# A Citrulline‐Functionalized Patch Coordinates Macrophage NRF2 Signaling and Arginine Metabolism for Tendon‐to‐Bone Repair

**DOI:** 10.1002/advs.78630

**Published:** 2026-11-09

**Authors:** Yuhao Yu, Wencai Liu, Xinyue Yang, Hanzhe Zhang, Hui Xu, Xin Wang, Weimin Lin, Xiping Jiang, Chenrui Yuan, Yifei Wang, Weilin Yu, Yamin Li, Wei Song, Yaohua He

**Affiliations:** ^1^ Department of Orthopedic Surgery Shanghai Sixth People's Hospital Affiliated to Shanghai Jiao Tong University School of Medicine Shanghai China; ^2^ Institute of Orthopaedic and Musculoskeletal Science University College London Royal National Orthopaedic Hospital London UK

**Keywords:** arginine metabolism, macrophage immunometabolism, NRF2 signaling, regenerative biomaterials, rotator cuff repair, tendon‐to‐bone interface

## Abstract

Structural retear remains common after rotator cuff repair. Persistent inflammation and metabolic dysfunction at the enthesis may impair healing, yet current augmentations rarely address this immunometabolic state. Exploratory single‐cell profiling shows a higher proportion of M1‐like cells among recovered macrophages in injured supraspinatus than in non‐injured semitendinosus specimens (three donors per group). Metabolomics of polarized macrophages identifies glutathione redox imbalance and reduced arginine and proline abundance in the M1 state, supporting redox regulation and arginine metabolism as complementary targets. In ACGQD@pPLG, citrulline‐functionalized, glutathione‐derived carbon dots are encapsulated in an H_2_O_2_‐responsive membrane coating from ultraviolet‐treated tendon‐derived stem cells and embedded in aligned core–shell electrospun fibers. In macrophages, the patch activates nuclear factor erythroid 2‐related factor 2 (NRF2) signaling and increases argininosuccinate synthetase 1 (ASS1) expression and arginine‐associated metabolite abundance. ASS1 knockdown attenuates CD206 induction, whereas NRF2 knockdown reduces HO‐1 and NQO1 induction. In rats, the patch improves enthesis collagen organization and increases ultimate stress, stiffness, and load to failure to 1.6–2.5 times repair‐only values at 8 weeks. Rabbit enthesis histology also improves at 10 weeks. These results support coordinated modulation of macrophage redox signaling and arginine metabolism as a local strategy for tendon‐to‐bone repair.

## Graphical abstract

A locally implanted patch combines citrulline‐functionalized carbon dots, a thioketal‐containing membrane coating from tendon‐derived stem cells, and aligned core–shell fibers. In macrophages, the patch reduces inflammatory markers and enhances antioxidant signaling through nuclear factor erythroid 2‐related factor 2 (NRF2); in rats and rabbits, it improves tendon‐to‐bone repair.

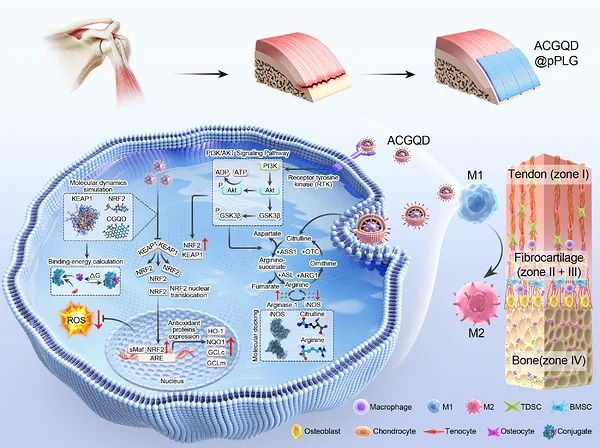

## 1 Introduction

Resolution of tissue injury involves a shift from proinflammatory toward reparative macrophage states [1, 2]. Macrophage metabolism shapes this shift, particularly through arginine metabolism. In M1‐like cells, arginine is consumed by inducible nitric oxide synthase (iNOS) to produce nitric oxide and citrulline, supporting host defense [3, 4]. In M2‐like cells, arginine is redirected through arginase 1 to ornithine and downstream polyamine and proline synthesis, pathways associated with tissue rebuilding [4]. Cytosolic argininosuccinate synthetase 1 (ASS1) and argininosuccinate lyase (ASL) recycle citrulline back to arginine and sustain substrate availability under prolonged demand [4]. Proinflammatory macrophages can also accumulate reactive oxygen species (ROS), which influence their activation state [5]. Endogenous antioxidant responses are governed by the Kelch‐like ECH‐associated protein 1 (KEAP1)–nuclear factor erythroid 2‐related factor 2 (NRF2) axis, with phosphoinositide 3‐kinase/protein kinase B (PI3K/AKT) signaling contributing to NRF2 stabilization [5, 6]. Whether concurrent modulation of redox signaling and arginine metabolism improves tendon‐to‐bone healing after rotator cuff repair remains unresolved.

Rotator cuff tear is a common musculoskeletal disorder whose prevalence increases with age [7, 8]. Structural failure still occurs following rotator cuff repair, particularly for large and massive tears, underscoring the limitations of surgical reconstruction alone [9, 10]. Biological augmentation may improve tendon integrity in selected settings, yet evidence for sustained functional benefit remains inconsistent [11]. Histopathological evaluation and recent single‐cell analyses have revealed proinflammatory and profibrotic macrophage states, tenocyte senescence, and disorganized matrix remodeling in injured tendons [12, 13]. Experimental studies have also identified fibrocartilage progenitor populations at the enthesis [14]. The redox and arginine‐metabolic features of macrophages in injured tendons remain incompletely characterized in clinical tissue. Defining these features could guide the design of local biological augmentation strategies for the tendon‐to‐bone interface.

Several biomaterial‐based augmentation strategies have been explored for tendon‐to‐bone repair. Growth factor–laden scaffolds deliver osteogenic, chondrogenic, or tenogenic cues [15, 16]. Engineered patches and scaffolds also provide cellular, immune, and structural cues for repair [17, 18, 19, 20, 21, 22, 23, 24]. Antioxidant nanozymes scavenge ROS and help reduce oxidative stress [25, 26]. Biomimetic platforms that combine cell‐membrane coatings with stimulus‐responsive release have been investigated for inflammatory disorders [27], motivating their integration with scaffolds for the inflamed tendon–bone niche. A platform that locally modulates redox signaling and arginine metabolism, while providing topographic guidance and local delivery at the tendon–fibrocartilage–bone interface, could support tendon‐to‐bone repair.

To combine these functions, we developed ACGQD@pPLG, a composite patch with three engineered components for local redox and arginine‐metabolic modulation and topographic guidance. Single‐cell RNA sequencing of clinical tendon specimens showed differences in macrophage‐state composition and sparse detection of ASS1/ASL transcripts. Widely targeted metabolomics of polarized macrophages identified glutathione redox imbalance and altered arginine‐related metabolites. First, citrulline‐conjugated glutathione‐derived carbon dots (CGQDs) were designed to buffer oxidative stress and modulate ASS1‐associated arginine metabolism. Second, CGQDs were encapsulated in a thioketal‐containing membrane preparation (ApoTK) from unsorted UV‐treated tendon‐derived stem cells (TDSCs) to form ACGQD nanoparticles with H2O2‐responsive release. Third, ACGQD was embedded in an aligned poly(L‐lactic acid) (PLLA)‐shell/gelatin methacryloyl (GelMA)‐core electrospun fiber matrix (pPLG) for sustained local delivery and topographic guidance. Cellular analyses showed macrophage‐marker changes associated with NRF2 signaling and arginine‐related metabolite changes, and rat tissue staining showed corresponding marker responses. Rat studies demonstrated improved histological, biomechanical, and gait outcomes, while rabbit histology showed improved tendon‐to‐bone interface repair (Scheme 1).

**SCHEME 1:**
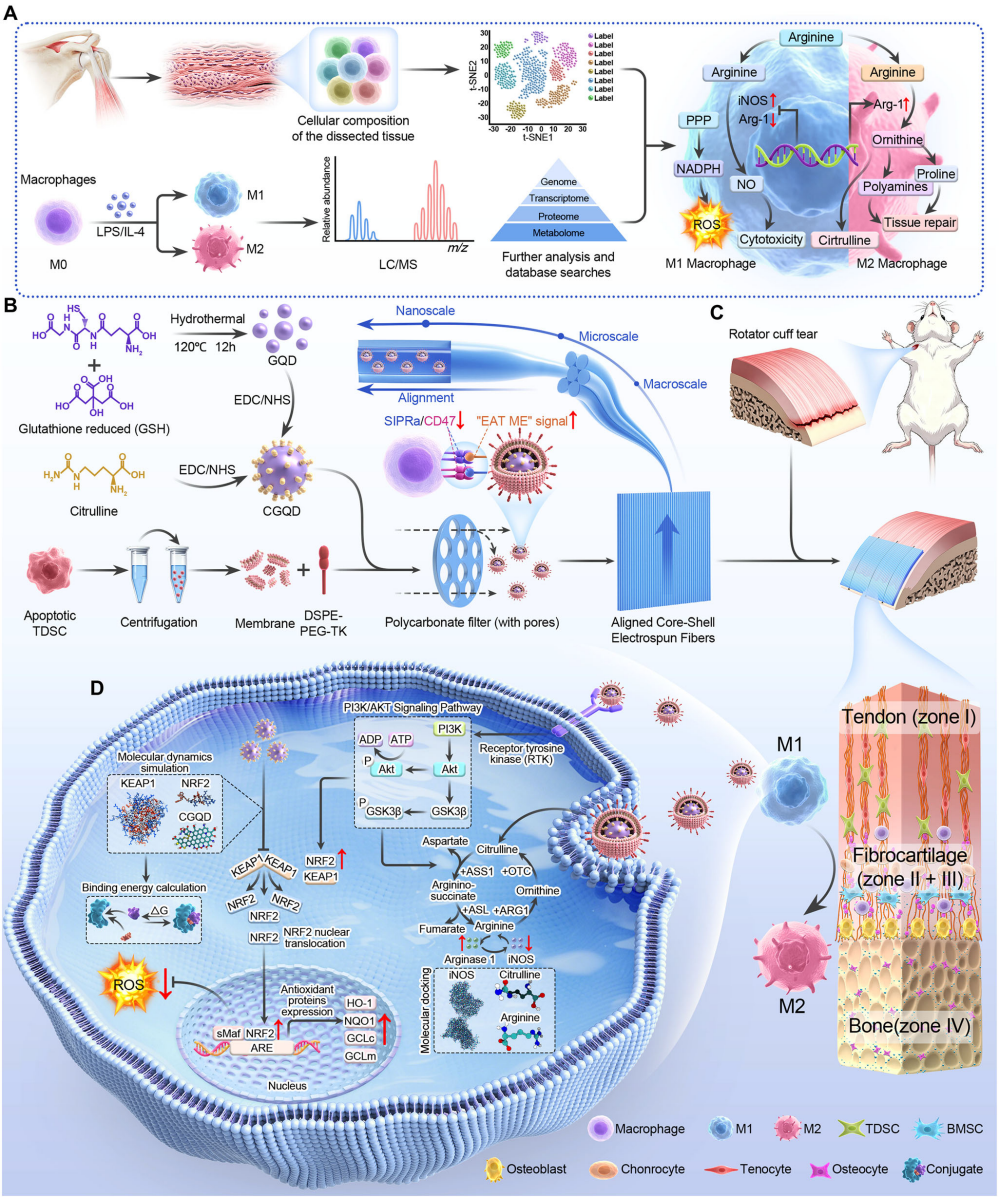
Design and evaluated responses of ACGQD@pPLG. (A) Exploratory single‐cell profiling of clinical tendon specimens and metabolomics of polarized THP‐1‐derived macrophages informed examination of redox regulation and arginine‐associated metabolism. (B) Glutathione‐derived carbon dots (GQD) were functionalized with L‐citrulline to form CGQD. CGQD was incorporated into a DSPE–PEG–TK‐containing membrane preparation from unsorted UV‐treated TDSCs to form ACGQD, which was then loaded into aligned PLLA‐shell/GelMA‐core fibers (pPLG). (C) Local patch implantation in the rat rotator cuff repair model. (D) Working model relating macrophage‐marker and NRF2‐related responses to measured metabolite abundance and repair outcomes.

## 2 Results

### 2.1 Clinical Tendon Profiles and Macrophage Metabolomics Inform Patch Design

We performed single‐cell RNA sequencing (scRNA‐seq) on supraspinatus tear‐stump tissue from three patients with rotator cuff tears (TN) and on semitendinosus tendons from three different patients undergoing anterior cruciate ligament reconstruction (NT). The combined analysis resolved seven major cell populations (Figure 1A,C and Figure S1), and macrophage subclustering identified M1‐like, M2‐like, and proliferating states (Figure 1B). Donor‐resolved embeddings and proportions (Figures S2 and S3) showed that M1‐like cells accounted for 8.2%–15.4% of macrophages in NT samples and 48.9%–56.4% in TN samples, whereas M2‐like cells accounted for 61.0%–73.2% and 42.9%–44.6%, respectively. The *ASS1* and *ASL* feature plots showed sparse transcript detection (Figure 1D and Figures S4 and S5). Immunofluorescence of the clinical specimens showed stronger iNOS and 4‐hydroxynonenal (4‐HNE) signals in injured tendons, no significant difference in ASS1 between the specimen groups (*p* = 0.4532), and a lower CD68 signal in injured tendons (*p* = 0.0493; Figure 1E,F and Figure S6). CD68 staining intensity reflects section‐level CD68 immunoreactivity, whereas the scRNA‐seq proportions describe the composition of the recovered macrophage states. Staining with an oxidant‐sensitive probe also revealed oxidant‐associated fluorescence in an injured tendon specimen (Figure S7). CellChat analysis further indicated differences in inferred macrophage–stromal communication between the two specimen groups (Figure 1G,H and Figure S8).

**FIGURE 1:**
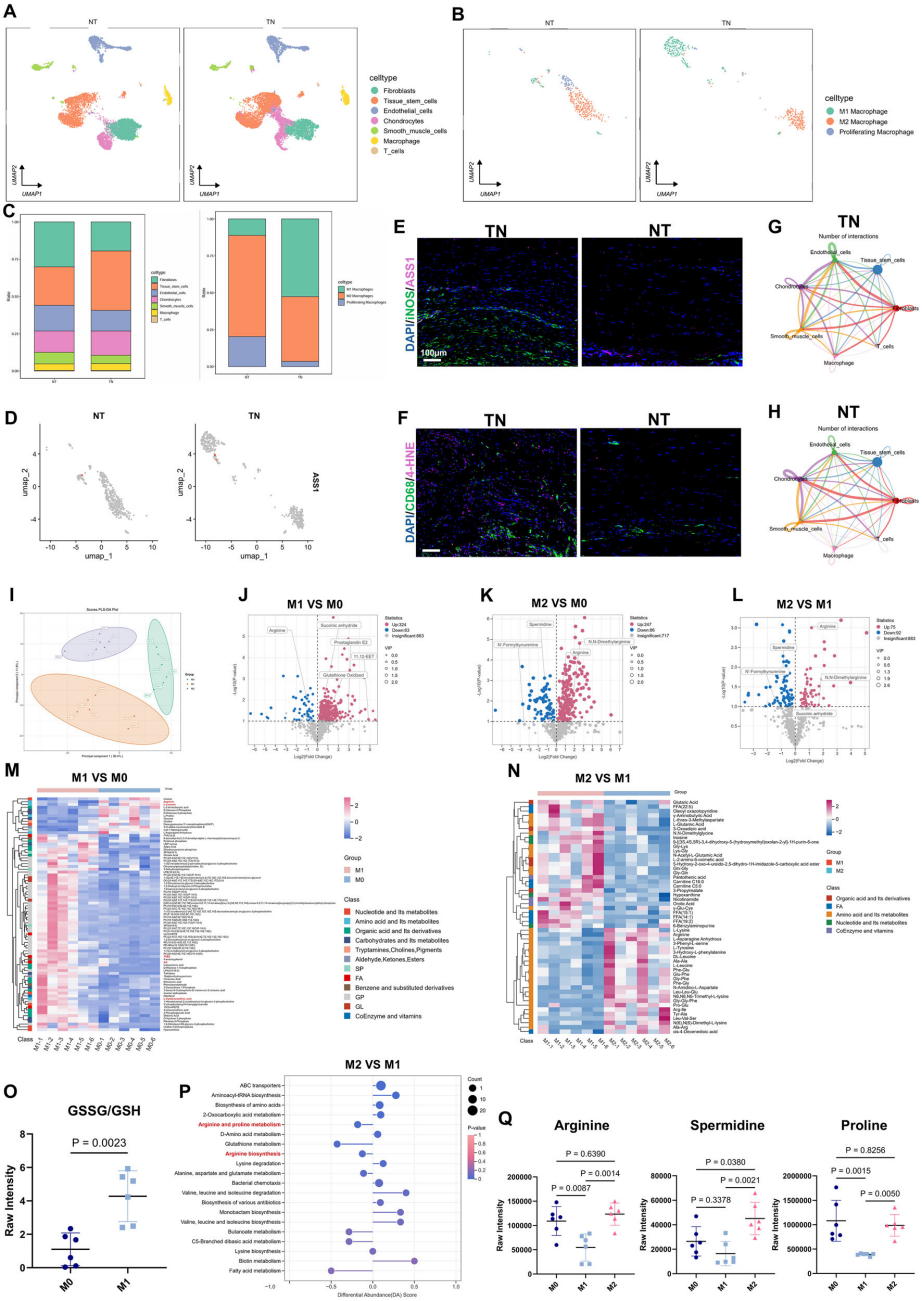
Clinical tendon profiling and macrophage metabolomics inform patch design. (A) UMAP of scRNA‐seq from non‐injured comparison tendon (NT) and injured tendon (TN) specimens, colored by major cell type. (B) Macrophage subclustering showing M1‐like, M2‐like, and proliferating states. (C) Cell‐type proportions in NT and TN: all populations (left) and macrophage subtypes (right). (D) UMAP feature plot of *ASS1* in the macrophage compartment. (E, F) Representative immunofluorescence of injured (TN) and non‐injured comparison (NT) tendon: DAPI (blue), iNOS (green), and ASS1 (magenta) (E); DAPI (blue), CD68 (green), and 4‐HNE (magenta) (F). Scale bar, 100 µm. (G, H) Cell–cell communication networks of injured tendon (TN; G) and non‐injured comparison tendon (NT; H); edge thickness, inferred interaction count. (I) PLS‐DA score plot of widely targeted metabolomics in M0, M1, and M2 macrophages. (J–L) Volcano plots of differentially abundant metabolites for M1 vs. M0 (J), M2 vs. M0 (K), and M2 vs. M1 (L). (M, N) Hierarchical clustering of differential metabolites for M1 vs. M0 (M) and M2 vs. M1 (N). (O) GSSG/GSH ratio in M0 and M1 macrophages (*p* = 0.0023). (P) KEGG pathway differential‐abundance scores for M2 vs. M1 macrophages. (Q) Intensities of arginine, spermidine, and proline in M0, M1, and M2 macrophages. Data are mean ± SD. The scRNA‐seq analysis of human tendon specimens included three donors per group; *n* = 6 for the metabolomics measurements (I–Q). Comparisons were made by two‐sided Student's *t*‐test (O) or one‐way ANOVA with Tukey's post hoc test (Q); *p* values are shown in the panels.

To characterize macrophage metabolic states, we performed widely targeted metabolomics on phorbol 12‐myristate 13‐acetate (PMA)‐differentiated THP‐1 macrophages in unpolarized (M0), M1‐polarized, and M2‐polarized states. Partial least squares discriminant analysis (PLS‐DA) separated the three states (Figure 1I), and pairwise comparisons identified 387 (M1 vs. M0), 333 (M2 vs. M0), and 167 (M2 vs. M1) differential metabolites (Figure 1J–N and Figures S9–S11). Two changes involved redox and arginine metabolism. First, the ratio of oxidized to reduced glutathione (GSSG/GSH), an indicator of oxidative state, was approximately 3.88‐fold higher in M1 than in M0 macrophages (4.28 ± 1.52 vs. 1.10 ± 0.98; Figure 1O), consistent with glutathione redox imbalance. Second, Kyoto Encyclopedia of Genes and Genomes (KEGG) analysis ranked arginine biosynthesis and arginine–proline metabolism among the most altered pathways (Figure 1P). Intracellular arginine and proline were approximately 49.86% and 64.06% lower in M1 than in M0 macrophages, whereas M2 macrophages contained approximately 2.26‐ and 2.54‐fold more arginine and proline, respectively, than M1 macrophages. Spermidine was elevated only in M2 macrophages (approximately 1.71‐fold relative to M0; Figure 1Q).

Together, the clinical profiles and macrophage metabolomics informed a patch design targeting redox regulation and arginine metabolism.

### 2.2 Engineering and Characterization of the ACGQD@pPLG Patch

The ACGQD@pPLG patch has three components: a citrulline‐functionalized, glutathione‐derived carbon‐dot core; a TDSC membrane coating containing a thioketal linker; and an aligned PLLA‐shell/GelMA‐core electrospun matrix.

CGQDs were prepared by coupling L‐citrulline to GQDs with 1‐ethyl‐3‐(3‐dimethylaminopropyl) carbodiimide/*N*‐hydroxysuccinimide (EDC/NHS) chemistry, and the resulting changes in surface chemistry were characterized (Figure 2A–D and Figures S12–S14 and S18). Transmission electron microscopy (TEM) showed approximately spherical particles, and dynamic light scattering (DLS) indicated hydrodynamic diameters of 3.22 nm for GQDs and 8.39 nm for CGQDs. In Fourier transform infrared (FTIR) spectra, the band near 1640 cm^−1^ was consistent with amide vibrations, with overlapping carbonyl and ureido contributions, and X‐ray photoelectron spectroscopy (XPS) indicated changes in nitrogen‐containing surface environments. The zeta potential was −30.28 ± 3.33 mV for GQDs, and the comparison with CGQDs showed no significant difference (*p* = 0.3578; Figure S18). Citrulline bears a neutral ureido side chain. Figure 2E depicts the proposed surface chemistry.

**FIGURE 2:**
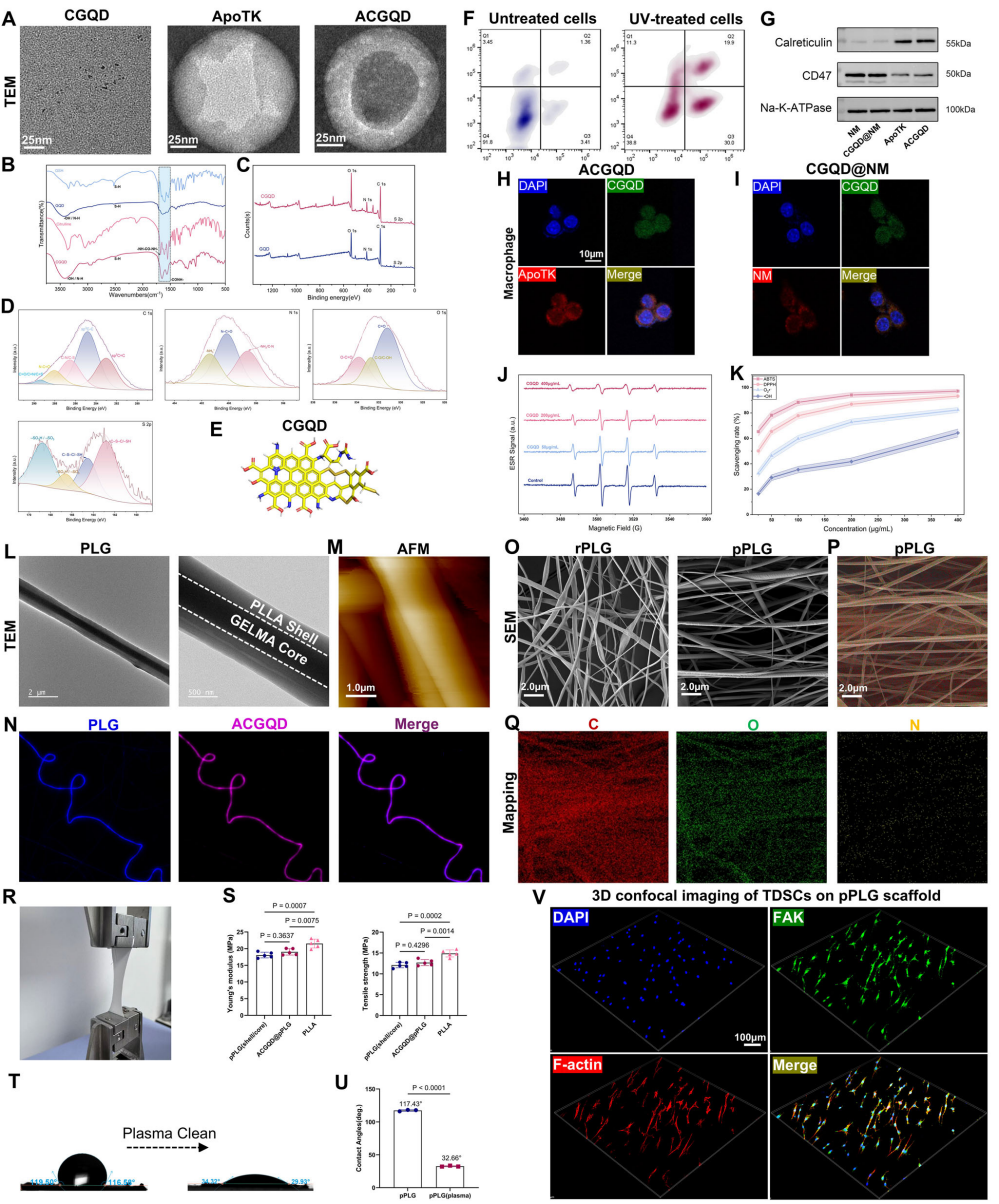
Engineering and characterization of the ACGQD@pPLG patch. (A) Representative transmission electron microscopy (TEM) images of CGQD, ApoTK, and ACGQD; scale bars, 25 nm. (B) Fourier transform infrared spectra of glutathione (GSH), GQD, L‐citrulline, and CGQD. (C) X‐ray photoelectron spectroscopy survey spectra of GQD and CGQD. (D) High‐resolution C 1s, N 1s, O 1s, and S 2p spectra of CGQD with fitted components. (E) Illustrative model of the proposed CGQD surface chemistry. (F) Annexin V/propidium iodide flow cytometry of untreated and UV‐treated TDSCs used for membrane preparation. The unsorted UV‐treated population was used for membrane preparation. (G) Representative immunoblots of calreticulin and CD47 in NM, CGQD@NM, ApoTK, and ACGQD; Na^+^/K^+^‐ATPase is the membrane‐protein loading control. NM denotes membranes from untreated TDSCs, and the corresponding densitometry is shown in Figure S17. (H, I) Confocal images of macrophages exposed to ACGQD (H) or CGQD@NM (I). DAPI, CGQD‐associated fluorescence, and DiD‐labeled membrane are shown in blue, green, and red, respectively; scale bar, 10 µm. (J) Electron paramagnetic resonance spectra at the indicated CGQD concentrations. (K) Scavenging percentages for ABTS•^+^, DPPH•, O_2_•^−^, and •OH over the tested CGQD concentration range. (L) TEM images of a core–shell PLG fiber; scale bars, 2 µm and 500 nm. (M) Atomic force microscopy topography of a pPLG fiber; scale bar, 1 µm. (N) Confocal images of fluorescently labeled scaffold fibers (blue), ACGQD (magenta), and the merged view, showing cargo distribution in the cell‐free scaffold. (O) Scanning electron microscopy of randomly oriented PLG (rPLG) and aligned PLG (pPLG) fibers. (P) Color‐enhanced view of aligned pPLG fibers. Scale bars in O and P, 2 µm. (Q) Energy‐dispersive X‐ray spectroscopy maps of C, O, and N in the scaffold. (R) Tensile‐testing setup. (S) Young's modulus (left) and tensile strength (right) of pPLG, ACGQD@pPLG, and PLLA. (T, U) Water‐contact‐angle images before and after plasma treatment (T) and quantification (U). (V) 3D confocal images of TDSCs on pPLG; DAPI, FAK, and F‐actin are shown in blue, green, and red, respectively. Scale bar, 100 µm. Data are mean ± SD; *n* = 5 specimens per formulation in S and *n* = 3 for the other quantitative analyses. Comparisons were made by one‐way ANOVA with Tukey's post hoc test (S) or two‐sided Student's *t*‐test (U); *p* values are shown in the panels.

We compared free L‐citrulline, GQDs, and CGQDs at the same nominal mass concentration (50 µg mL^−^^1^) for 24 h in lipopolysaccharide (LPS)‐stimulated macrophages. Free L‐citrulline decreased iNOS and increased CD206 relative to LPS alone, unmodified GQDs had little effect on CD206, and CGQDs produced the largest change in both markers (Figure S28). These results support iNOS and CD206 modulation by the citrulline‐functionalized formulation under the tested mass‐based dosing conditions.

CGQDs were then encapsulated in ApoTK, a DSPE–PEG–TK‐containing membrane preparation from UV‐treated TDSCs, to form ACGQD. UV irradiation induced apoptosis in approximately 51.7% of donor TDSCs, compared with approximately 6.0% of untreated cells (Figure 2F and Figure S15). The unsorted UV‐treated population yielded a membrane preparation containing contributions from both apoptotic and non‐apoptotic TDSCs. Compared with membranes from untreated TDSCs (NM), ApoTK and ACGQD showed higher calreticulin and lower CD47 levels (Figure 2G and Figure S17). Membrane coating raised the zeta potential to −15.61 ± 2.74 mV for ApoTK and −16.24 ± 2.83 mV for ACGQD, and the assembled ACGQD had a hydrodynamic diameter of 154.2 nm (Figures S13 and S18). Macrophages exposed to ACGQD showed higher CGQD‐associated fluorescence than those exposed to CGQD@NM (Figure 2H,I and Figure S19), and DiD fluorescence from the labeled coating increased progressively between 1 and 12 h of incubation (Figure S20). Both assays quantified cell‐associated fluorescence.

Electron paramagnetic resonance (EPR) spectroscopy showed concentration‐dependent radical scavenging by CGQDs (Figure 2J). At 400 µg mL^−1^, CGQDs scavenged approximately 97.18% of 2,2′‐azino‐bis (3‐ethylbenzothiazoline‐6‐sulfonic acid) radical cations (ABTS•^+^), 93.24% of 2,2‐diphenyl‐1‐picrylhydrazyl radicals (DPPH•), 82.36% of superoxide anions (O_2_•^−^), and 64.27% of hydroxyl radicals (•OH) (Figure 2K). Over 28 days, CGQD release from ACGQD was greater in H_2_O_2_‐containing media than in phosphate‐buffered saline (PBS) and increased with H_2_O_2_ concentration (Figure S22), confirming H_2_O_2_‐responsive release in vitro.

For local delivery, ACGQD was loaded into the aligned PLLA‐shell/GelMA‐core electrospun matrix. TEM showed the core–shell fiber architecture, and atomic force microscopy (AFM) characterized the fiber topography (Figure 2L,M). Scanning electron microscopy (SEM) showed aligned fibers in pPLG and randomly oriented fibers in the reference mat (rPLG; Figure 2O,P); aligned electrospun fibers have been used to approximate the anisotropic organization of rotator cuff tendon [28]. Confocal imaging and elemental mapping showed ACGQD‐associated fluorescence along the fibers and the distribution of C, O, and N within the scaffold (Figure 2N,Q). In tensile tests, Young's modulus and tensile strength did not differ significantly between pPLG and ACGQD@pPLG, whereas both values were higher for PLLA (Figure 2R,S). After incubation in PBS at 37°C, elongation at break was similar for pPLG and ACGQD@pPLG and slightly higher for PLLA (Figure S21). Plasma treatment reduced the water contact angle from 117.43° to 32.66° (Figure 2T,U), and TDSCs cultured on pPLG adopted an elongated morphology, with focal adhesion kinase (FAK) and F‐actin signals extending along the fiber direction (Figure 2V). Compared with PLLA fibers, PLG fibers showed greater cumulative ACGQD release over 28 days and lost mass more rapidly during 120 days of incubation (Figures S23 and S24).

### 2.3 ACGQD@pPLG Modifies Macrophage Markers and Reduces Oxidative Stress

In Figures 3, 4, 5, 6, 7, 8, 9, Control denotes the corresponding no‐patch (in vitro) or suture‐repair‐only (in vivo) condition, and pPLG, ApoTK, CGQD, and ACGQD denote patches that were unloaded or loaded with ApoTK, CGQDs, or ACGQD, respectively. For cellular assays, patches were placed in Transwell inserts (Figures 3A and 6A); for animal studies, they were implanted at the repair site. PMA‐differentiated THP‐1 macrophages were stimulated with LPS and co‐cultured with the patches; transcripts were analyzed at 12 h and protein markers at 24 h (Figure 3A). In cytocompatibility tests, Cell Counting Kit‐8 (CCK‐8) absorbance, a measure of metabolic activity, increased over 5 days in all groups. No formulation reduced the signal in THP‐1 macrophages or bone marrow‐derived mesenchymal stem cells (BMSCs) (Figure S25), and TDSC absorbance did not differ significantly between groups at any time point (Figure S26).

**FIGURE 3:**
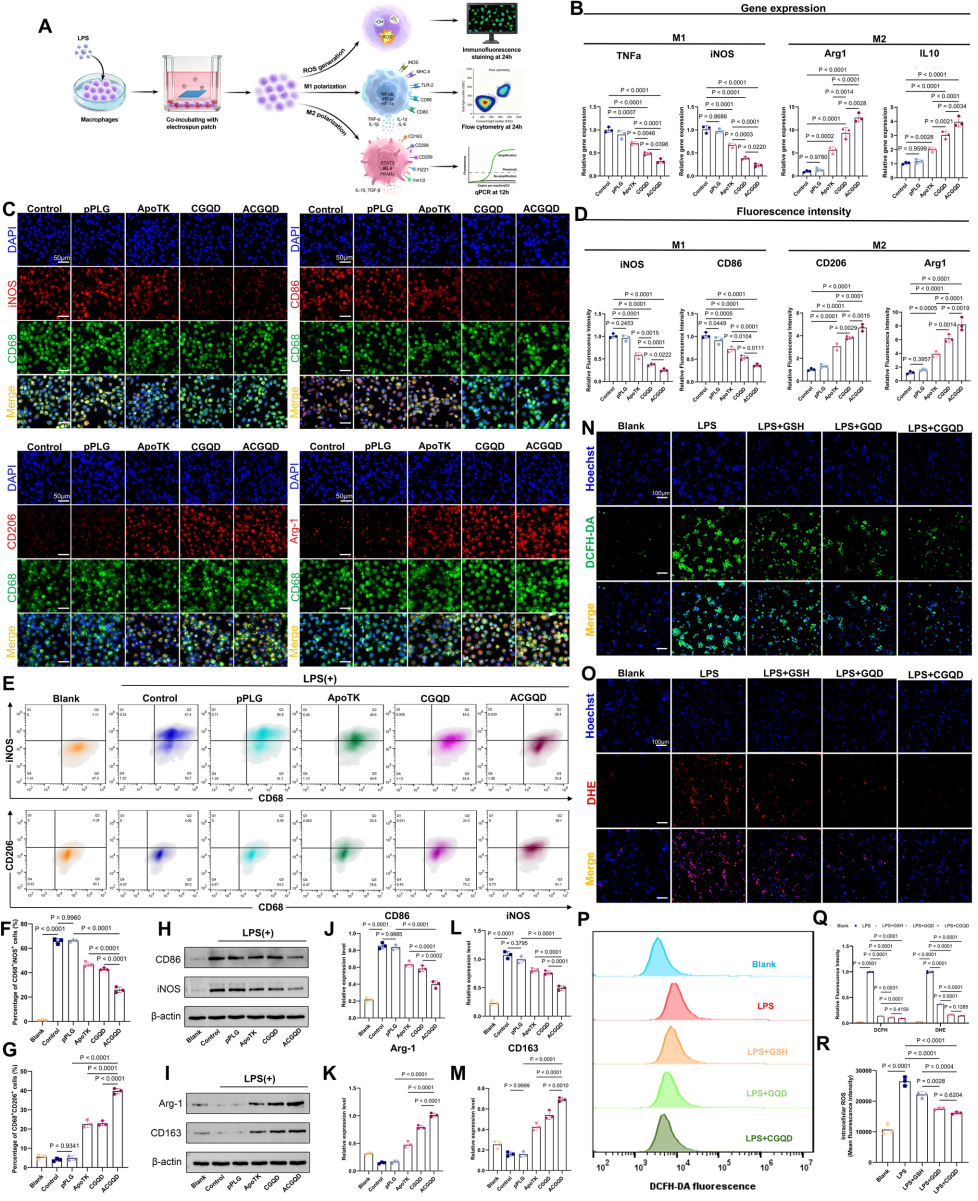
ACGQD@pPLG modifies macrophage markers and reduces oxidative stress. PMA‐differentiated THP‐1 macrophages were stimulated with LPS and co‐cultured in Transwell inserts without a patch (Control) or with the indicated electrospun patches (pPLG, ApoTK, CGQD, or ACGQD); unstimulated cells served as Blank. Sampling times for each readout are indicated in (A). (A) Schematic of the co‐culture and downstream readouts. (B) RT‐qPCR of M1 markers (*TNF*, *NOS2*) and M2 markers (*ARG1*, *IL10*). (C) Immunofluorescence of CD68 (green) with iNOS, CD86, CD206, or Arg‐1 (red); nuclei are stained with DAPI (blue). Scale bar, 50 µm. (D) Relative fluorescence intensities from (C). (E) Flow‐cytometric density plots of CD68 with iNOS (upper row) or CD206 (lower row) in the Blank, Control, pPLG, ApoTK, CGQD, and ACGQD conditions. (F, G) Quantification of CD68^+^iNOS^+^ (F) and CD68^+^CD206^+^ (G) percentages. (H, I) Western blots for CD86 and iNOS (H) or Arg‐1 and CD163 (I); β‐actin loading control. (J–M) Western blot quantification of CD86 (J), Arg‐1 (K), iNOS (L), and CD163 (M). (N, O) Fluorescence images of macrophages stained with Hoechst and DCFH‐DA (N) or Hoechst and DHE (O) in the Blank, LPS, LPS + GSH, LPS + GQD, and LPS + CGQD groups, in which GSH, GQD, and CGQD denote the patch cargo; scale bar, 100 µm. (P) Flow‐cytometric DCFH‐DA histograms. (Q) Quantification of DCFH‐DA and DHE fluorescence from (N) and (O). (R) Flow‐cytometric DCFH‐DA mean fluorescence intensity from (P). Data are mean ± SD; *n* = 3 biologically independent samples per group. Comparisons were made by one‐way ANOVA with Tukey's post hoc test; *p* values are shown in the panels.

**FIGURE 4:**
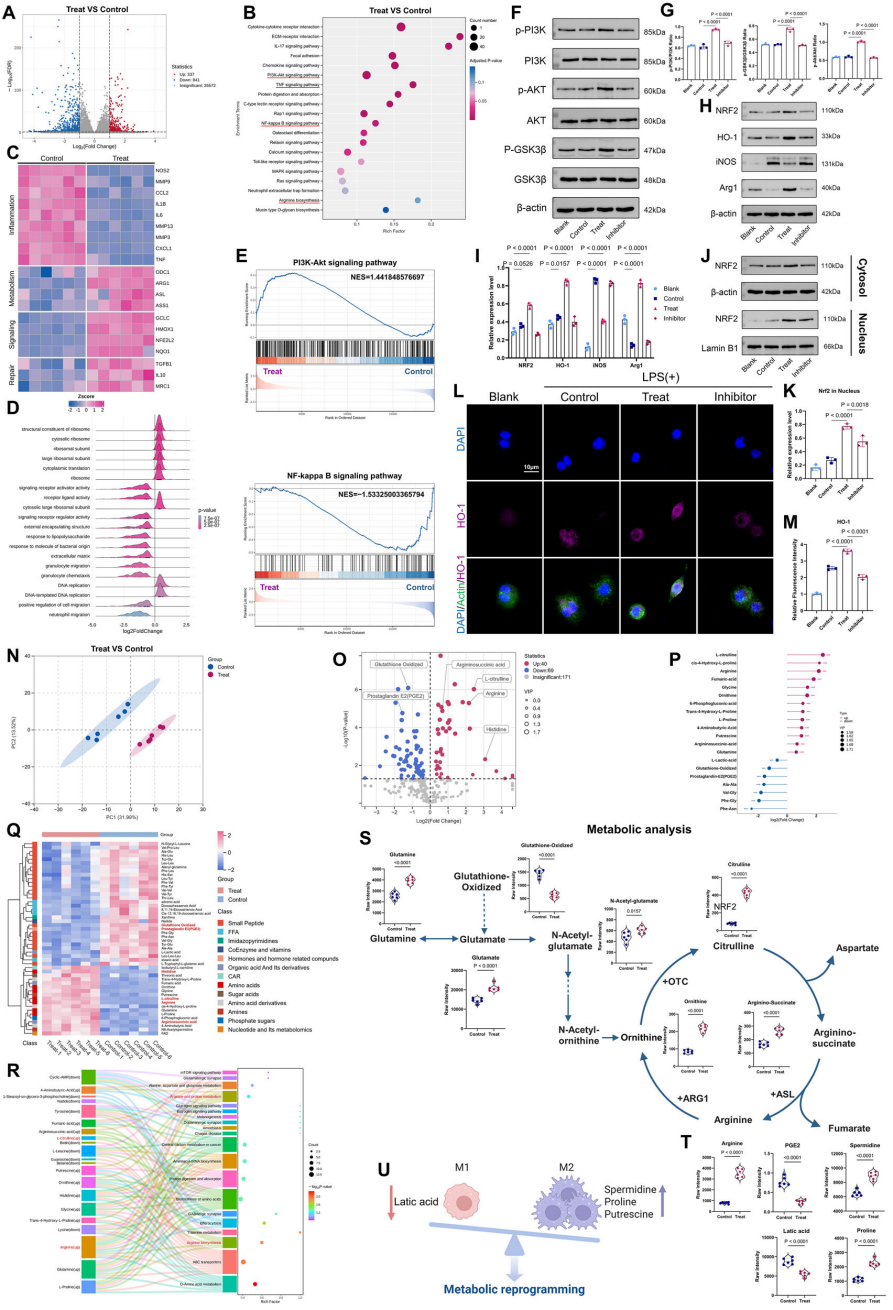
NRF2‐related signaling and arginine‐associated metabolite changes in treated macrophages. PMA‐differentiated THP‐1 macrophages were treated under four conditions: Blank (no LPS), Control (LPS), Treat (LPS + ACGQD@pPLG), and Inhibitor (Treat + 10 µM LY294002, 1 h pretreatment). (A) Volcano plot of differentially expressed transcripts in Treat vs. Control (up 337, down 841). (B) KEGG pathway enrichment. (C) Hierarchical clustering of inflammation, metabolism, signaling, and repair genes. (D) GO ridge plot. (E) GSEA for PI3K–Akt (NES = 1.442) and NF‐κB (NES = −1.533). (F, G) Western blots (F) and quantification (G) of p‐PI3K/PI3K, p‐AKT/AKT, and p‐GSK3β/GSK3β; β‐actin, loading control. (H, I) Western blots (H) and quantification (I) of NRF2, HO‐1, iNOS, and Arg‐1; β‐actin, loading control. (J, K) Cytoplasmic–nuclear fractionation Western blots of NRF2 (J) and nuclear NRF2 quantification (K); β‐actin and Lamin B1 are the cytoplasmic and nuclear loading controls, respectively. (L, M) Confocal images of HO‐1 (magenta), DAPI (blue), and F‐actin (green) (L) and HO‐1 fluorescence quantification (M); scale bar, 10 µm. (N) Principal component analysis of metabolomics. (O) Volcano plot of differential metabolites (up 40, down 69). (P) Top differential metabolites ranked by log_2_ fold change. (Q) Heatmap of differential metabolites with class annotation. (R) KEGG pathway enrichment of differential metabolites. (S) Urea–arginine cycle schematic with violin plots of glutamine, glutamate, GSSG, N‐acetyl‐glutamate, citrulline, ornithine, and argininosuccinate. (T) Relative abundance of arginine, PGE2, spermidine, lactate, and proline. (U) Working model relating measured metabolite abundance to macrophage‐marker responses. Data are mean ± SD. *n* = 3 for G, I, K, and M; *n* = 6 for the omics measurements in A–E and N–T. Comparisons were made by two‐sided Student's *t*‐test for two‐group metabolite data (S–T) and by one‐way ANOVA with Tukey's post hoc test for multigroup molecular measurements (G, I, K, M); *p* values are shown in the panels.

**FIGURE 5:**
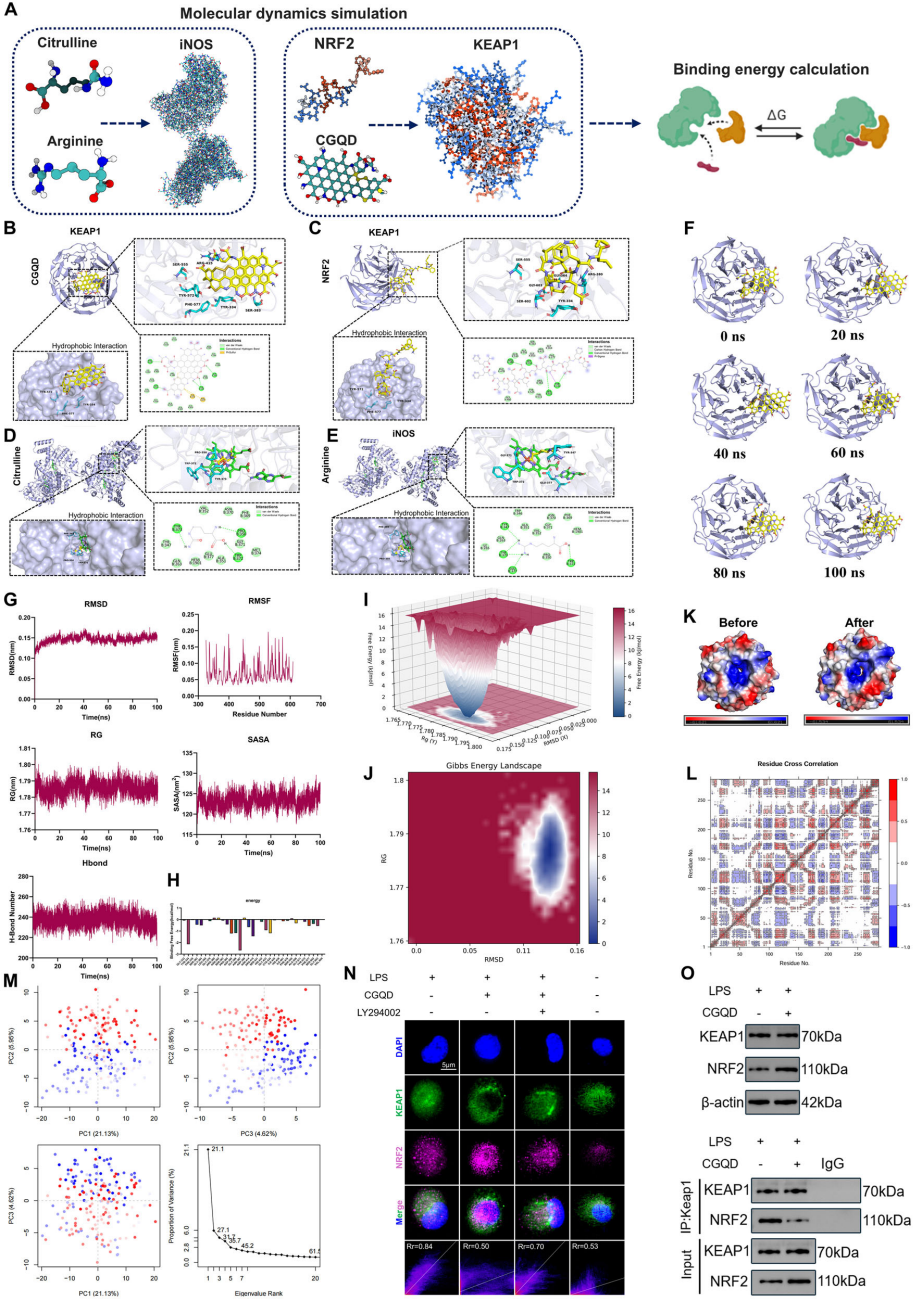
Computational interaction models and cellular KEAP1–NRF2 association. (A) Computational workflow: a selected CGQD representation and an NRF2‐derived ligand model docked to KEAP1; citrulline and arginine docked into iNOS; 100‐ns all‐atom molecular dynamics simulations and per‐residue analyses. (B) Docking of the selected CGQD representation into the KEAP1 Kelch domain (docking score = −9.608 kcal mol^−^^1^); key residues Ser555, Arg415, Tyr572, Phe577, Tyr334, and Ser383, with hydrophobic‐interaction surface and 2D interaction map below. (C) Docking of the NRF2‐derived ligand model into KEAP1 (docking score = −9.033 kcal mol^−^^1^); key residues Ser555, Gly364, Gly603, Arg380, Tyr334, and Ser602. (D) Docking of citrulline into iNOS (docking score = −6.005 kcal mol^−^^1^); key residues Pro350, Trp372, and Tyr373. (E) Docking of arginine into iNOS (docking score = −5.938 kcal mol^−^^1^); key residues Tyr347, Trp372, Tyr373, and Glu377. (F) Snapshots of the CGQD–KEAP1 complex at 0, 20, 40, 60, 80, and 100 ns. (G) Backbone RMSD, per‐residue RMSF, radius of gyration (Rg), solvent‐accessible surface area (SASA), and number of hydrogen bonds. (H) Per‐residue binding‐energy decomposition. (I, J) Free‐energy landscape projected onto RMSD and Rg, shown as a 3D surface (I) and a 2D contour map (J). (K) Electrostatic surface potential of KEAP1 alone (Before) and in the modeled CGQD complex (After). (L) Residue cross‐correlation matrix. (M) Principal component analysis (PC1, 21.13%; PC2, 5.95%; PC3, 4.62%) with cumulative‐variance plot. (N) Confocal immunofluorescence of KEAP1 (green), NRF2 (magenta), and DAPI (blue) in the LPS, LPS + CGQD, LPS + CGQD + LY294002, and unstimulated groups; Pearson's *R* values are indicated. Scale bar, 5 µm. (O) Western blots of total KEAP1 and NRF2 (top) and co‐immunoprecipitation of NRF2 with anti‐KEAP1 (bottom); β‐actin and IgG serve as the loading and immunoprecipitation controls, respectively. *n* = 3 biologically independent samples per group for (N, O).

**FIGURE 6:**
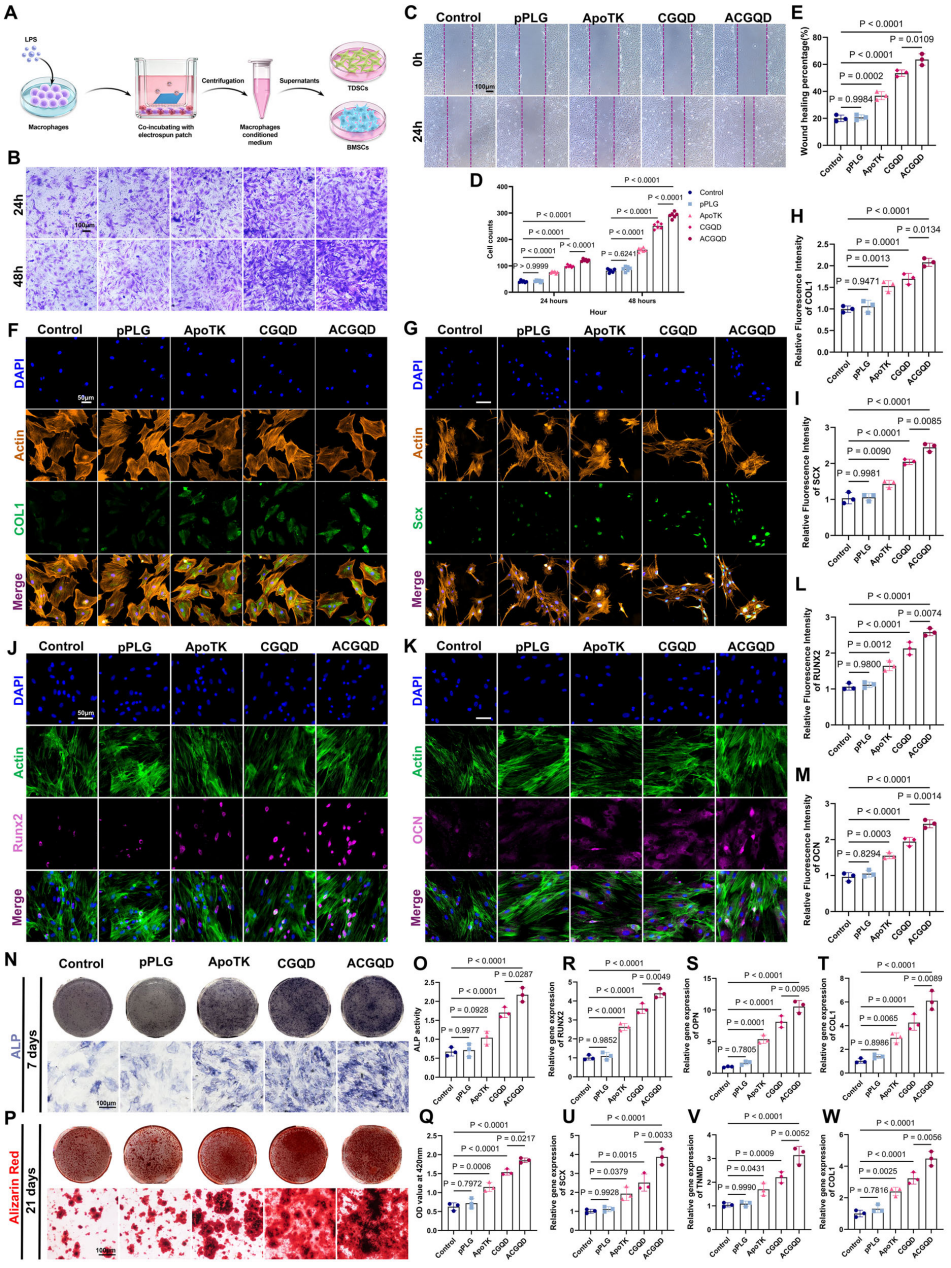
Conditioned medium from treated macrophages supports tendon and bone marrow stromal‐cell responses. PMA‐differentiated THP‐1 macrophages were stimulated with LPS and incubated without a patch (Control) or with the indicated electrospun patches (pPLG, ApoTK, CGQD, or ACGQD) in Transwell inserts for 48 h. After washing, macrophages were cultured in serum‐free medium for 24 h to collect conditioned medium, which was transferred to TDSCs or BMSCs. (A) Schematic of the conditioned‐medium workflow. (B) Transwell images of TDSCs at 24 h (top) and 48 h (bottom); scale bar, 100 µm. (C) Scratch‐wound images of TDSCs at 0 h (top) and 24 h (bottom); scale bar, 100 µm. (D) Transmigrated TDSC counts at 24 h and 48 h. (E) Wound‐healing percentage of TDSCs at 24 h. (F, G) Immunofluorescence of TDSCs co‐stained with DAPI (blue), F‐actin (orange), and COL1 (green; F) or SCX (green; G); scale bar, 50 µm. (H, I) Quantification of COL1 (H) and SCX (I). (J, K) Immunofluorescence of BMSCs co‐stained with DAPI (blue), F‐actin (green), and RUNX2 (magenta; J) or OCN (magenta; K); scale bar, 50 µm. (L, M) Quantification of RUNX2 (L) and OCN (M). (N, O) ALP staining at day 7 (N) and ALP activity quantification (O); scale bar, 100 µm. (P, Q) Alizarin Red at day 21 (P) and OD_420_ quantification (Q); scale bar, 100 µm. (R–T) qPCR of *RUNX2* (R), *OPN* (S), and *COL1A1* (T) in BMSCs. (U–W) qPCR of *SCX* (U), *TNMD* (V), and *COL1A1* (W) in TDSCs. Data are mean ± SD; *n* = 6 independent replicates for (D) and *n* = 3 independent replicates for the other quantitative analyses. Comparisons were made by one‐way ANOVA with Tukey's post hoc test; *p* values are shown in the panels.

**FIGURE 7:**
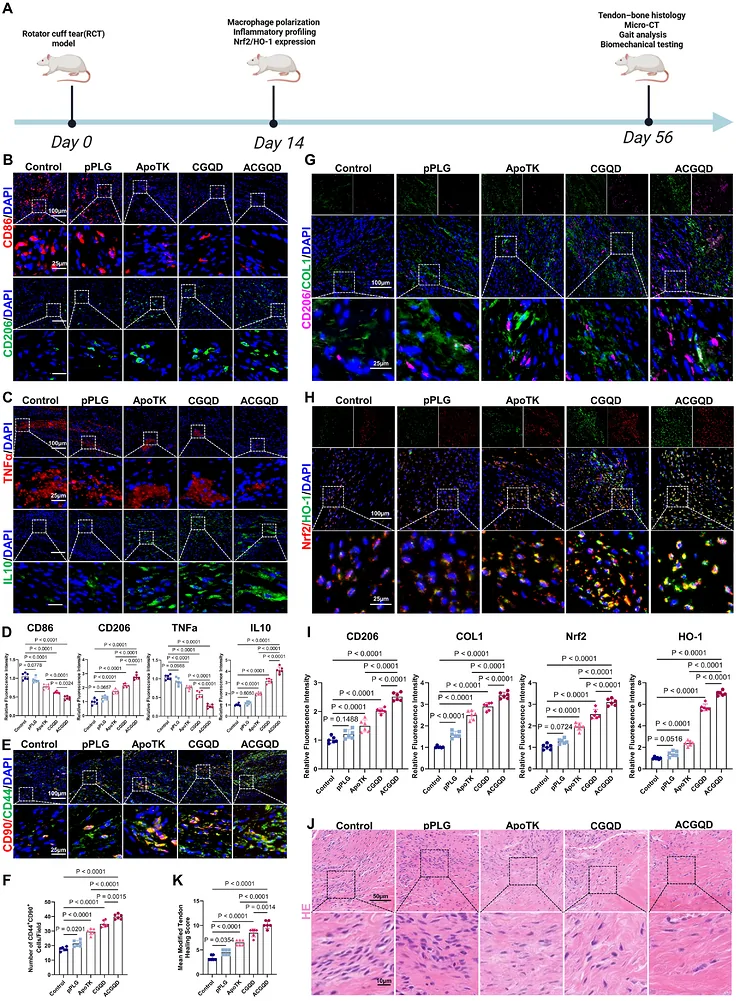
Early tissue responses after ACGQD@pPLG‐assisted rotator cuff repair in rats. Control denotes suture repair only; pPLG denotes the unloaded aligned scaffold. ApoTK, CGQD, and ACGQD denote the corresponding patch‐loaded formulations. Tissue staining was assessed at day 14. (A) Study timeline showing the early and 8‐week evaluations. (B) CD86 (red) and CD206 (green) immunofluorescence, displayed separately with DAPI (blue). (C) TNF‐α (red) and IL‐10 (green) immunofluorescence, displayed separately with DAPI. (D) Relative fluorescence intensities of CD86, CD206, TNF‐α, and IL‐10. (E) CD90 (red)/CD44 (green) co‐staining with DAPI. (F) Number of CD44^+^CD90^+^ cells per field. (G) CD206 (magenta)/COL I (green) co‐staining with DAPI. Spatial overlap indicates the distribution of CD206‐positive cells within collagen‐rich tissue. (H) NRF2 (red)/HO‐1 (green) co‐staining with DAPI. (I) Relative fluorescence intensities of CD206, COL I, NRF2, and HO‐1. Scale bars in B, C, E, G, and H, 100 µm in the overview images and 25 µm in the enlargements. (J) Hematoxylin and eosin staining of the repaired tendon‐to‐bone interface; scale bars, 50 µm and 10 µm. (K) Modified tendon healing score; criteria are provided in Table S1. Dashed boxes indicate enlarged regions. Relative fluorescence measurements are expressed against the repair‐only Control reference. Data are mean ± SD; *n* = 6 independent animals per group. Comparisons were made by one‐way ANOVA with Tukey's post hoc test; *p* values are shown in the panels.

**FIGURE 8:**
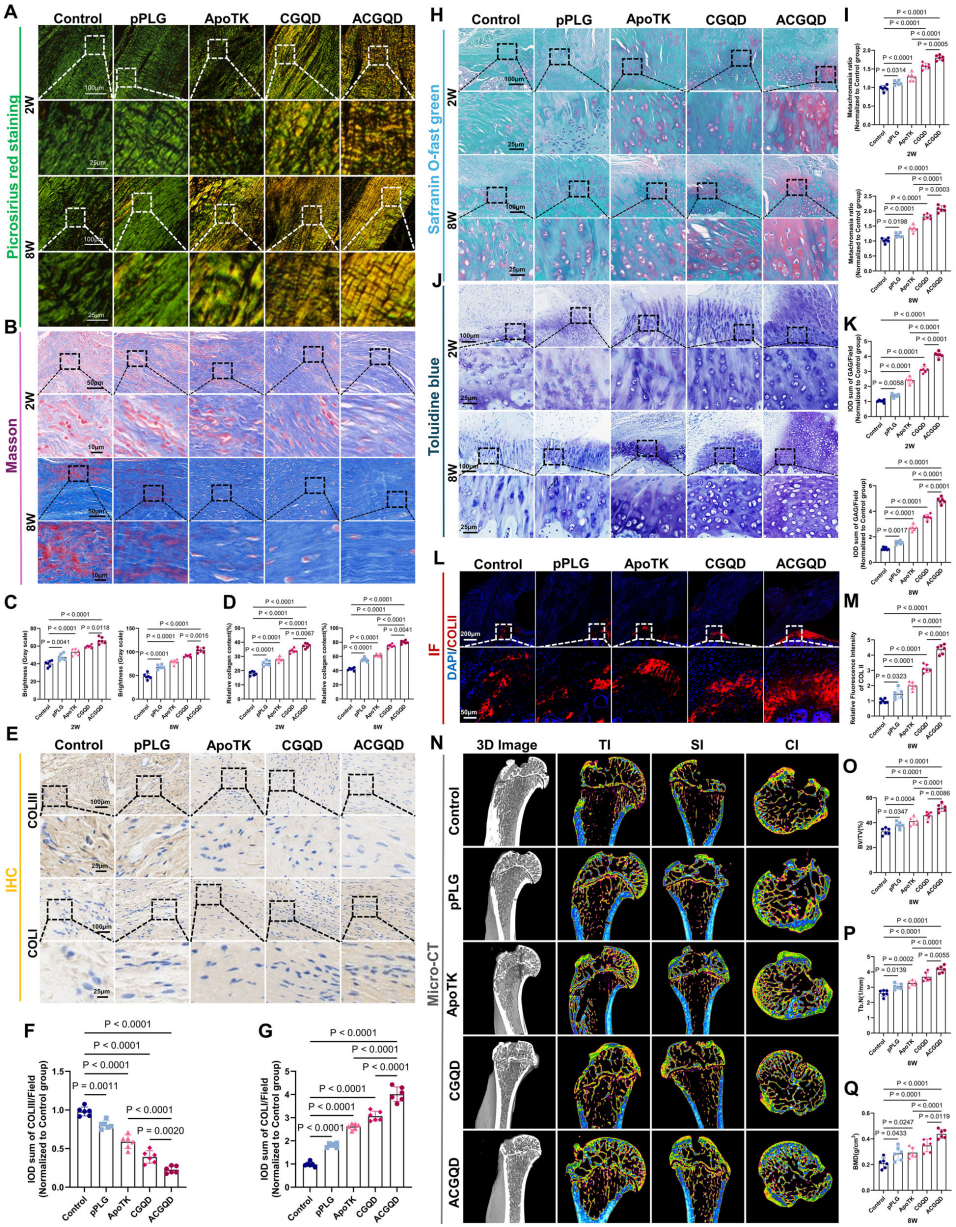
Tendon, interface matrix, and subchondral bone outcomes after rotator cuff repair in rats. The five repair groups are Control (suture repair only), unloaded pPLG, ApoTK@pPLG, CGQD@pPLG, and ACGQD@pPLG; the last three are abbreviated as ApoTK, CGQD, and ACGQD in the panels. (A) Picrosirius red staining under polarized light at 2 and 8 weeks; scale bars, 100 and 25 µm. (B) Masson trichrome staining at 2 and 8 weeks; scale bars, 50 and 10 µm. (C) Grayscale brightness of polarized picrosirius‐red images at 2 weeks (left) and 8 weeks (right), used as a birefringence readout. (D) Masson‐based relative collagen content at 2 weeks (left) and 8 weeks (right). (E) COL III (upper rows) and COL I (lower rows) immunohistochemistry at 8 weeks; scale bars, 100 µm and 25 µm. (F, G) COL III (F) and COL I (G) integrated optical density, expressed relative to Control. (H) Safranin O–fast green staining at 2 and 8 weeks. (I) Metachromasia ratio, defined here as Safranin O staining normalized to Control at each time point. (J) Toluidine blue staining at 2 and 8 weeks. (K) Integrated optical density of toluidine blue‐stained glycosaminoglycans, normalized to Control at each time point. Scale bars in H and J, 100 and 25 µm. (L) COL II immunofluorescence at 8 weeks; COL II and DAPI are shown in red and blue, respectively. Scale bars, 200 and 50 µm. (M) Relative COL II fluorescence intensity. (N) 3D micro‐computed tomography reconstructions of the humeral footprint and orthogonal trabecular views at 8 weeks. (O–Q) Bone volume fraction (BV/TV; O), trabecular number (Tb. N; P), and bone mineral density (BMD; Q). Dashed boxes indicate enlarged regions. Data are mean ± SD; *n* = 6 independent animals per group at each assessed time point. Comparisons were made by one‐way ANOVA with Tukey's post hoc test; *p* values are shown in the panels.

**FIGURE 9:**
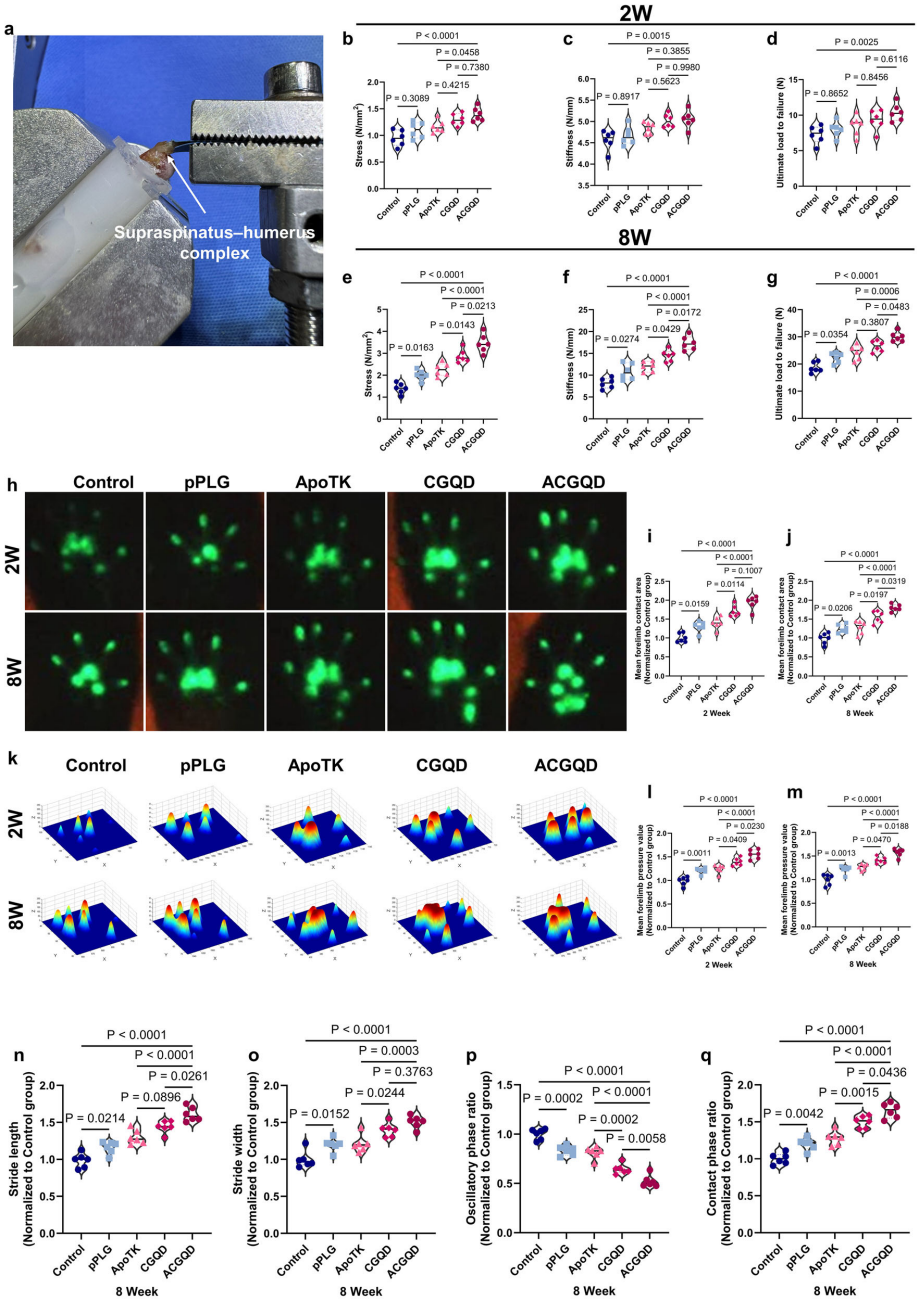
ACGQD@pPLG improves tendon–bone mechanical properties and gait measures. (a) Photograph of the tensile‐testing setup of the supraspinatus tendon–humerus complex. (b–d) Ultimate stress (b), stiffness (c), and ultimate load to failure (d) at 2 weeks. (e–g) Ultimate stress (e), stiffness (f), and ultimate load to failure (g) at 8 weeks. (h) Representative paw‐contact images. (i, j) Mean forelimb contact area at 2 weeks (i) and 8 weeks (j). (k) 3D walkway pressure‐signal maps. (l, m) Mean forelimb pressure at 2 weeks (l) and 8 weeks (m). (n–q) Stride length (n), stride width (o), oscillatory phase ratio (p), and contact phase ratio (q) at 8 weeks. Relative gait values use the mean of the repair‐only Control group at the corresponding time point as the denominator. The 8‐week unoperated reference is shown separately in Figure S43. Data are mean ± SD; *n* = 6 biologically independent animals per group. Comparisons were made by one‐way ANOVA with Tukey's post hoc test; *p* values are shown in the panels.

ACGQD@pPLG simultaneously suppressed M1‐like markers and increased M2‐like markers. Relative to Control, ACGQD reduced *TNF* and *NOS2* mRNA by approximately 68.00% and 77.23%, respectively. The CD68^+^iNOS^+^ fraction decreased from 65.86% ± 2.10% to 25.89% ± 2.35% (an approximately 60.69% reduction), and iNOS and CD86 fluorescence intensities decreased by approximately 75.25% and 63.73%, respectively (Figure 3B–F,H,J,L). ACGQD increased *ARG1* mRNA approximately 12.62‐fold and also increased *IL10* mRNA (Figure 3B). The CD68^+^CD206^+^ fraction rose approximately 9.97‐fold, from 3.98% ± 0.69% to 39.69% ± 1.44%. CD206 (immunofluorescence) and CD163 (Western blot) increased approximately 4.64‐ and 4.31‐fold, respectively, and arginase‐1 (Arg‐1) increased approximately 6.83‐fold by immunofluorescence and 6.91‐fold by Western blot (Figure 3B–E,G–I,K,M). Across these markers, responses were generally greatest with ACGQD, followed by CGQD and ApoTK, whereas unloaded pPLG had little effect relative to Control.

Consistent with these marker changes, enzyme‐linked immunosorbent assay (ELISA) of culture supernatants showed lower TNF‐α and higher IL‐10 secretion in the ACGQD@pPLG group than in the LPS‐only Control (Figure S27), indicating that treatment also altered the macrophage secretory profile.

To examine the contribution of the carbon‐dot component to the antioxidant response, we compared patches loaded with glutathione (GSH), GQDs, or CGQDs in the same Transwell co‐culture. Relative to LPS alone, CGQD‐loaded patches reduced 2′,7′‐dichlorodihydrofluorescein diacetate (DCFH‐DA) and dihydroethidium (DHE) fluorescence by approximately 90.70% and 85.50%, respectively, and reduced the flow‐cytometric DCFH‐DA mean fluorescence intensity by 39.00% (Figure 3N–R). GQD‐loaded patches were nearly as effective, whereas GSH‐loaded patches were the least effective in all three readouts. The carbon‐dot formulations produced the largest reductions in oxidant‐associated fluorescence among the formulations tested.

### 2.4 NRF2 Signaling and ASS1‐Associated Macrophage Responses

To identify treatment‐associated transcriptional pathways, we performed bulk RNA sequencing on LPS‐stimulated macrophages cultured without (Control) or with ACGQD@pPLG (Treat); unstimulated (Blank) and LY294002‐treated (Inhibitor) groups were included in the subsequent validation experiments. Principal component analysis (PCA) separated Treat from Control (Figure S29), and 337 transcripts were upregulated and 841 downregulated in Treat relative to Control (Figure 4A). Selected differentially expressed genes, grouped into inflammation, metabolism, signaling, and repair categories, changed in opposite directions (Figure 4C and Figure S30A): inflammatory mediators (*NOS2*, *MMP9*, *CCL2*, *IL1B*, *IL6*, and *TNF*) were suppressed, whereas arginine‐metabolism and polyamine genes (*ODC1*, *ARG1*, *ASL*, and *ASS1*), antioxidant genes (*GCLC*, *HMOX1*, *NFE2L2*, and *NQO1*), and tissue‐repair genes (*TGFB1*, *IL10*, and *MRC1*) were induced. KEGG and Gene Ontology (GO) enrichment analyses identified phosphatidylinositol 3‐kinase (PI3K)–Akt signaling, TNF signaling, nuclear factor‐κB (NF‐κB) signaling, and arginine biosynthesis among the top pathways (Figure 4B,D and Figure S30B). Gene set enrichment analysis (GSEA) showed positive enrichment of the PI3K–Akt pathway (normalized enrichment score [NES] = 1.442) and negative enrichment of the NF‐κB pathway (NES = −1.533) (Figure 4E), as well as negative enrichment scores for hypoxia‐inducible factor 1 (HIF‐1) signaling and ferroptosis (Figure S31).

We then examined the involvement of PI3K‐related signaling using the inhibitor LY294002. Treatment increased the phosphorylation of PI3K, AKT, and glycogen synthase kinase‐3β (GSK3β) by approximately 1.44‐, 1.53‐, and 1.71‐fold relative to Control, respectively, and these increases were reduced by LY294002 (Figure 4F,G). LY294002 also attenuated the induction of *NQO1*, *GCLC*, *ASS1*, and *ASL* transcripts (Figure S32). Treatment increased NRF2, heme oxygenase‐1 (HO‐1), and Arg‐1 protein levels and decreased iNOS, and LY294002 diminished these responses (Figure 4H,I). LY294002 only partially reduced nuclear NRF2 accumulation, and confocal imaging confirmed HO‐1 induction by treatment (Figure 4J–M). Together, these results support the involvement of PI3K‐related signaling in the NRF2‐related antioxidant and macrophage marker responses.

Steady‐state metabolomic profiling also separated Treat from Control by PCA and identified 40 metabolites with higher and 69 with lower abundance after treatment (Figure 4N,O). Metabolites with increased abundance included citrulline, arginine, ornithine, argininosuccinate, putrescine, glutamine, proline, and hydroxyproline species (Figure 4P,Q), whereas lactate, GSSG, and prostaglandin E2 (PGE2) decreased. Pathway mapping and enrichment analyses highlighted arginine biosynthesis, arginine–proline metabolism, and related amino acid pathways (Figure 4R–T), and correlation and network analyses revealed relationships among these metabolites (Figure S35). Figure 4U summarizes how these changes relate to the macrophage marker response.

We next used small interfering RNA (siRNA) knockdown to test the contributions of ASS1 and NRF2 to the macrophage response. ASS1 silencing reduced ASS1 protein and attenuated the ACGQD‐induced increase in CD206, whereas iNOS remained below the LPS‐only level in the silenced groups (Figure S33). NRF2 silencing reduced whole‐cell and nuclear NRF2 and diminished the induction of HO‐1 and NAD(P)H quinone oxidoreductase 1 (NQO1) (Figure S34), consistent with the reported anti‐inflammatory role of these enzymes in human monocytes [29]. These perturbations support a contribution of ASS1 to CD206 induction and of NRF2 to antioxidant‐enzyme induction.

### 2.5 Computational Interaction Models and Cellular NRF2 Regulation

We used molecular docking and 100‐ns molecular dynamics (MD) simulations to explore possible interactions of selected ligand models with KEAP1 and iNOS (Figure 5A). The CGQD representation docked to the Kelch domain of KEAP1 with a docking score of −9.608 kcal mol^−1^, with predicted contacts involving Arg415, Ser555, Ser383, Tyr334, Tyr572, and Phe577 (Figure 5B and Figure S36A). The NRF2‐derived ligand model yielded a score of −9.033 kcal mol^−1^ and shared contacts at Tyr334 and Ser555 (Figure 5C and Figure S36B). Free citrulline and arginine docked to the human iNOS heme domain with scores of −6.005 and −5.938 kcal mol^−1^, respectively (Figure 5D,E and Figure S36C,D), and their modeled poses shared contacts with Trp372 and Tyr373.

In the CGQD–KEAP1 simulation, the backbone root‐mean‐square deviation (RMSD) plateaued near 0.15 nm after approximately 20 ns, and the root‐mean‐square fluctuation (RMSF) of binding‐site residues remained below 0.25 nm (Figure 5F,G); the radius of gyration and solvent‐accessible surface area were also stable over the trajectory. Per‐residue energy decomposition identified Tyr334, Arg415, and Ser555 as the main contributors in this model (Figure 5H). The free‐energy landscape, electrostatic surface, residue cross‐correlations, and principal component analysis further characterized the trajectory (Figure 5I–M); the first three principal components accounted for approximately 31.7% of the total variance. Snapshots and trajectory analyses of the citrulline–iNOS complex are provided in Figures S37–S40. The simulations yield candidate contact patterns for experimental testing.

We then examined KEAP1–NRF2 association in cells by immunofluorescence colocalization and co‐immunoprecipitation. CGQD treatment reduced KEAP1–NRF2 colocalization in LPS‐stimulated cells from a Pearson's *R* of 0.84 to 0.50, close to the baseline value of 0.53 in unstimulated cells, whereas co‐treatment with LY294002 increased it to 0.70 (Figure 5N). With comparable input levels of KEAP1 and NRF2, less NRF2 co‐immunoprecipitated with KEAP1 after CGQD treatment (Figure 5O). CGQD treatment therefore reduces KEAP1–NRF2 association in cells.

Taken together, CGQD exposure was associated with reduced KEAP1–NRF2 association and an NRF2‐related antioxidant response in macrophages. The molecular models provide candidate contact patterns for testing, while the free‐citrulline comparison and knockdown experiments support selected cellular contributions (Figures S28, S33, and S34).

### 2.6 Conditioned Medium From Treated Macrophages Supports Stromal‐Cell Responses

To examine how conditioned medium from treated macrophages affects tendon and bone marrow progenitor cells, we performed conditioned‐medium transfer experiments (Figure 6A). PMA‐differentiated THP‐1 macrophages were stimulated with LPS and incubated without a patch (Control) or with the indicated patches (pPLG, ApoTK, CGQD, or ACGQD) for 48 h, washed with PBS, and incubated in serum‐free RPMI 1640 for a further 24 h. The conditioned media were then collected and applied to TDSCs or BMSCs.

Conditioned medium from ACGQD‐treated macrophages enhanced TDSC migration and tenogenic marker expression. The number of transmigrated TDSCs in the ACGQD group reached 121.17 ± 4.49 cells per field at 24 h and 291.67 ± 11.17 at 48 h, compared with 40.67 ± 3.72 and 81.67 ± 6.62, respectively, in Control (approximately 2.98‐ and 3.57‐fold increases; Figure 6B,D). Scratch‐wound closure at 24 h was 63.62% ± 4.19% with ACGQD vs. 20.03% ± 2.42% with Control (approximately 3.18‐fold; Figure 6C,E). At day 7, ACGQD‐conditioned medium increased the immunofluorescence intensities of COL1 and scleraxis (SCX) in TDSCs approximately 2.09‐ and 2.37‐fold, respectively (Figure 6F–I), and quantitative PCR (qPCR) showed approximately 3.85‐, 3.01‐, and 4.47‐fold induction of *SCX*, tenomodulin (*TNMD*), and *COL1A1*, respectively (Figure 6U–W).

ACGQD‐conditioned medium also promoted the osteogenic differentiation of BMSCs. At day 7, immunofluorescence intensities of Runt‐related transcription factor 2 (RUNX2) and osteocalcin (OCN) were approximately 2.43‐ and 2.53‐fold higher than in Control (Figure 6J–M), and alkaline phosphatase (ALP) activity was approximately 3.23‐fold higher (Figure 6N,O). Alizarin Red S staining showed approximately 2.95‐fold greater mineralization at day 21 (Figure 6P,Q), and qPCR showed approximately 4.34‐, 10.54‐, and 5.91‐fold induction of *RUNX2*, osteopontin (*OPN*), and *COL1A1*, respectively (Figure 6R–T).

Across the migration and differentiation assays, ACGQD‐conditioned medium produced the strongest responses, ApoTK and CGQD produced intermediate responses, and unloaded pPLG had little effect (Figure 6). These conditioned‐medium responses support further evaluation of the patch in vivo.

### 2.7 ACGQD@pPLG Modulates the Early Repair Environment in Rats

We next evaluated the patches in a rat model of rotator cuff tear and repair. After reattachment of the supraspinatus tendon, the assigned patch was placed at the tendon‐to‐bone interface, and early tissue responses were assessed at day 14 (Figure 7A and Figure S41).

At day 14, ACGQD@pPLG‐treated tendons showed lower CD86 and TNF‐α fluorescence and higher CD206 and IL‐10 fluorescence than repair‐only Control (Figure 7B–D), consistent with the macrophage responses observed in vitro (Figure 3).

CD206/COL I co‐staining showed CD206‐positive cells within collagen‐rich repair tissue (Figure 7G), and both CD206 and COL I fluorescence were higher in the ACGQD@pPLG group than in repair‐only Control (Figure 7I). NRF2 and HO‐1 fluorescence was also higher in treated tissue (Figure 7H,I), in line with the antioxidant response observed in cultured macrophages.

CD90/CD44 co‐staining identified more double‐positive cells in the ACGQD@pPLG group than in repair‐only Control (Figure 7E,F). Hematoxylin and eosin (H&E) staining showed more organized repair tissue after treatment (Figure 7J), and the modified tendon healing score was 10.17 ± 0.75 in ACGQD@pPLG‐treated tendons vs. 3.33 ± 0.52 in Control (Figure 7K; scoring criteria in Table S1).

At day 14, ACGQD@pPLG shifted the balance of inflammatory and reparative markers, enhanced NRF2/HO‐1 and collagen staining, and increased the number of CD44^+^CD90^+^ cells. The unloaded pPLG scaffold also improved several of these measures relative to Control. Longer‐term matrix and bone outcomes were assessed at 8 weeks (Figure 8).

### 2.8 ACGQD@pPLG Improves Tendon, Interface Matrix, and Bone Outcomes

Longer‐term outcomes were evaluated in the same rotator cuff repair model. The tendon was assessed by picrosirius red and Masson trichrome staining at 2 and 8 weeks and by COL I/COL III immunohistochemistry at 8 weeks. The fibrocartilaginous tendon‐to‐bone interface was assessed by Safranin O‐fast green and toluidine blue staining at 2 and 8 weeks and by COL II immunofluorescence at 8 weeks, and subchondral bone microarchitecture was evaluated by micro‐computed tomography (micro‐CT) at 8 weeks (day 56).

Under polarized light, picrosirius red staining showed more organized collagen bundles in ACGQD@pPLG‐treated tissue (Figure 8A). Grayscale brightness, a readout of collagen birefringence and organization [30], was higher than in repair‐only Control at both 2 and 8 weeks (Figure 8C). At 8 weeks, Masson‐based relative collagen content was 80.03% ± 2.16% in ACGQD@pPLG‐treated tissue vs. 42.17% ± 1.90% in Control (Figure 8B,D). Collagen type was assessed separately by immunohistochemistry, which showed stronger COL I and weaker COL III staining after treatment (Figure 8E–G).

At the tendon‐to‐bone insertion, ACGQD@pPLG treatment increased Safranin O–fast green and toluidine blue staining, which reflect proteoglycan and glycosaminoglycan content, as well as COL II fluorescence, a marker of fibrocartilaginous matrix, relative to repair‐only Control (Figure 8H–M).

Micro‐CT analysis of the underlying subchondral bone (Figure 8N–Q) showed denser and more organized trabecular networks beneath the humeral footprint in ACGQD‐treated animals. Bone volume fraction (BV/TV) and trabecular number (Tb.N) were approximately 1.57 and 1.60 times the Control values, respectively. Bone mineral density (BMD) reached 0.44 ± 0.03 g cm^−3^, compared with 0.21 ± 0.04 g cm^−3^ in Control (approximately 2.08 times the Control value).

Overall, at 8 weeks ACGQD@pPLG improved collagen organization and content, proteoglycan and COL II staining at the interface, and subchondral bone parameters relative to suture repair alone. Comparison with the unoperated Sham reference showed residual histological differences (Figure S42), indicating incomplete restoration of native tissue characteristics.

### 2.9 ACGQD@pPLG Improves Tendon–Bone Mechanical Properties and Gait

Biomechanical testing of the repaired supraspinatus tendon–humerus complex under monotonic loading to failure (Figure 9a–g) showed higher ultimate stress, stiffness, and ultimate load to failure in the ACGQD@pPLG group than in repair‐only Control, with larger differences at 8 weeks. At this time point, ultimate stress was 3.45 ± 0.42 vs. 1.40 ± 0.23 N mm^−2^ (Figure 9e), stiffness was 17.40 ± 1.52 vs. 8.17 ± 1.18 N mm^−1^ (Figure 9f), and ultimate load to failure was 30.33 ± 1.69 vs. 18.67 ± 1.88 N (Figure 9g).

Gait was assessed at 2‐ and 8‐week using paw‐contact imaging and a pressure‐sensitive walkway (Figure 9h–m); all values were normalized to the mean of the repair‐only Control group at the corresponding time point. Forelimb contact pressure and contact area were higher in ACGQD@pPLG‐treated animals than in Control, and at 8 weeks, stride length, stride width, and phase ratios also differed from Control (Figure 9n–q). In the 8‐week Sham comparison, the contact pressure, contact area, stride width, and stride length of treated animals were closer to the unoperated reference, but residual differences remained (Figure S43). These findings support improved use of the operated limb over the assessment period.

### 2.10 Local Implant Tolerability and Rabbit Repair Outcomes

After local implantation of a patch loaded with fluorescently labeled nanoparticles, fluorescence at the implantation site remained detectable at days 0, 3, 7, and 10 and gradually declined (Figure S44). At 8 weeks, H&E‐stained sections of major organs showed no overt treatment‐related lesions (Figure S45), and hematological and serum biochemical parameters did not differ significantly between groups (Figure S46). No overt systemic toxicity was detected by these 8‐week endpoints.

Finally, we evaluated the patch in a rabbit rotator cuff repair model, which provides a larger anatomical scale (Figure S47A). At 10 weeks, ACGQD@pPLG‐treated tissue showed more organized collagen and stronger Safranin O staining at the interface than repair‐only Control (Figure S47B), with a relative collagen content of 86.00% ± 3.20% vs. 53.50% ± 4.00% and a metachromasia ratio approximately 2.38 times that of Control (Figure S47C). These results extend the histological findings beyond the rat model.

## 3 Discussion

Macrophage redox regulation and arginine‐associated metabolism provide complementary targets for biological augmentation of rotator cuff repair. Surgical reattachment remains the standard treatment, yet retears are common, particularly after repair of large or massive tears [8, 9, 10]. In our exploratory single‐cell analysis, M1‐like cells constituted a higher proportion of recovered macrophages in injured supraspinatus specimens than in non‐injured semitendinosus specimens. *ASS1* and *ASL* transcripts were sparsely detected, while tissue staining showed higher iNOS and 4‐HNE fluorescence in injured specimens. Each group comprised three donors, and the anatomically distinct tendons differ in their mechanical environments, limiting attribution of these observations to rotator cuff injury alone. Metabolomic profiling of polarized THP‐1 macrophages identified glutathione redox imbalance and reduced arginine and proline abundance in M1 cells, with spermidine enriched in M2 cells. These metabolic findings extend the rationale for macrophage modulation beyond the cellular differences observed in human tendon specimens. ASS1 regulation is context‐dependent, as infection and IFN‐γ/LPS stimulation can induce citrulline utilization to sustain proinflammatory macrophage activity [31, 32]. By contrast, ASS1 inhibition can alleviate inflammation in selected immune‐mediated disorders [33]. The contribution of citrulline metabolism therefore needs to be evaluated within the inflammatory setting of tendon injury. The arginine, proline, and spermidine signature identified here complements the cellular changes described in recent tendon single‐cell studies [12, 13]. It also informed our use of citrulline functionalization for local macrophage modulation, consistent with reported effects of citrulline on macrophage metabolism and inflammation [34].

To examine how the patch modulates these responses, we assessed NRF2 signaling alongside ASS1/ASL expression. ACGQD@pPLG increased NRF2‐related antioxidant transcription (*NQO1*, *GCLC*) and *ASS1*/*ASL* expression. LY294002 attenuated induction of all four target genes by approximately 79%–82% and partially reduced nuclear NRF2 accumulation. NRF2 knockdown diminished HO‐1 and NQO1 induction, whereas ASS1 knockdown attenuated CD206 induction. These findings support PI3K‐related involvement in the measured responses, NRF2‐dependent antioxidant‐enzyme induction, and a contribution of ASS1 to CD206 induction. A related immunometabolic strategy was reported by Liang et al. [35], who used NAD^+^‐loaded membrane nanocarriers to modulate inflammatory macrophages and improve bone and skin regeneration in aged models. Although the metabolic targets and injury settings differ, both approaches use local delivery to influence macrophage metabolism during tissue repair.

The NRF2 response was accompanied by reduced KEAP1–NRF2 association after CGQD treatment. KEAP1–NRF2 colocalization decreased from a Pearson's R of 0.84 in LPS‐stimulated macrophages to 0.50 after treatment. Co‐immunoprecipitation also showed reduced NRF2 pull‐down, with comparable KEAP1 and NRF2 input levels. These cellular findings are consistent with reports that carbon dots can induce NRF2‐related antioxidant responses or act as broad‐spectrum antioxidants [36, 37]. Docking and 100‐ns molecular dynamics (MD) simulations provided a candidate structural explanation using a representative CGQD model at the KEAP1 Kelch β‐propeller interface. The CGQD docking score was −9.608 kcal mol^−^^1^, compared with −9.033 kcal mol^−^^1^ for the NRF2‐derived ligand model, with Tyr334 and Ser555 shared as contact residues. A single deep free‐energy basin was consistent with a stable pose within the simulated system. Citrulline docking in the iNOS substrate pocket yielded a score of −6.005 kcal mol^−^^1^, compared with −5.938 kcal mol^−^^1^ for arginine. This comparison suggests a possible substrate‐pocket interaction for experimental testing. The contribution of citrulline functionalization was examined separately by comparing macrophage‐marker responses. At the same nominal mass concentration (50 µg mL^−^^1^), CGQDs produced larger changes in iNOS and CD206 than free L‐citrulline or GQDs (Figure S28). This result supports the functionalized formulation for modulating these markers under the tested dosing conditions. Conditioned medium from treated macrophages also enhanced TDSC migration and tenogenic marker expression, as well as osteogenic responses in stromal cells (Figure 6). These responses connect macrophage modulation to stromal processes relevant to tendon‐to‐bone repair.

The scaffold design combines macrophage modulation with local delivery through aligned fibers. The coating prepared from UV‐treated TDSCs showed higher calreticulin and lower CD47 than the untreated‐cell membrane control. ACGQD also showed greater macrophage association than CGQD@NM, consistent with prior apoptotic‐membrane delivery platforms [27, 38]. Incorporating thioketal linkers added an oxidant‐responsive delivery component. The formulation showed greater cumulative cargo release in H_2_O_2_‐containing medium than in PBS at 28 days, consistent with thioketal‐responsive delivery reported in osteoarthritis [39]. The aligned PLLA‐shell/GelMA‐core architecture was designed to accommodate early local delivery alongside longer‐term matrix remodeling. Slower PLLA degradation and cargo release from PLG over weeks provide a material basis for these different timescales. Growth‐factor and ionic‐gradient scaffolds have also been developed for tendon‐to‐bone repair [16, 24]. The present design combines aligned topography with membrane‐assisted cell association and redox–metabolic modulation to address the structural and inflammatory aspects of repair.

Extending this local delivery approach to rotator cuff repair requires consideration of both formulation reproducibility and the biological settings examined here. The membrane preparation was derived from unsorted UV‐treated TDSCs, making batch‐to‐batch consistency an important consideration for further development. The human tendon comparison involved three donors per group, anatomically distinct tendon origins, and no stratification by tear size or fatty infiltration grade. Larger comparisons within the same tendon type would help distinguish injury‐associated changes from anatomical variation. Experiments in primary macrophages would also establish how well the responses in PMA‐differentiated THP‐1 cells extend to other macrophage populations. At the molecular level, direct binding assays could test the proposed interaction models, while stable‐isotope tracing combined with ASS1/ASL perturbation and rescue could resolve citrulline‐to‐arginine conversion. Selective PI3K perturbation would clarify the pathway contributions currently assessed pharmacologically [40]. The rat functional and biomechanical findings and rabbit histology provide a basis for evaluating repair durability in chronic large‐animal models, including established ovine models [41]. Such studies should assess structural repair and function together over longer follow‐up. Clinical evidence that platelet‐rich plasma augmentation can reduce retear rates without improving patient‐reported function supports the combined assessment of these endpoints [11]. Longer‐term follow‐up would also extend the assessment of local tissue responses and tolerability.

In conclusion, these findings support an immunometabolic approach to rotator cuff repair in which macrophage redox regulation and arginine‐associated metabolism complement structural reattachment. Observations from human tendon specimens and metabolomic profiling of polarized macrophages informed the design of ACGQD@pPLG. The patch increased NRF2‐related antioxidant responses and *ASS1*/*ASL* expression, while perturbation experiments supported PI3K‐related involvement and a contribution of ASS1 to CD206 induction. Cellular KEAP1–NRF2 association measurements support NRF2 regulation, while computational models provide candidate interaction modes. Improvements in rat biomechanics and gait, together with rabbit enthesis histology and short‐term tolerability findings, support further evaluation of this local scaffold for tendon‐to‐bone repair.

## 4 Materials and Methods

### 4.1 Ethics

All experimental procedures involving human tissues were conducted in accordance with the Declaration of Helsinki and were approved by the Institutional Review Board (IRB) of Shanghai Sixth People's Hospital Affiliated to Shanghai Jiao Tong University School of Medicine (No. 2023‐KY‐074(K)). Written informed consent was obtained from all participants. All animal experiments were performed in accordance with protocols approved by the Institutional Animal Care and Use Committee (IACUC) of Shanghai Sixth People's Hospital (No. DWLL2026‐0026) and followed the *Guide for the Care and Use of Laboratory Animals*.

### 4.2 Human Samples and Animals

#### 4.2.1 Human Tendon Specimens

Supraspinatus tear‐stump tissue (∼0.5 cm^3^) was obtained arthroscopically from three patients undergoing rotator cuff repair. Semitendinosus tendons from three different patients undergoing anterior cruciate ligament reconstruction served as non‐injured comparison tissues. The two specimen groups came from different patients and anatomically distinct tendon sites. Specimens were rinsed in cold PBS, divided, and processed for single‐cell dissociation within 1 h or for histology.

#### 4.2.2 Animal Housing and Allocation

Male Sprague–Dawley rats and male New Zealand white rabbits were housed under specific‐pathogen‐free conditions at 22 ± 2°C and 50% ± 10% humidity, with a 12 h light/dark cycle and ad libitum access to food and water. Treatment groups and assessment time points are described in Section 4.12.

### 4.3 Materials, Reagents, Antibodies, and Cells

L‐citrulline, L‐glutathione, poly(L‐lactic acid) (PLLA, MW ∼150 kDa), GelMA (∼80% degree of substitution), EDC, NHS, hexafluoroisopropanol (HFIP), and lithium phenyl‐2,4,6‐trimethylbenzoylphosphinate (LAP) were purchased from Aladdin or Macklin (Shanghai, China). DSPE–PEG–TK was custom‐synthesized by Ruixibio (Xi'an, China). DCFH‐DA, DHE, Calcein‐AM/PI, Hoechst 33342, LPS, PMA, and IL‐4 were obtained from Beyotime or Sigma‐Aldrich. Antibodies are listed in Table S2. TDSCs (cat. SCRS‐C108), BMSCs (cat. BMRS‐C106), and THP‐1 cells (cat. TCH‐C361) were purchased from Suzhou Haixing Biotechnology.

### 4.4 Synthesis of CGQD and ACGQD Nanoparticles

#### 4.4.1 Carbon‐Dot Synthesis and Citrulline Functionalization

GQDs were prepared from citric acid and L‐glutathione by hydrothermal synthesis at 120°C for 12 h, then filtered through a 0.22 µm membrane, dialyzed (molecular weight cut‐off [MWCO], 1 kDa; 48 h), and lyophilized. For EDC/NHS‐mediated amide coupling, GQDs (10 mg) in 2‐(*N*‐morpholino)ethanesulfonic acid (MES) buffer (50 mM, pH 5.5) were activated with EDC (40 mg) and NHS (24 mg) for 30 min, then reacted with L‐citrulline (50 mg) at room temperature for 24 h. The resulting CGQDs were dialyzed (MWCO, 1 kDa; 72 h) and lyophilized. DSPE–PEG–TK was characterized by ^1^H NMR spectroscopy on a Bruker AVANCE 600 MHz instrument (Figure S16).

#### 4.4.2 Membrane Preparation and Nanoparticle Assembly

TDSCs were UV‐irradiated (254 nm, 100 mJ cm^−2^) and incubated for 12 h. Apoptosis was assessed by Annexin V‐FITC/PI flow cytometry, and the UV‐treated population was collected without apoptotic‐cell enrichment. Cells were washed, resuspended in hypotonic buffer (10 mm Tris‐HCl, pH 7.5, 10 mm NaCl, 1 mm EDTA, and protease inhibitor cocktail), and disrupted by three freeze‐thaw cycles and sonication (50 W, 5 min, on ice). Membranes were collected by differential centrifugation (700 × g for 10 min, followed by 14 000 × g for 30 min) and resuspended in PBS at 2 mg mL^−1^. DSPE–PEG–TK (1 mg) was dried into a thin film, rehydrated with the membrane suspension, and extruded through a 200 nm polycarbonate membrane for 11 cycles to obtain ApoTK. CGQDs (1 mg) were added, incubated at 37°C for 1 h, and re‐extruded to form ACGQD. Free CGQDs were removed by ultracentrifugation (100 000 × g, 1 h, 4°C).

### 4.5 Fabrication and Characterization of the ACGQD@pPLG Patch

#### 4.5.1 Coaxial Electrospinning

Aligned pPLG fibers were prepared by coaxial electrospinning. The shell solution contained 12% (w/v) PLLA in HFIP, and the core solution contained 10% (w/v) GelMA and 0.5% (w/v) LAP in deionized water. For ACGQD@pPLG, ACGQD was added to the core solution at 0.5 mg mL^−^^1^. Electrospinning was performed at 18 kV with shell and core flow rates of 0.5 and 0.2 mL h^−^^1^, respectively, a collection distance of 15 cm, and a rotating drum speed of 2000 rpm. The mats were UV‐crosslinked (365 nm, 5 min), vacuum‐dried for 48 h, and stored at −20°C.

#### 4.5.2 Physicochemical Characterization

Materials were characterized by TEM (Hitachi HT7700, 100 kV), DLS and zeta potential measurements (Malvern Zetasizer Nano ZS), FTIR spectroscopy (Thermo Nicolet iS50), XPS (Thermo K‐Alpha, Al Kα), SEM (Zeiss Sigma 300), AFM, and contact‐angle measurements (DataPhysics OCA 25). Antioxidant activity was examined by EPR spectroscopy and by ABTS•^+^, DPPH•, O_2_•^−^, and •OH scavenging assays over a range of CGQD concentrations.

#### 4.5.3 Patch Tensile Testing

Patch specimens were tested in tension to obtain stress–strain data. Tensile strength was calculated as the maximum load divided by the initial cross‐sectional area, Young's modulus as the slope of the initial linear region of the stress–strain curve, and elongation at break as the extension at failure relative to the initial gauge length. Elongation at break was also assessed after incubation in PBS at 37°C.

#### 4.5.4 Cargo Release and Scaffold Degradation

CGQD cargo release from ACGQD was evaluated in PBS, 100 µm H_2_O_2_, or 1 mm H_2_O_2_ at 37°C. Carbon‐dot‐associated fluorescence was quantified at excitation and emission wavelengths of 360 and 460 nm, respectively (Figure S22). Cumulative release of ACGQD‐associated cargo from PLG and PLLA mats was measured in PBS at 37°C over 28 days using the same fluorescence readout. For degradation measurements, PLG and PLLA mats were incubated in PBS at 37°C for up to 120 days and weighed after lyophilization.

### 4.6 THP‐1 Macrophage Culture, Polarization, and Treatment Groups

#### 4.6.1 Macrophage Differentiation and Polarization

THP‐1 monocytes were maintained in RPMI 1640 medium supplemented with 10% fetal bovine serum (FBS), 1% penicillin‐streptomycin, and 50 µm β‐mercaptoethanol. Cells were differentiated into macrophages with 100 ng mL^−^^1^ PMA for 48 h, followed by a 24 h resting period. For reference‐state profiling, macrophages were left unpolarized (M0) or polarized to M1 and M2 states. Polarization was assessed by flow cytometry (CD68/iNOS and CD68/CD206) and qPCR (*TNF*, *NOS2*, *ARG1*, and *IL10*).

#### 4.6.2 Patch Co‐Culture and Treatment Groups

Patch effects were evaluated in five LPS‐stimulated groups: Control (LPS without a patch), pPLG, ApoTK@pPLG, CGQD@pPLG, and ACGQD@pPLG. Unstimulated cells (Blank) served as an additional reference where indicated. Patches were placed in the upper compartment of 0.4 µm Transwell inserts (Corning), with cells seeded in the lower compartment. This arrangement separated the patch from the cells while permitting exchange of soluble factors and released material components. In the macrophage‐marker experiments, transcripts were analyzed at 12 h and protein markers at 24 h. Assay‐specific sample sizes are reported in the corresponding figure legends.

#### 4.6.3 PI3K‐Inhibitor Experiments

Four groups were compared: Blank, Control, Treat (LPS + ACGQD@pPLG), and Inhibitor (LPS + ACGQD@pPLG + 10 µM LY294002). LY294002 was added 1 h before treatment.

### 4.7 Molecular and Cellular Assays

#### 4.7.1 Quantitative PCR

Total RNA was extracted with TRIzol (Invitrogen), and cDNA was synthesized from 1 µg of RNA using the PrimeScript RT Reagent Kit (Takara). qPCR was performed with TB Green Premix Ex Taq II on a QuantStudio 7 Flex system using 95°C for 30 s, followed by 40 cycles of 95°C for 5 s and 60°C for 30 s. Relative expression was calculated using the 2^−ΔΔCt^ method, with *GAPDH* as the reference gene. Primers are listed in Table S3.

#### 4.7.2 Flow Cytometry and Oxidant‐Sensitive Probe Assays

Macrophages were detached with Accumax (Innovative Cell Technologies), blocked with TruStain FcX (BioLegend), and stained with antibodies against CD68/iNOS or CD68/CD206. Oxidant‐sensitive probe fluorescence was measured after incubation with 10 µm DCFH‐DA or 5 µm DHE for 30 min at 37°C. Apoptosis was assessed with an Annexin V‐FITC/PI kit (Beyotime). Samples were analyzed on a BD FACSAria III flow cytometer, and data were processed in FlowJo v10.

#### 4.7.3 Immunofluorescence

Samples were fixed in 4% paraformaldehyde for 15 min, permeabilized with 0.1% Triton X‐100 for 10 min, blocked with 5% goat serum for 1 h, and incubated with primary antibodies (1:200, overnight at 4°C) and Alexa Fluor‐conjugated secondary antibodies (1:500, 1 h). Nuclei were counterstained with DAPI, and images were acquired on a Leica SP8 confocal microscope. KEAP1–NRF2 colocalization was assessed from the fluorescence images using Pearson's correlation coefficient (Figure 5N).

#### 4.7.4 Immunoblotting and Immunoprecipitation

Protein extracted with RIPA buffer (20 µg per lane) was separated by SDS‐PAGE, transferred to PVDF membranes, blocked with 5% non‐fat milk, and probed with primary (1:1000) and HRP‐conjugated secondary (1:5000) antibodies. Signals were detected by enhanced chemiluminescence on a Tanon imaging system and quantified in ImageJ. Nuclear and cytoplasmic NRF2 were examined in separated protein fractions, with Lamin B1 and β‐actin as the respective loading controls (Figure 4J,K). KEAP1–NRF2 association was examined by immunoprecipitation with anti‐KEAP1 followed by NRF2 immunoblotting, alongside input and IgG controls (Figure 5O).

#### 4.7.5 Cell Viability and Metabolic Activity

Cell viability in THP‐1‐derived macrophages and BMSCs was assessed by Calcein‐AM/PI staining (2 µM/4 µM, 30 min, 37°C). Metabolic activity was measured using the CCK‐8 assay (Beyotime) on days 1, 3, and 5 in TDSCs, BMSCs, and THP‐1‐derived macrophages.

#### 4.7.6 Cytokine ELISA

TNF‐α and IL‐10 concentrations were measured in macrophage culture supernatants from the Blank, Control, pPLG, ApoTK@pPLG, CGQD@pPLG, and ACGQD@pPLG groups using commercial ELISA kits (Servicebio, Wuhan, China) according to the manufacturer's instructions (Figure S27).

#### 4.7.7 Component Comparisons

For the redox comparison, LPS‐stimulated macrophages were co‐cultured in the Transwell system with patches loaded with GSH, GQDs, or CGQDs, using LPS alone as the reference. DCFH‐DA and DHE fluorescence were measured by imaging, and DCFH‐DA fluorescence was also quantified by flow cytometry, as described above (Figure 3N–R).

For the free‐citrulline comparison, LPS‐stimulated macrophages were exposed to free L‐citrulline (50 µg mL^−^^1^), GQDs (50 µg mL^−^^1^), or CGQDs (50 µg mL^−^^1^) for 24 h, with LPS alone as the reference. The same nominal total mass concentration of each test material was used to compare iNOS and CD206 responses at a common mass exposure; doses were not matched for citrulline content or molarity. iNOS and CD206 were assessed by immunoblotting and normalized to β‐actin (Figure S28).

#### 4.7.8 ASS1 And NRF2 Silencing

ASS1 and NRF2 were transiently silenced in THP‐1‐derived macrophages using target‐specific small interfering RNAs, with a non‐targeting negative control (si‐NC). After transfection and recovery, cells received the assigned treatments. Knockdown was verified by immunoblotting.

Each silencing experiment compared LPS, LPS + ACGQD, LPS + target‐specific siRNA, and LPS + ACGQD + target‐specific siRNA, with a non‐targeting‐siRNA control. In the ASS1 experiment, ASS1, iNOS, and CD206 were measured, with GAPDH as the loading control (Figure S33). In the NRF2 experiment, whole‐cell NRF2, nuclear NRF2, HO‐1, and NQO1 were measured; whole‐cell proteins were normalized to β‐actin and nuclear NRF2 to Lamin B1 (Figure S34).

#### 4.7.9 Particle–Cell Association

Total macrophage‐associated fluorescence after exposure to ACGQD or CGQD@NM was compared by confocal imaging (Figure S19). For flow cytometry, the membrane coating was labeled with DiD, and cell‐associated fluorescence was measured at 0, 1, 2, 8, and 12 h (Figure S20).

### 4.8 Single‐Cell RNA Sequencing

#### 4.8.1 Cell Isolation and Sequencing

Tendon specimens were minced and digested with 0.2% collagenase II (Worthington) and 2.5 U mL^−^^1^ Dispase II (Gibco) at 37°C for 4 h with gentle agitation. The suspension was passed through a 70 µm strainer, treated with ACK lysis buffer, and resuspended in PBS containing 0.04% bovine serum albumin. Cell viability exceeded 90% by trypan blue exclusion. Cells were loaded onto a 10x Genomics Chromium controller using the Chromium Next GEM Single Cell 3′ Reagent Kit v3.1, and libraries were sequenced on an Illumina NovaSeq 6000 platform (150 bp paired‐end). Reads were aligned to the GRCh38 reference genome with Cell Ranger v6.1.2.

#### 4.8.2 Single‐Cell Data Analysis

Quality control, normalization, dimensionality reduction, clustering, uniform manifold approximation and projection (UMAP), and differential expression analysis were performed in Seurat v4 [42]. Cell–cell communication was inferred with CellChat v1.6 [43]. Donor‐resolved macrophage embeddings and state proportions are presented in Figures S2 and S3, and *sparsely detected ASS1*/*ASL* transcripts are displayed in Figures S4 and S5. Donors were the biological units for interpreting the clinical comparison [44].

### 4.9 Bulk RNA Sequencing and Metabolomics

#### 4.9.1 Bulk RNA Sequencing

Bulk RNA sequencing compared LPS‐stimulated macrophages cultured without a patch (Control) or with ACGQD@pPLG (Treat). Libraries were constructed from total RNA (RNA integrity number > 8) using the TruSeq Stranded mRNA Library Prep Kit (Illumina) and sequenced on a NovaSeq 6000 platform (2 × 150 bp; approximately 30 million reads per sample). Reads were aligned to GRCh38 with HISAT2 and counted with featureCounts. Differential expression was analyzed with DESeq2 using |log_2_FC| > 1 and a false discovery rate < 0.05. GSEA was performed with Hallmark and KEGG gene sets, and enrichment analyses were performed with clusterProfiler.

#### 4.9.2 Widely Targeted Metabolomics

Macrophages from the M0, M1, and M2 reference states or from the Control and Treat conditions were analyzed by widely targeted metabolomics. Cells were quenched in 80% methanol at −80°C, sonicated, centrifuged (12 000 × g, 10 min, 4°C), and filtered through a 0.22 µm membrane. Extracts were analyzed by ultra‐performance liquid chromatography–tandem mass spectrometry (UPLC‐MS/MS) on a Shimadzu LC‐30A system coupled to an AB Sciex QTRAP 6500, using a database of more than 900 compounds (Metware Biotechnology). Multivariate analysis, hierarchical clustering, and KEGG analysis were used to examine sample separation, metabolite patterns, and pathway associations. Standardized metabolite‐abundance profiles across M0, M1, and M2 states were clustered with Mfuzz (Figure S9) [45]. Differential metabolites were defined using VIP > 1, |log_2_FC| > 0.5, and *p* < 0.05. Pearson correlations were used to explore metabolite–metabolite and gene–metabolite associations (Figure S35).

### 4.10 Molecular Docking and Dynamics Simulation

#### 4.10.1 Receptor Preparation and Docking

Receptor structures were obtained from the Protein Data Bank: the human KEAP1 Kelch domain–NRF2 peptide complex (PDB 2FLU) [46] and the human iNOS heme domain in complex with L‐arginine (PDB 1NSI) [47]. Structures were prepared in AutoDock Tools by adding polar hydrogens and assigning Gasteiger charges [48]. Docking was performed with AutoDock Vina [49], using search boxes centered on the NRF2‐binding interface of KEAP1 and the L‐arginine‐binding site of iNOS. Poses were selected according to docking score and conformational plausibility.

#### 4.10.2 Ligand Models

A representative CGQD ligand model was constructed to describe the surface‐functionalized carbon‐dot preparation, informed by the XPS and FTIR characterization in Figure 2B–D. Its geometry was optimized before docking. The model was a simplified representative structure for the chemically heterogeneous CGQD preparation. An NRF2‐derived ligand model was used for the KEAP1 comparison, and free L‐citrulline and L‐arginine were modeled separately as ligands for iNOS.

#### 4.10.3 Molecular Dynamics and Trajectory Analysis

Molecular dynamics simulations of the docked complexes were performed using GROMACS [50]. Receptor topologies were prepared with pdb2gmx using the AMBER ff14SB force field [51]. For free citrulline, the geometry was optimized in Gaussian, atomic charges were calculated with Multiwfn [52], and the ligand topology was constructed with Sobtop using GAFF parameters [53]. Complexes were solvated in TIP3P water under periodic boundary conditions [54], neutralized with counterions, energy‐minimized, and equilibrated before 100‐ns production simulations. Covalent bond lengths were constrained with LINCS, and long‐range electrostatic interactions were treated using Particle Mesh Ewald (PME).

Trajectories were analyzed for root‐mean‐square deviation (RMSD), root‐mean‐square fluctuation (RMSF), radius of gyration (Rg), solvent‐accessible surface area, and intermolecular contacts, including hydrogen bonds. Electrostatic surfaces, residue cross‐correlations, and principal component analysis were also examined. Free‐energy landscapes were projected onto RMSD–Rg, principal‐component, or time‐lagged independent‐component coordinates as shown in Figure 5 and Figures S39 and S40; k‐means clustering was used to describe the citrulline–iNOS trajectory in time‐lagged independent component analysis space (Figure S39). Binding free energies and per‐residue energy contributions were estimated using MM/GBSA [55].

### 4.11 Conditioned‐Medium Assays in TDSCs and BMSCs

#### 4.11.1 Conditioned‐Medium Preparation

LPS‐stimulated THP‐1‐derived macrophages were treated under each of the five patch conditions described in Section 4.6.2 for 48 h, washed with PBS, and incubated in serum‐free RPMI 1640 for 24 h. The resulting conditioned medium (CM) was filtered through a 0.22 µm membrane and applied to TDSCs or BMSCs.

#### 4.11.2 TDSC Migration and Tenogenic Responses

TDSC migration was assessed by Transwell assays at 24 and 48 h and by scratch wound‐healing assays at 24 h. Tenogenic responses were assessed at day 7 by immunofluorescence for COL1 and SCX and by qPCR for *SCX*, *TNMD*, and *COL1A1*.

#### 4.11.3 BMSC Osteogenic Responses

BMSC osteogenic responses were assessed at day 7 by immunofluorescence for RUNX2 and OCN, qPCR for *RUNX2*, *OPN*, and *COL1A1*, and ALP staining and activity assays. Mineralization was evaluated by Alizarin Red S staining at day 21.

### 4.12 Rat and Rabbit Rotator Cuff Tear Models

#### 4.12.1 Rat Model

An acute supraspinatus transection‐and‐repair model was established in male Sprague–Dawley rats (8 weeks old, 250–300 g). Animals were assigned to five repair groups: Control (suture repair only), pPLG, ApoTK@pPLG, CGQD@pPLG, and ACGQD@pPLG. Patches (approximately 5 × 5 mm) were placed at the repaired tendon‐to‐bone interface. Repair outcomes were assessed at 2 and 8 weeks, with six independent animals per group at each time point. An additional unoperated Sham group (*n* = 6) provided the 8‐week histological and gait references shown in Figures S42 and S43.

#### 4.12.2 Rabbit Model

An acute supraspinatus transection‐and‐repair model was established in male New Zealand white rabbits (12 weeks old, 2.5–3.0 kg). Rabbits were assigned to two groups (*n* = 6 per group): Control (suture repair only) and ACGQD@pPLG. Animals were euthanized 10 weeks after surgery for histological evaluation.

### 4.13 Histological, Biomechanical, and Functional Evaluation

#### 4.13.1 Histology and Matrix Quantification

Tendon–humerus blocks were fixed in 4% paraformaldehyde for 48 h, decalcified in 10% EDTA (pH 7.4) for 4–6 weeks, paraffin‐embedded, and sectioned at 5 µm. Sections were stained with H&E, Masson trichrome, Safranin O–fast green, toluidine blue, or picrosirius red and imaged by bright‐field or polarized‐light microscopy (Leica DM6). Five fields per section were analyzed in ImageJ. Matrix endpoints comprised Masson‐based relative collagen content, the metachromasia ratio (Safranin O staining normalized to the Control reference), and grayscale brightness of polarized picrosirius‐red images as a measure of collagen birefringence and organization [30]. COL I and COL III were assessed by immunohistochemistry, and COL II by immunofluorescence. The 8‐week unoperated reference is presented in Figure S42. Modified tendon healing score criteria are provided in Table S1 [56, 57, 58].

#### 4.13.2 Tendon–Bone Biomechanical Testing

The supraspinatus tendon–humerus complex was harvested with the proximal muscle and humeral shaft preserved. Specimens were tested on an HSS‐DX1000 biomechanical testing machine (Jinan Heng Rui Jin Testing Machine Co., Ltd., Jinan, China). The humeral head was potted in dental cement, and the muscle was secured in a soft‐tissue grip. Specimens were preloaded at 0.1 N and pulled at 10 mm min^−^^1^ to failure. Ultimate load to failure was recorded, stiffness (N mm^−^^1^) was calculated from the linear region of the load–displacement curve, and ultimate stress (N mm^−^^2^) was calculated as the ultimate load divided by the tendon cross‐sectional area measured with digital calipers. Six specimens per group were tested at each time point.

#### 4.13.3 Gait Analysis

Gait was assessed with the Shanghai Xinruan VisuGait system at 2 and 8 weeks. Forelimb pressure, contact area, stride length, stride width, contact phase ratio, and oscillatory phase ratio were recorded. Each measurement was normalized to the mean of the repair‐only Control group at the corresponding time point; the 8‐week Sham comparisons used the same denominator (Figure S43). Six independent animals per group were included, and animal‐level values were used for statistical analysis.

#### 4.13.4 Micro‐CT

The proximal humerus was scanned on a SkyScan 1276 system (Bruker; 80 kV, 100 µA, 1 mm Al filter, 8 µm voxel size). A volume of interest in the proximal humeral trabecular bone was analyzed in CTAn v1.20 for bone volume fraction (BV/TV), bone mineral density (BMD), trabecular number (Tb.N), and trabecular thickness (Tb.Th).

### 4.14 Local Fluorescence Imaging and Endpoint Safety Assessment

#### 4.14.1 Implant‐Site Fluorescence Imaging

A patch containing fluorescently labeled nanoparticles was placed at the repair site, and the shoulder region was imaged on days 0, 3, 7, and 10 using identical acquisition settings and a common display range (Figure S44).

#### 4.14.2 Eight‐Week Safety Endpoints

Whole blood was collected at the 8‐week endpoint. Complete blood counts were measured on a Sysmex XN‐1000 analyzer. Serum alanine aminotransferase (ALT), aspartate aminotransferase (AST), blood urea nitrogen (BUN), creatinine, and uric acid were measured on a Beckman AU5800 analyzer (Figure S46). The heart, liver, spleen, lungs, and kidneys were fixed, paraffin‐embedded, sectioned, and stained with H&E (Figure S45).

### 4.15 Statistical Analysis

Data are presented as mean ± SD, with individual data points shown where applicable. Two‐group comparisons were performed using two‐sided Student's *t*‐tests. Comparisons among three or more groups were performed using one‐way ANOVA with Tukey's post hoc test. Tests for individual analyses are specified in the corresponding figure legends. Analyses were performed in GraphPad Prism v10.0. *p* < 0.05 was considered statistically significant, and *p* values or reporting thresholds are shown in the figures.

## Author Contributions

**Yuhao Yu**: conceptualization, methodology, writing – review and editing, writing – original draft, visualization, validation, data curation, formal analysis. **Wencai Liu**: methodology, validation, formal analysis, data curation, visualization. **Xinyue Yang**: validation, visualization, methodology. **Hanzhe Zhang**: validation, methodology. **Hui Xu**: validation. **Xin Wang**: validation. **Weimin Lin**: validation. **Xiping Jiang**: validation. **Chenrui Yuan**: validation. **Yifei Wang**: validation. **Weilin Yu**: validation. **Yamin Li**: supervision, writing – review and editing, investigation, funding acquisition. **Wei Song**: funding acquisition, supervision, writing – review and editing, investigation. **Yaohua He**: funding acquisition, writing – review and editing, supervision, investigation, resources.

## Funding

This work was supported by the National Natural Science Foundation of China (grant No.82372441; 82572737; 82302694), the China Postdoctoral Science Foundation General Program (No. 2024M762032), the Shanghai Municipal Natural Science Foundation (No. 25ZR1402422) and Youth Cultivation Program of Shanghai Sixth People's Hospital (No. ynqn202505).

## Conflicts of Interest

The authors declare no conflicts of interest.

## Supporting information




**Supporting File**: advs78630‐sup‐0001‐SuppMat.pdf.

## Data Availability

All data supporting the results of this study are available within the Main Text and Supporting Information or with the corresponding author on request. The bulk RNA‐sequencing data generated in this study have been deposited in the NCBI Sequence Read Archive (SRA) under BioProject accession PRJNA1465821.
