# Supplementary material for: A Citrulline‐Functionalized Patch Coordinates Macrophage NRF2 Signaling and Arginine Metabolism for Tendon‐to‐Bone Repair
Source: Adv Sci (Weinh). 2026 Nov 9:e78630. Online ahead of print. doi: 10.1002/advs.78630 (PMC13749005; doi:10.1002/advs.78630)
Supplement: Supplementary file 1 [file ADVS-9999-e78630-s001.pdf]

Supporting Information

A Citrulline-Functionalized Patch Coordinates Macrophage NRF2 Signaling and Arginine Metabolism for Tendon-to-Bone Repair

Yuhao Yu[1]†, Wencai Liu[1]†, Xinyue Yang[1], Hanzhe Zhang[1], Hui Xu[1], Xin Wang[1], Weimin Lin[1], Xiping Jiang[1], Chenrui Yuan[1], Yifei Wang[1], Weilin Yu[1], Yamin Li[1], Wei Song[1]*, Yaohua He[1]**

[1] Department of Orthopedic Surgery, Shanghai Sixth People's Hospital Affiliated to Shanghai Jiao Tong University School of Medicine, Shanghai 200233, China

*Corresponding author. Email: xiaokui0621@126.com; songw1995@sjtu.edu.cn; heyaohua@sjtu.edu.cn

†These authors contributed equally to this work.

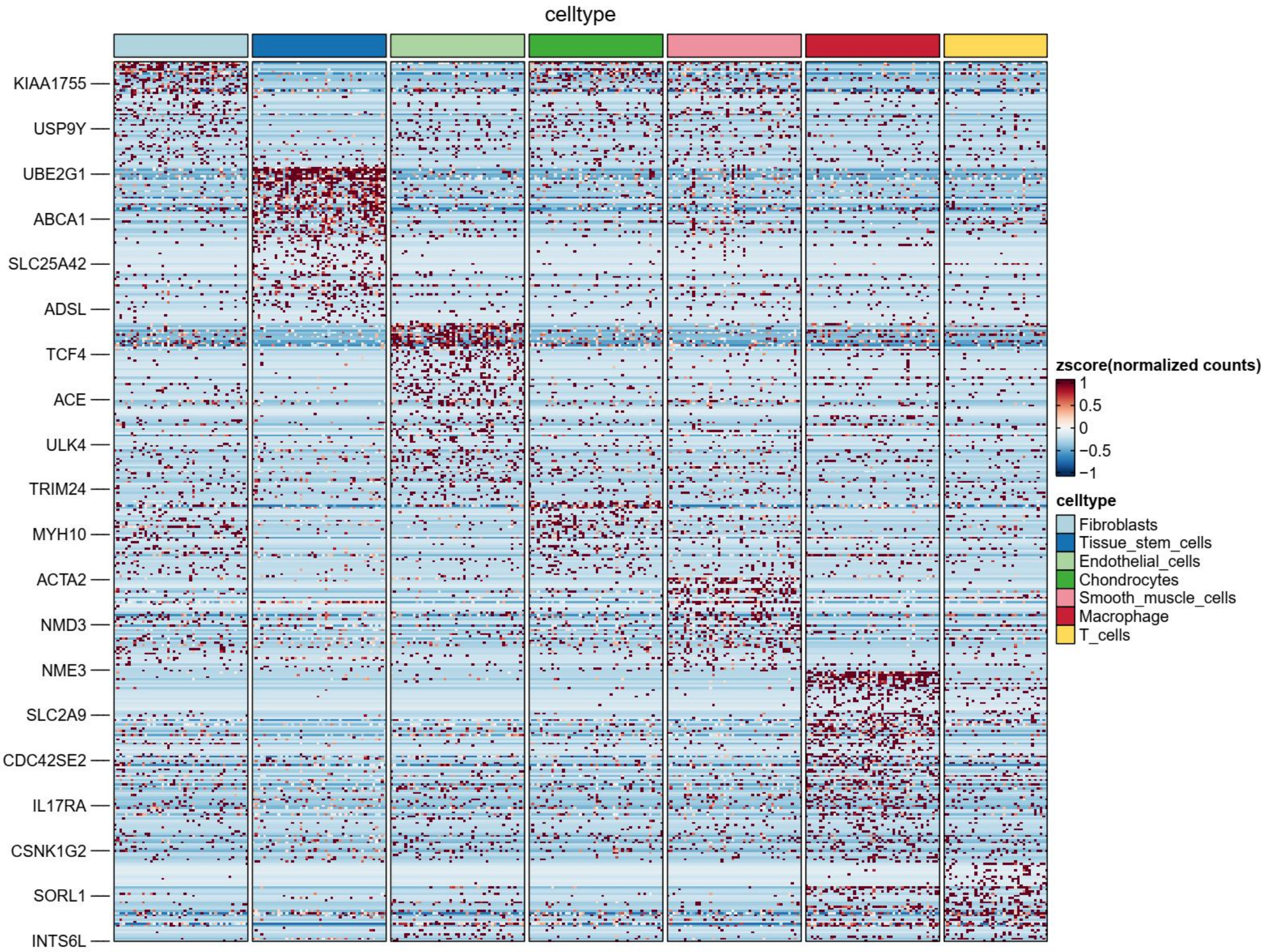

Figure S1. Marker-gene expression across the major cell populations identified by single-cell RNA sequencing. Rows show selected marker genes, and columns show individual cells grouped by their annotated population: fibroblasts, tissue stem cells, endothelial cells, chondrocytes, smooth muscle cells, macrophages, and T cells.

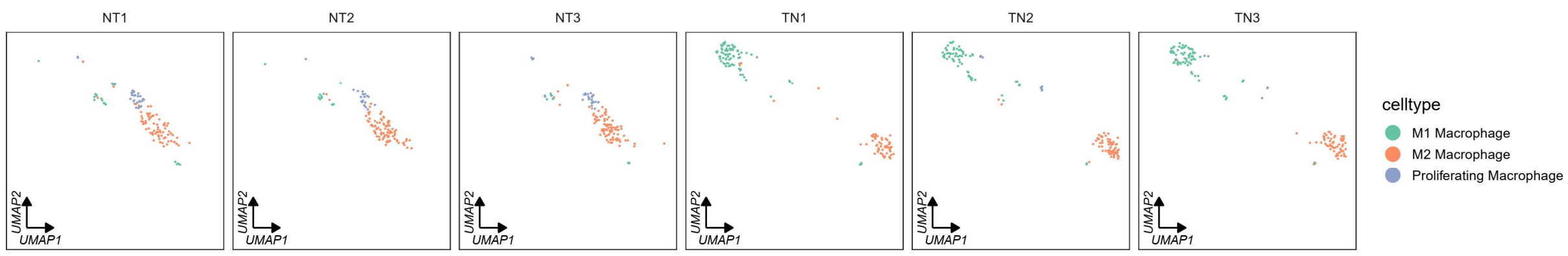

Figure S2. Donor-resolved UMAP visualization of macrophage populations in clinical tendon specimens. Macrophages from non-injured comparison tendons (NT1–NT3) and injured tendons (TN1–TN3) are displayed separately on the same UMAP embedding. Each point represents one cell; green, orange, and blue denote populations annotated as M1-like, M2-like, and proliferating macrophages, respectively.

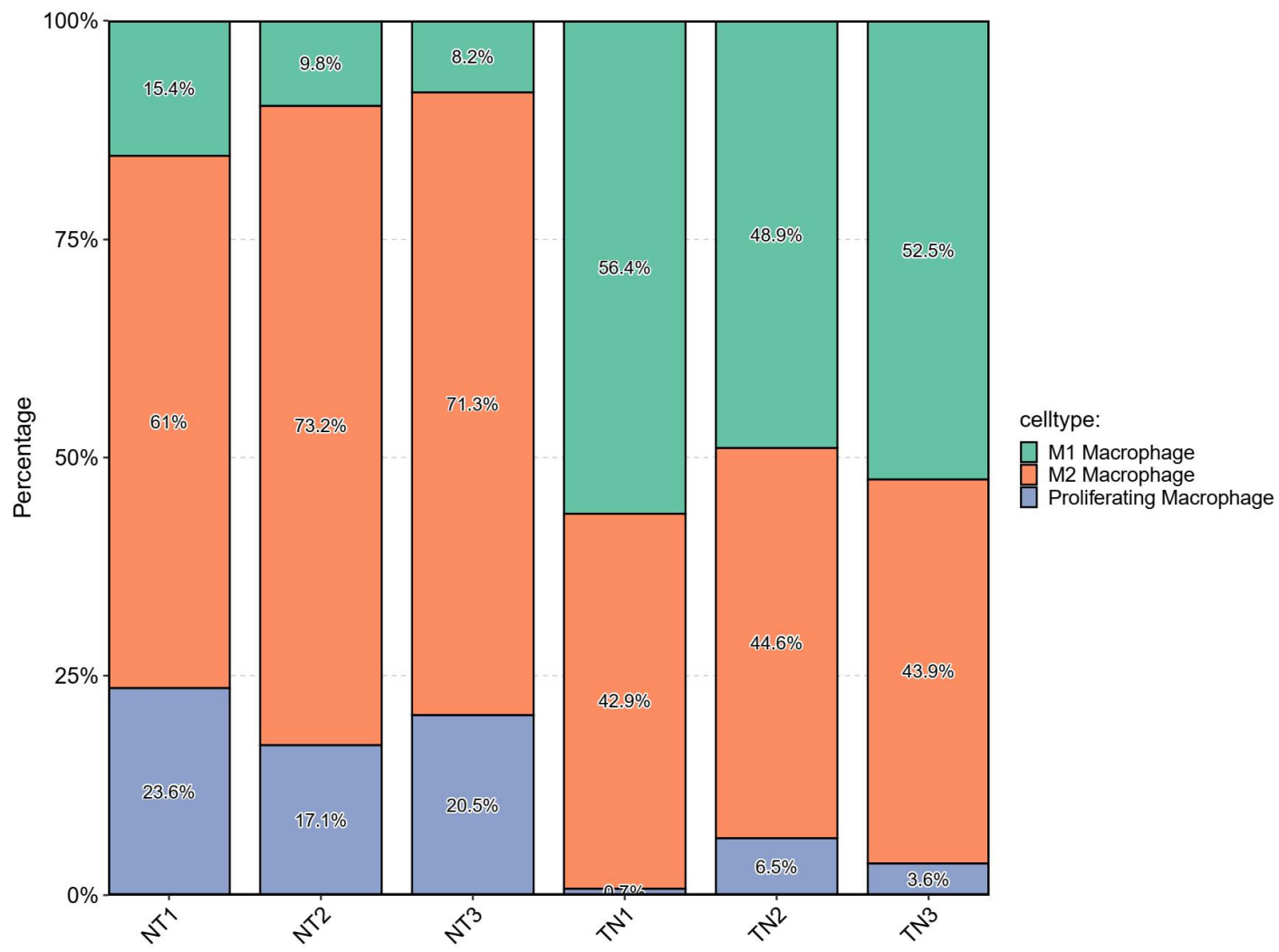

Figure S3. Sample-level proportions of the annotated macrophage populations. Stacked bars show the percentages of cells annotated as M1-like, M2-like, and proliferating macrophages within the macrophage compartment of each non-injured comparison sample (NT1–NT3) and injured sample (TN1–TN3).

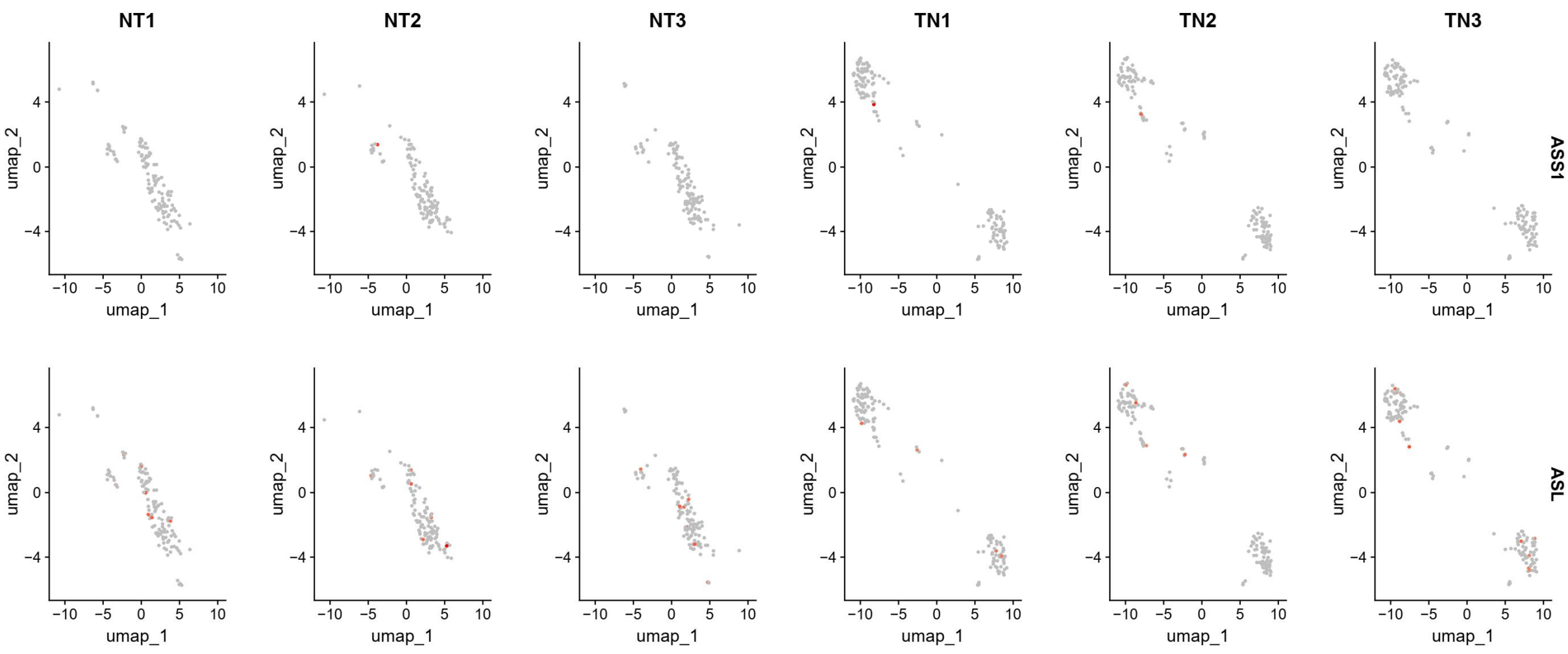

Figure S4. Donor-resolved UMAP feature plots of *ASS1* and *ASL* expression in macrophages. *ASS1* (upper row) and *ASL* (lower row) expression is shown separately for NT1–NT3 and TN1–TN3 on the same UMAP embedding. Each point represents one macrophage; gray denotes cells without a displayed expression signal, and colored points indicate expression of the labeled gene. NT denotes non-injured comparison tendon specimens, and TN denotes injured tendon specimens.

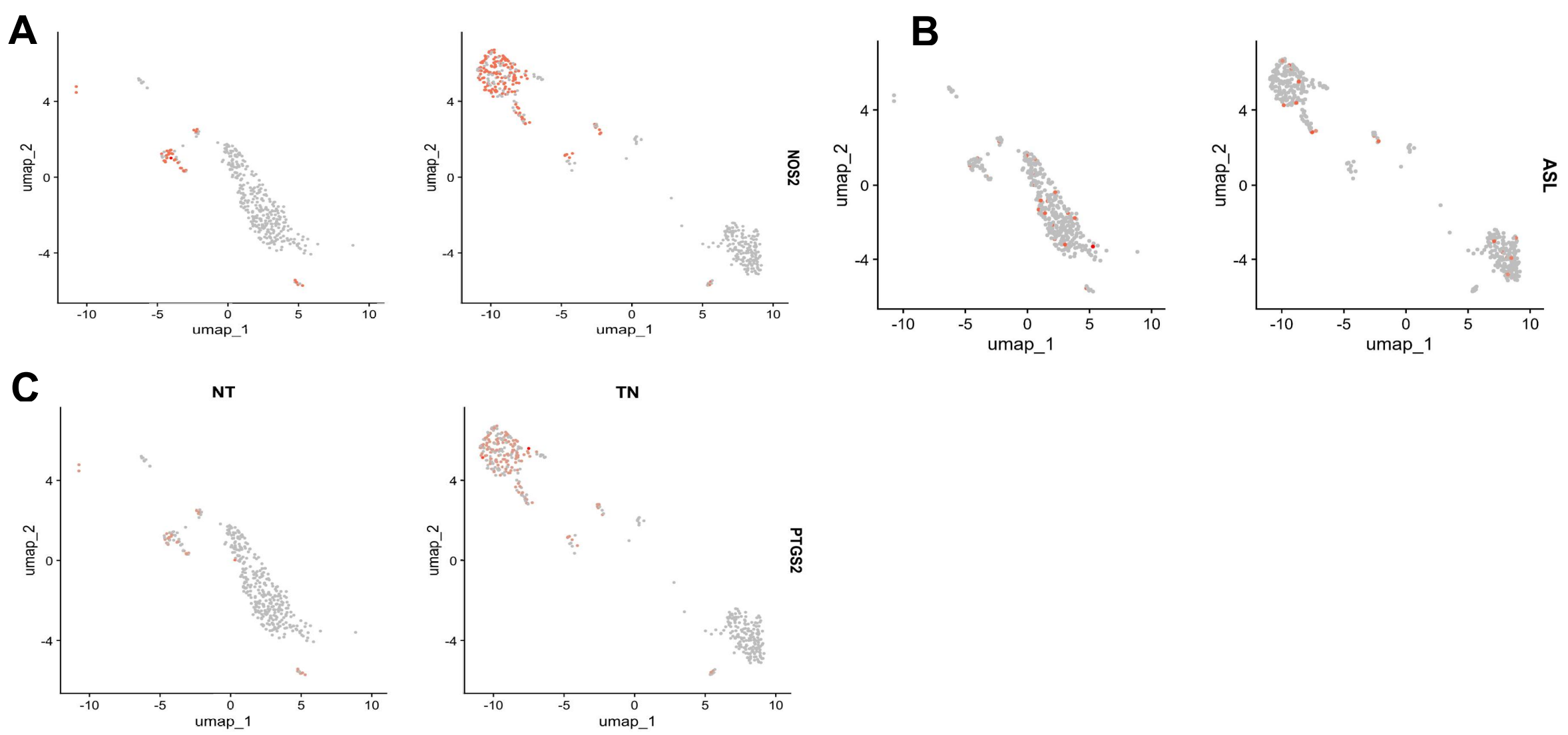

Figure S5. UMAP feature plots of macrophage gene expression in clinical tendon specimens. Expression of (A) *NOS2*, (B) *ASL*, and (C) *PTGS2* is shown in non-injured comparison tendon (NT; left) and injured tendon (TN; right) macrophages. Each point represents one cell, and color intensity reflects log-normalized expression of the indicated gene.

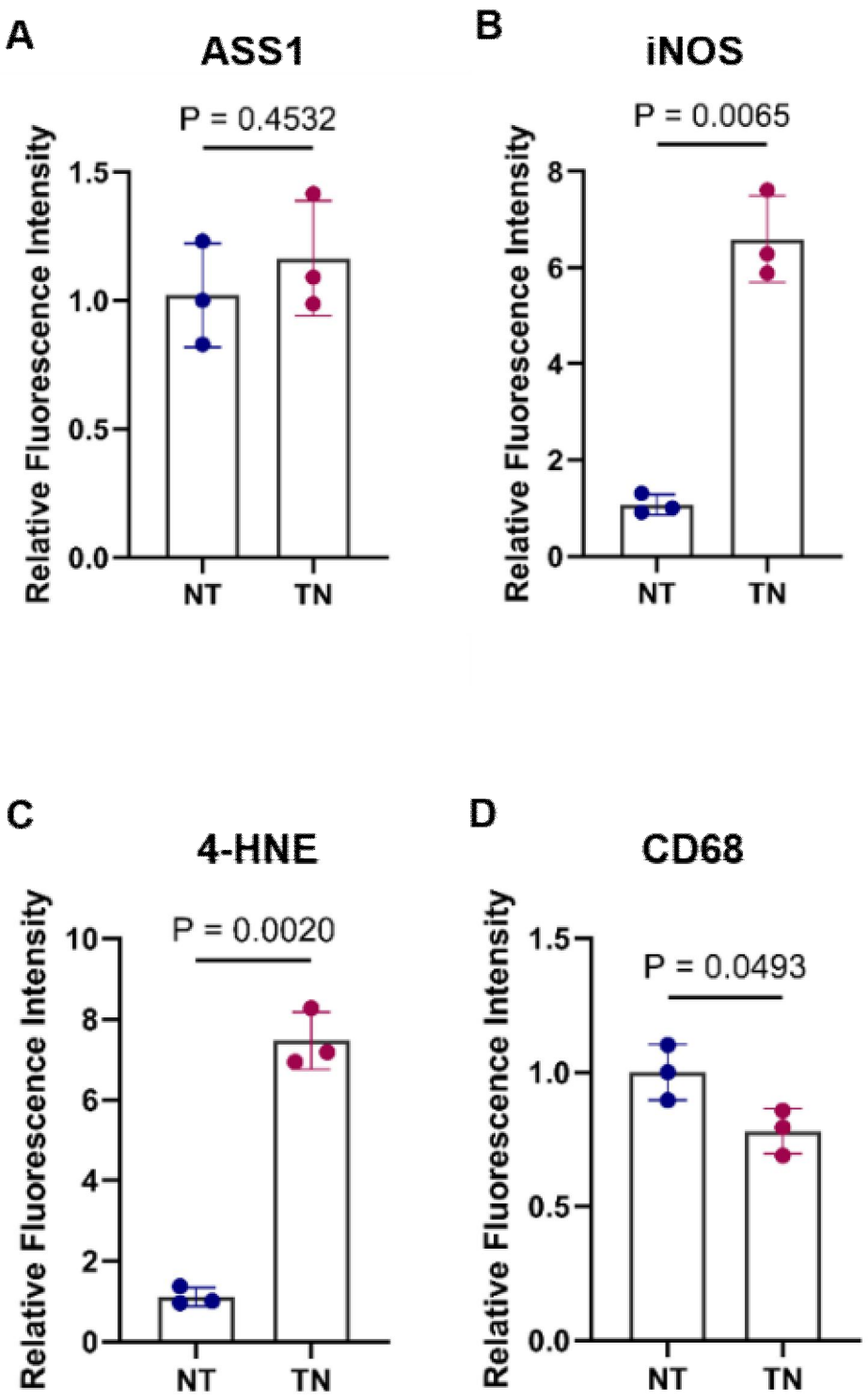

Figure S6. Semiquantitative immunofluorescence analysis of clinical tendon specimens. Relative fluorescence intensities of (A) argininosuccinate synthetase 1 (ASS1), (B) inducible nitric oxide synthase (iNOS), (C) 4-hydroxynonenal (4-HNE), and (D) CD68 are compared between non-injured comparison tendons (NT) and injured tendons (TN). Data are mean ± SD; n = 3 specimens per group. Individual values and comparison P values are shown.

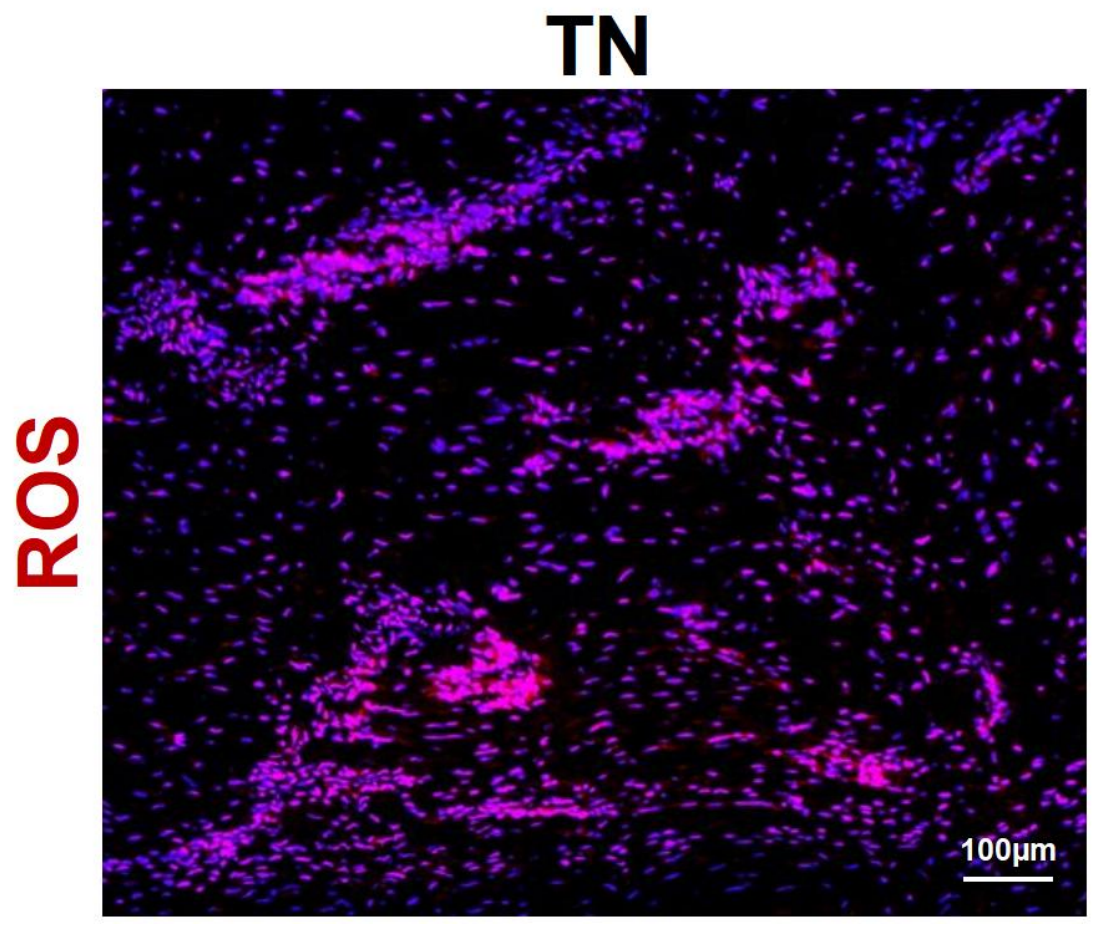

Figure S7. Fluorescence staining of oxidative stress in a clinically injured tendon specimen. The ROS-associated fluorescence signal is shown in magenta, and nuclei are counterstained with DAPI (blue). Scale bar, 100 μm.

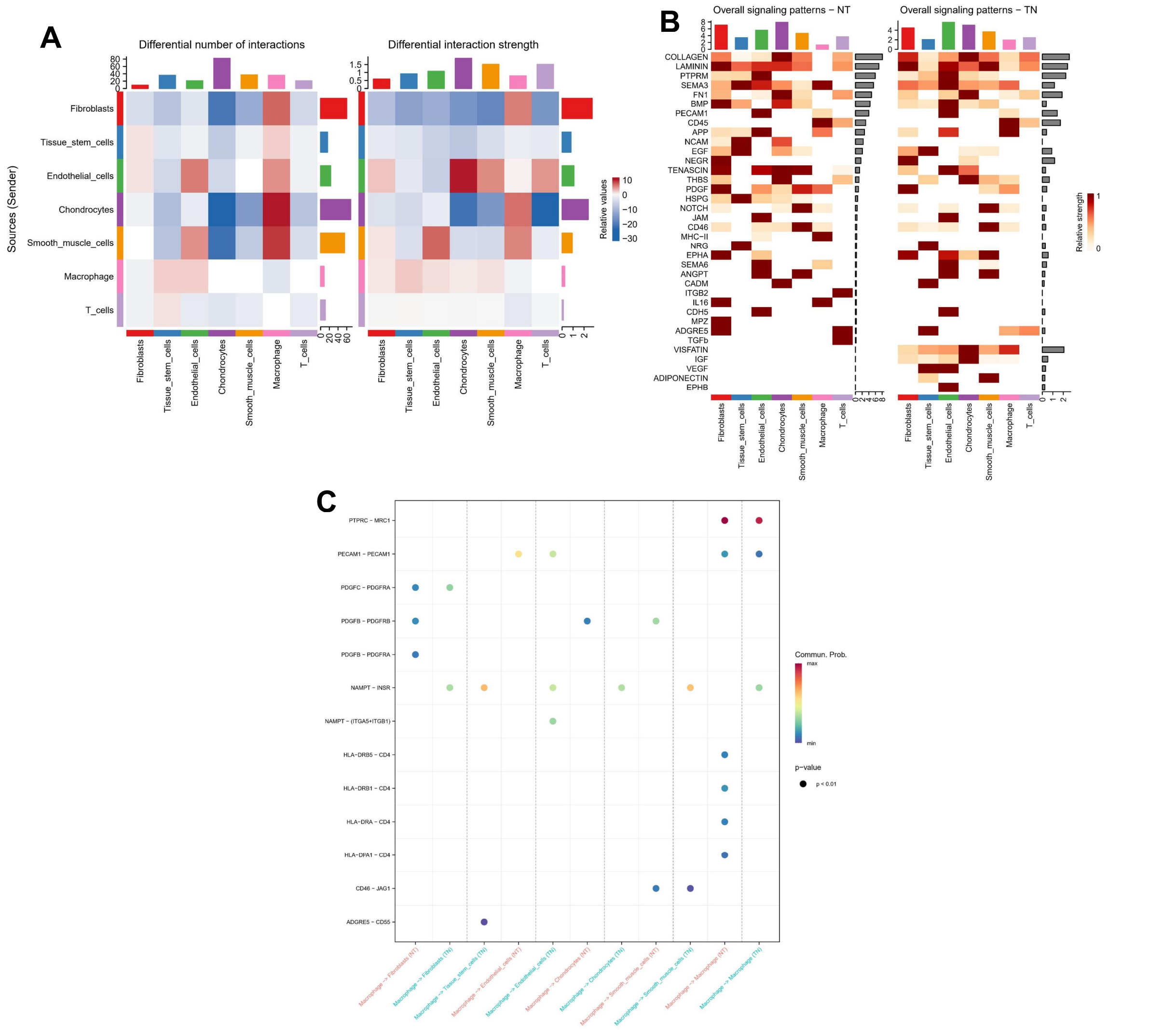

Figure S8. Inferred cell–cell communication in non-injured comparison tendon (NT) and injured tendon (TN). (A) Heatmaps showing differences between TN and NT in the number (left) and strength (right) of inferred interactions among fibroblasts, tissue stem cells, endothelial cells, chondrocytes, smooth muscle cells, macrophages, and T cells. Rows and columns represent sender and receiver populations, respectively. (B) Overall signaling-pattern heatmaps for NT and TN; color indicates relative signaling strength. (C) Bubble plot of inferred macrophage-derived ligand–receptor interactions with the indicated receiver populations in NT and TN. Bubble color indicates communication probability; only interactions with $P < 0.01$ are plotted. Cell–cell communication was inferred from single-cell transcriptomic data using CellChat.

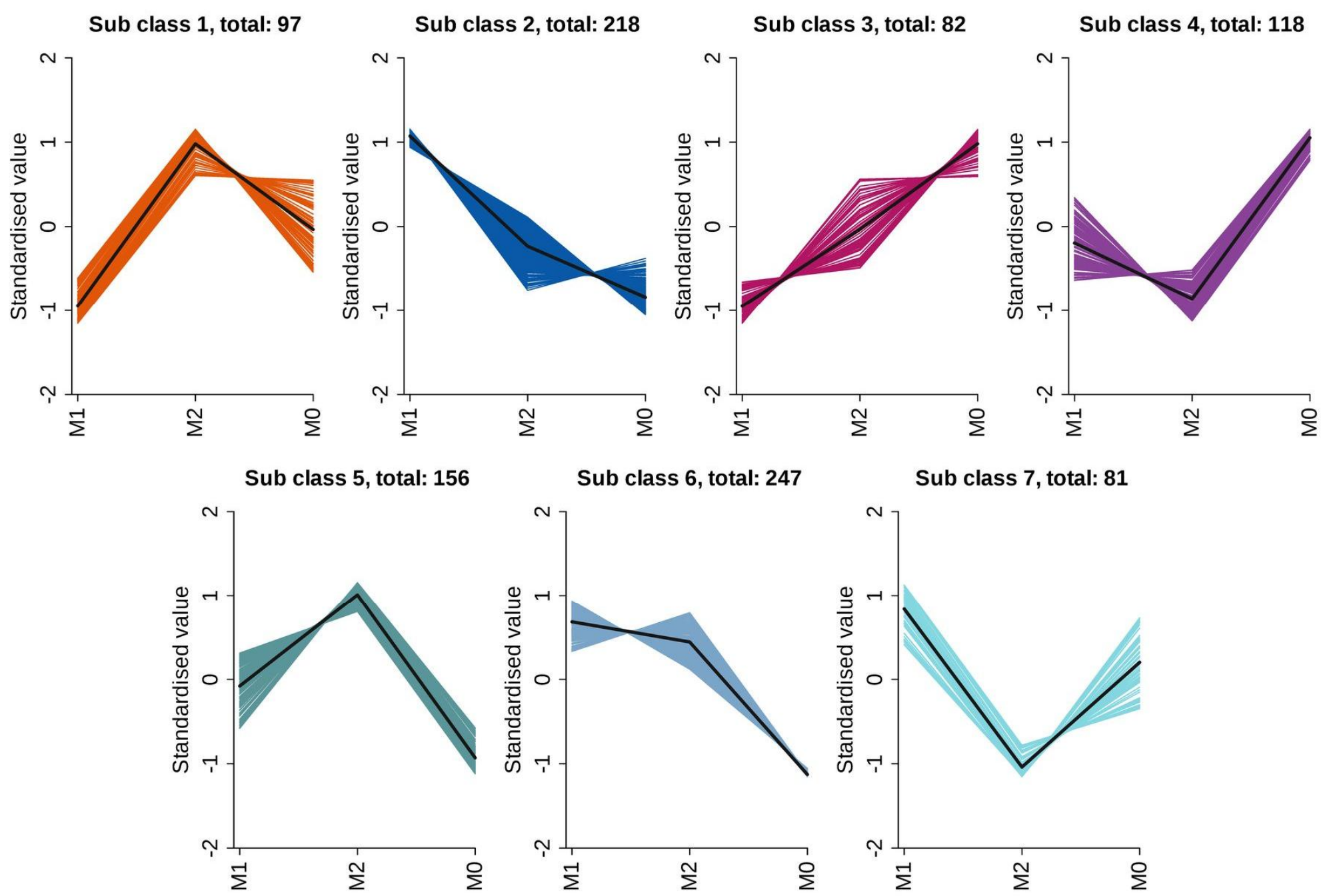

Figure S9. Mfuzz clustering of standardized metabolite-abundance profiles across macrophage states. Seven clusters contain 97, 218, 82, 118, 156, 247, and 81 metabolites, respectively. Profiles are shown in the order M1, M2, and M0 macrophages. Colored lines represent individual metabolite profiles, and black lines show the corresponding cluster-average profiles. Values were standardized before clustering.

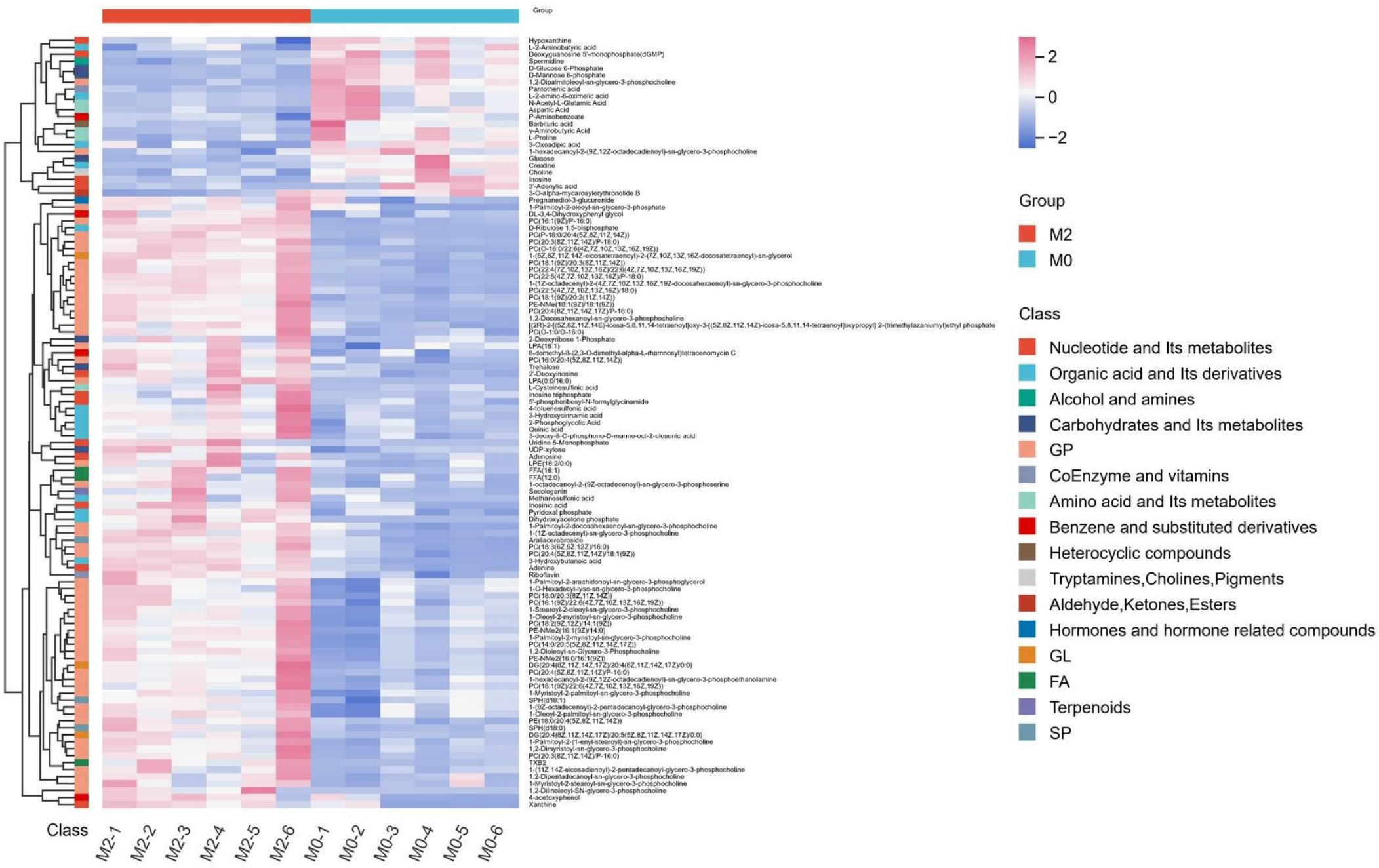

Figure S10. Heatmap of differential metabolite abundance between M2 and M0 macrophages. Differential metabolites selected using |log2FC| > 0.5, VIP > 1 and $P < 0.05$ are displayed after hierarchical clustering. Columns represent six samples per group, and rows represent metabolites. The upper color bar indicates macrophage state, and the side color bar indicates metabolite class. Red and blue denote higher and lower standardized relative abundance, respectively.

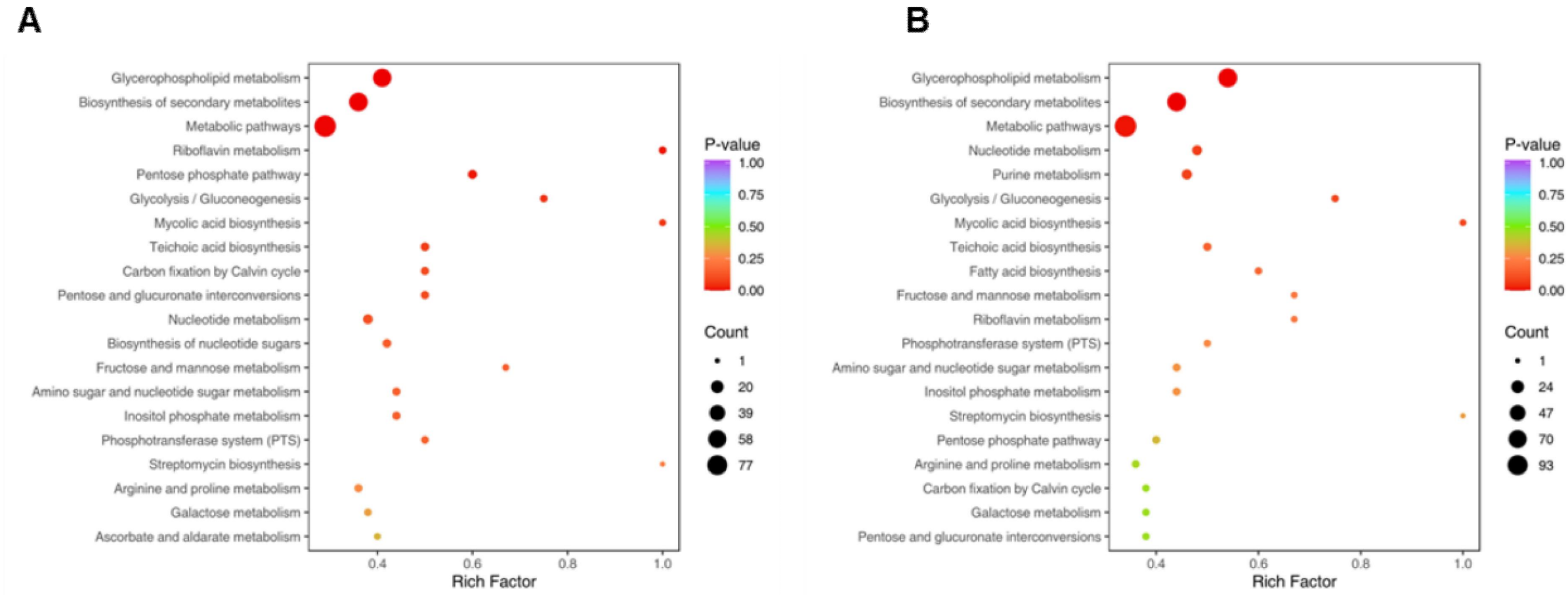

Figure S11. KEGG pathway enrichment of differential metabolites in macrophages. Enrichment results are shown for (A) M2 versus M0 macrophages and (B) M1 versus M0 macrophages. Bubble size indicates the number of differential metabolites assigned to a pathway, and bubble color indicates the enrichment *P* value. The rich factor summarizes the fraction of pathway-associated metabolites represented by the differential-metabolite set.

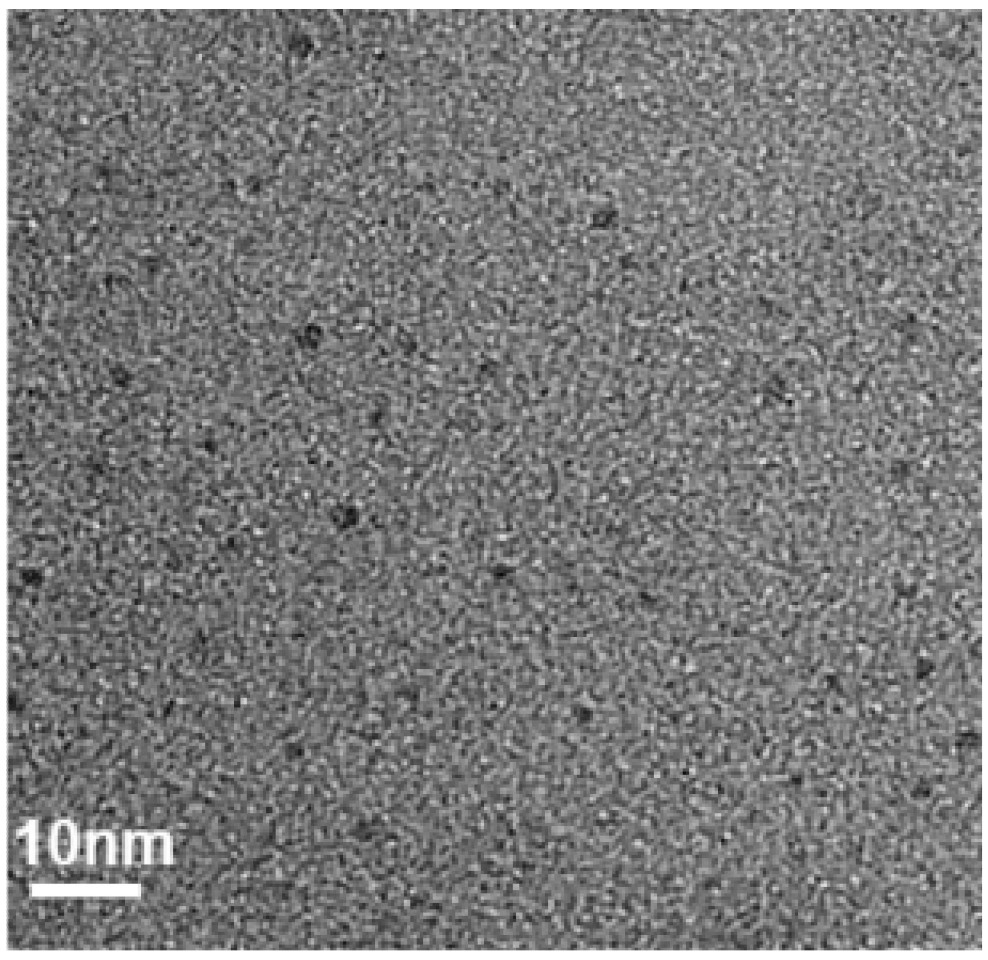

Figure S12. Transmission electron microscopy of glutathione-derived carbon dots (GQD). Representative TEM image of the GQD preparation. Scale bar, 10 nm.

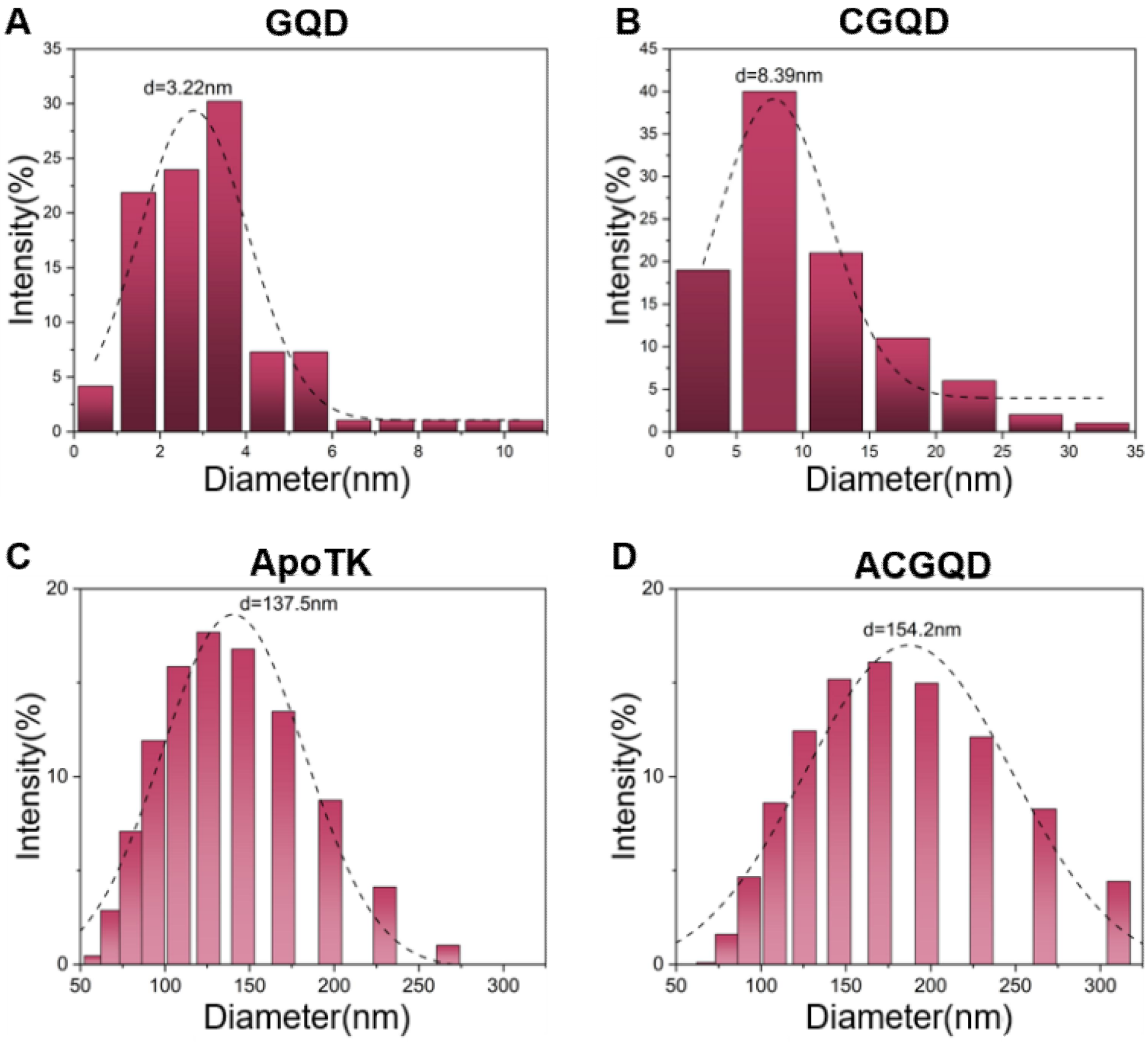

Figure S13. Hydrodynamic size distributions of the nanoparticle formulations. Dynamic light scattering measurements are shown for (A) GQD, (B) citrulline-functionalized GQD (CGQD), (C) the membrane preparation containing DSPE–PEG–TK (ApoTK), and (D) membrane-coated CGQD (ACGQD). The displayed diameters are 3.22, 8.39, 137.5, and 154.2 nm, respectively. Distributions are presented on an intensity basis.

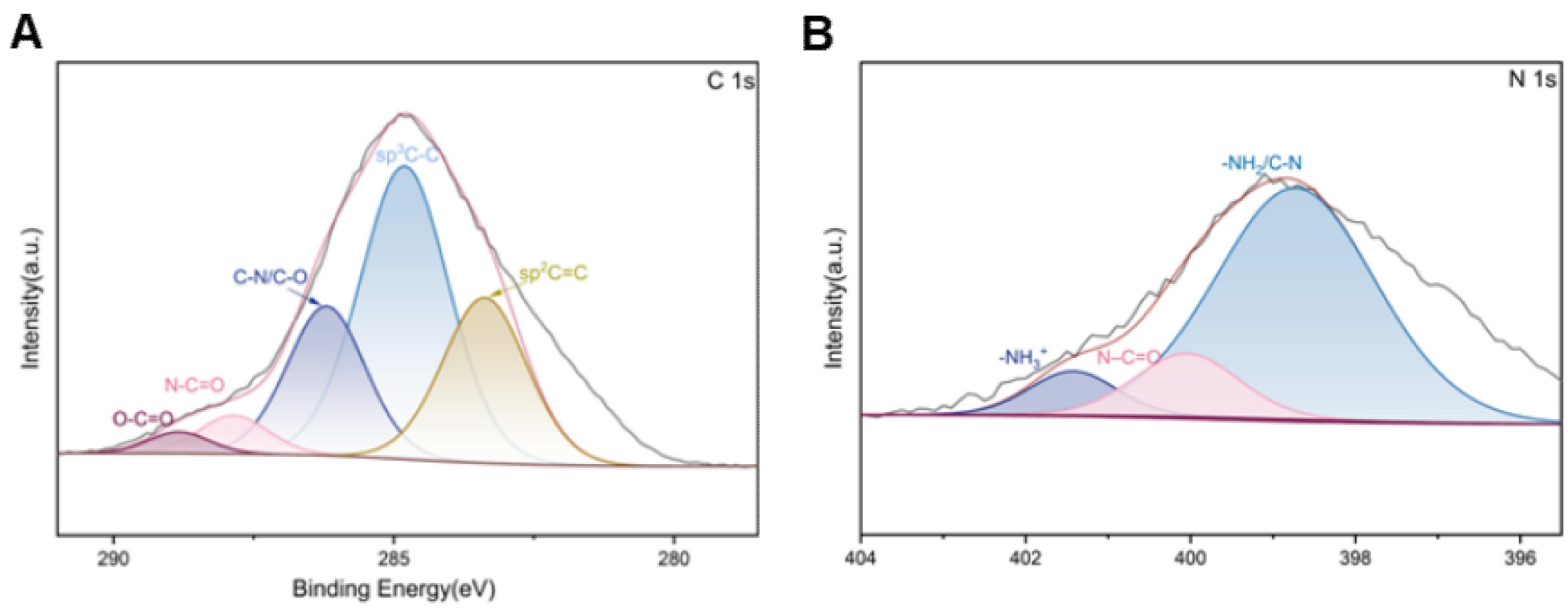

Figure S14. High-resolution X-ray photoelectron spectroscopy of GQD. (A) C 1s spectrum with the fitted carbon chemical environments labeled in the panel: sp^2 C=C, sp^3 C–C, C–N/C–O, N–C=O, and O–C=O. (B) N 1s spectrum with the fitted nitrogen environments labeled as –NH_2/C–N, N–C=O, and –NH_3^+. Colored components show the individual fitted contributions.

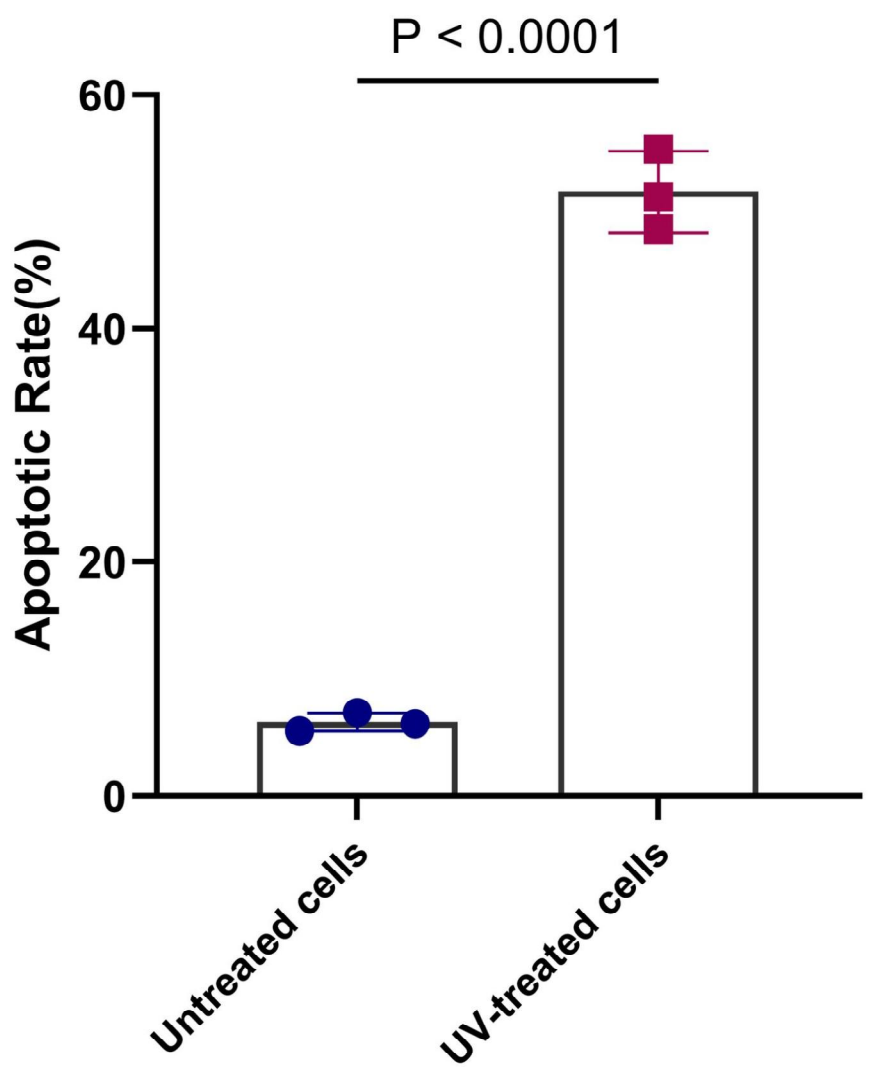

Figure S15. Apoptosis in the TDSC populations used for membrane preparation. The apoptotic-cell fraction in untreated and UV-treated tendon-derived stem cells (TDSCs) was assessed by Annexin V/propidium iodide flow cytometry. UV-treated cells were used without subsequent sorting or enrichment, so the membrane source contained both apoptotic and non-apoptotic cells. Data are mean ± SD; $n = 3$. Statistical comparison: two-sided Student's t-test; $P < 0.0001$.

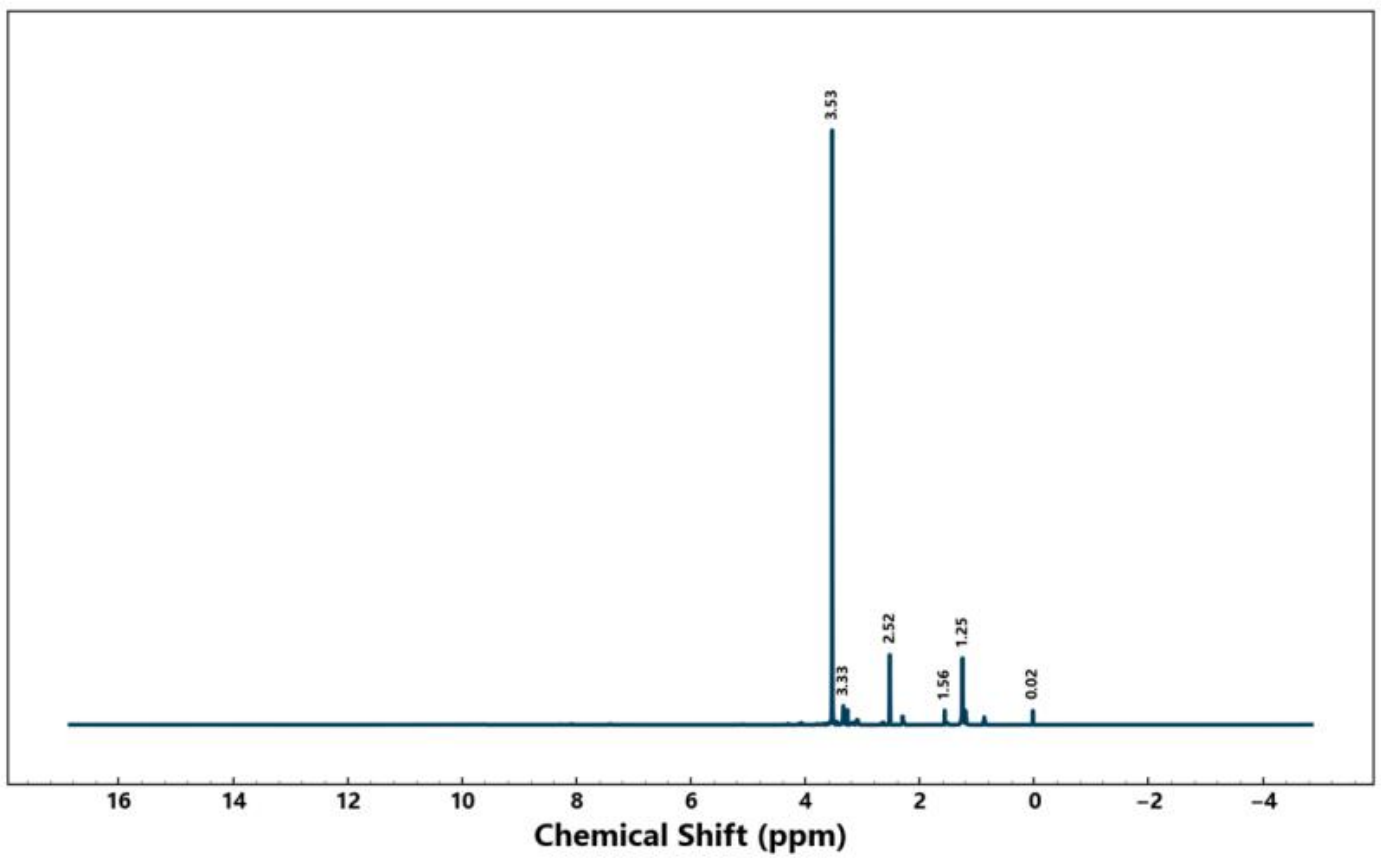

Figure S16. Proton nuclear magnetic resonance spectrum of DSPE–PEG–TK. The spectrum was recorded at 600 MHz in DMSO-d_6. The signal at δ 3.53 ppm corresponds to the poly(ethylene glycol) methylene protons, and those at δ 1.25 and 1.56 ppm to the stearoyl methylene protons; the signals at δ 2.52 and 3.33 ppm arise from residual DMSO and water, respectively.

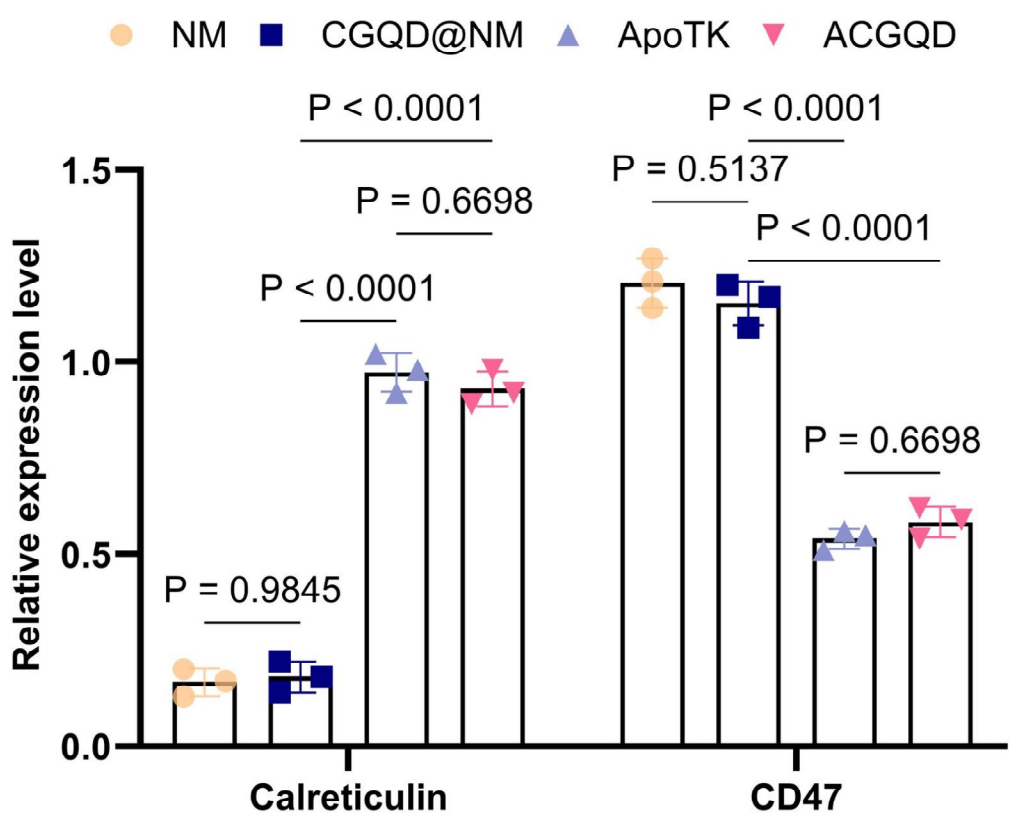

Figure S17. Densitometric analysis of calreticulin and CD47 in NM, CGQD@NM, ApoTK, and ACGQD. Calreticulin and CD47 band intensities were normalized to Na^+/K^+-ATPase. NM denotes the membrane preparation from untreated TDSCs. Data are mean ± SD; $n = 3$ per group. P values for the indicated comparisons were calculated by one-way ANOVA with Tukey's post hoc test.

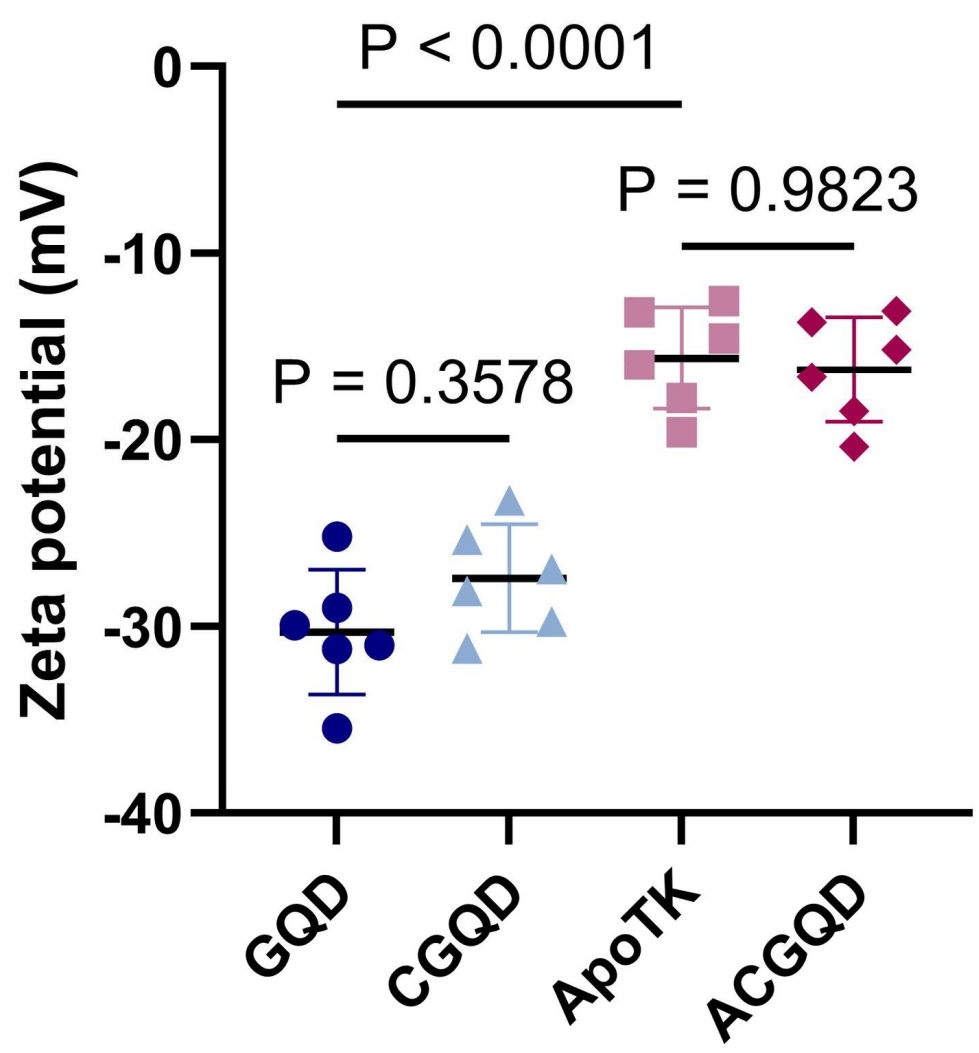

Figure S18. Zeta potentials of the nanoparticle formulations during assembly. Zeta potentials of GQD, CGQD, ApoTK, and ACGQD are compared. ApoTK denotes the DSPE–PEG–TK-containing membrane preparation from unsorted UV-treated TDSCs, and ACGQD denotes CGQD encapsulated in that preparation. Citrulline coupling did not significantly change the zeta potential, whereas membrane coating shifted it toward neutrality. Data are mean ± SD; $n = 6$ per formulation. P values for the indicated comparisons were calculated by one-way ANOVA with Tukey's post hoc test.

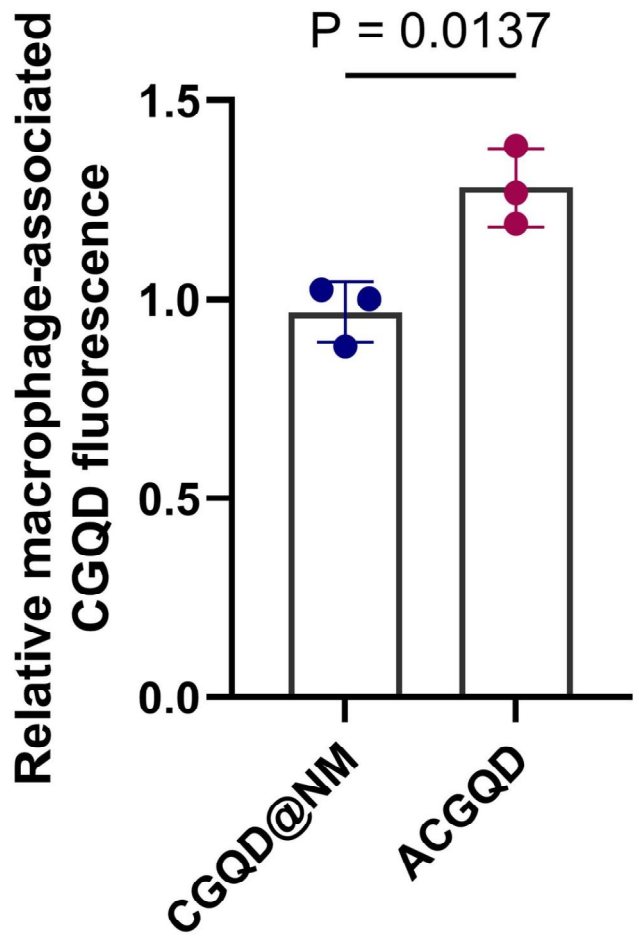

Figure S19. Semiquantification of macrophage-associated CGQD fluorescence. Relative CGQD fluorescence intensities are compared between macrophages exposed to CGQD@NM and ACGQD. CGQD@NM is coated with membranes from untreated TDSCs, whereas ACGQD contains membranes isolated from unsorted UV-treated TDSCs. Data are mean ± SD; $n = 3$. Individual values and the comparison P value are shown.

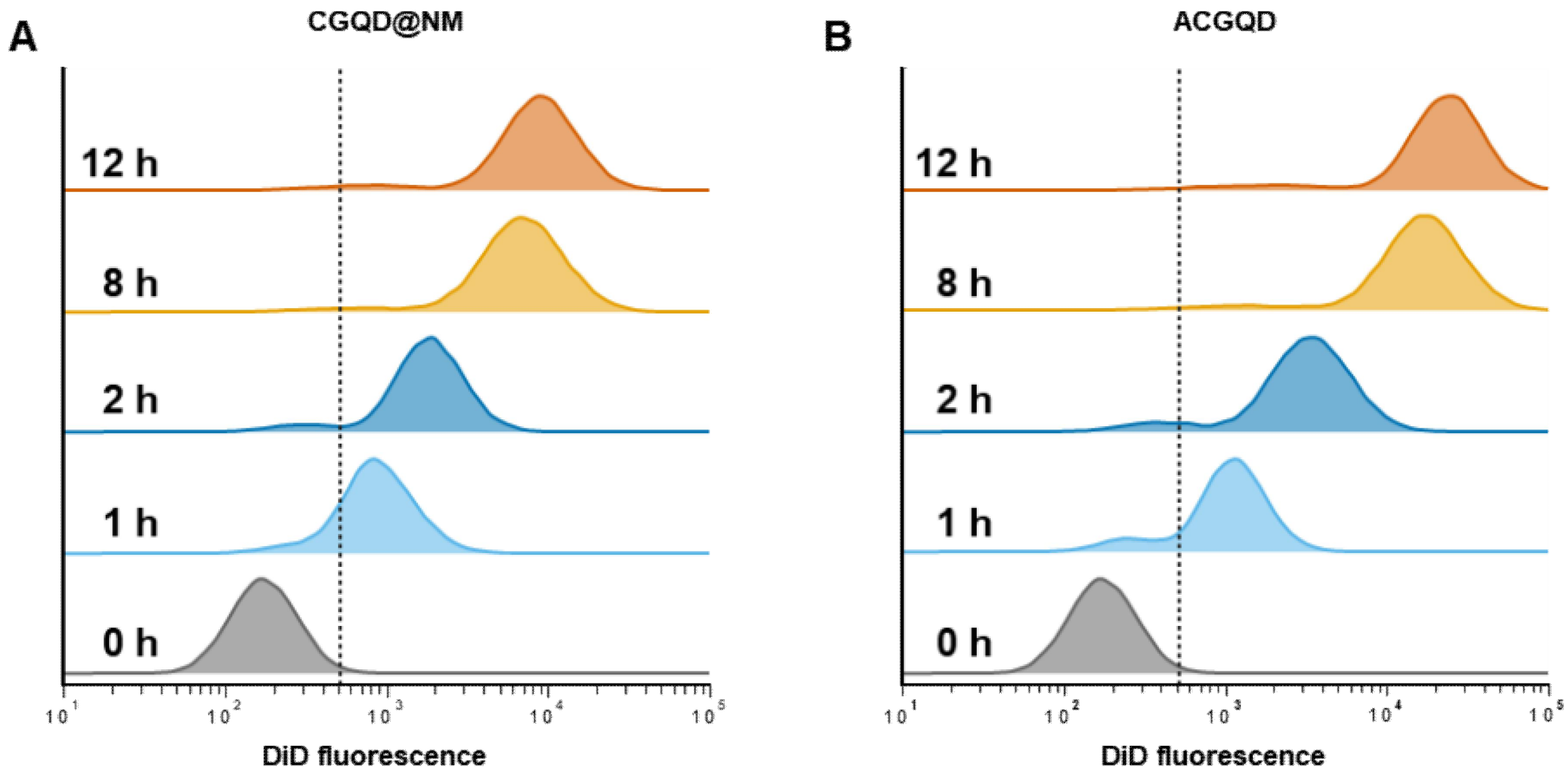

Figure S20. Time-dependent macrophage-associated fluorescence measured by flow cytometry. Histograms show DiD fluorescence in macrophages incubated with (A) CGQD@NM or (B) ACGQD for 1, 2, 8, and 12 h, with the 0 h distribution shown as the baseline. DiD labels the nanoparticle membrane coating. CGQD@NM contains membranes from untreated TDSCs, whereas ACGQD contains membranes from unsorted UV-treated TDSCs. The vertical dashed line in each panel marks the reference fluorescence level.

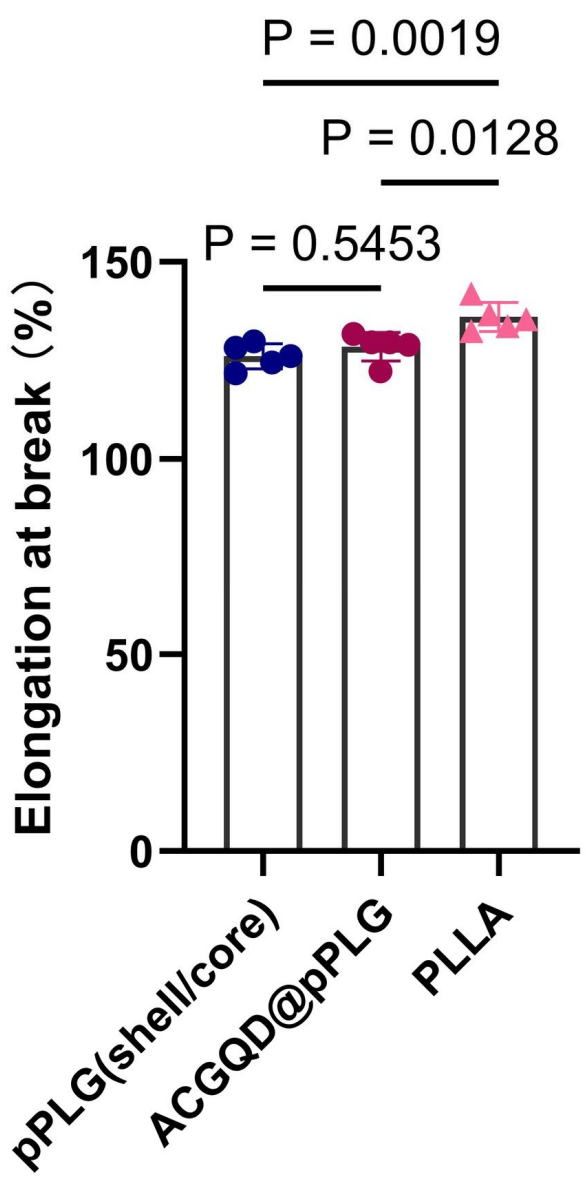

Figure S21. Elongation-at-break comparison of the electrospun patch formulations. Values are shown for pPLG (shell/core), ACGQD@pPLG, and PLLA patches after conditioning in PBS at 37 °C. Data are mean ± SD; *n* = 5 per formulation. Individual values and comparison *P* values are shown.

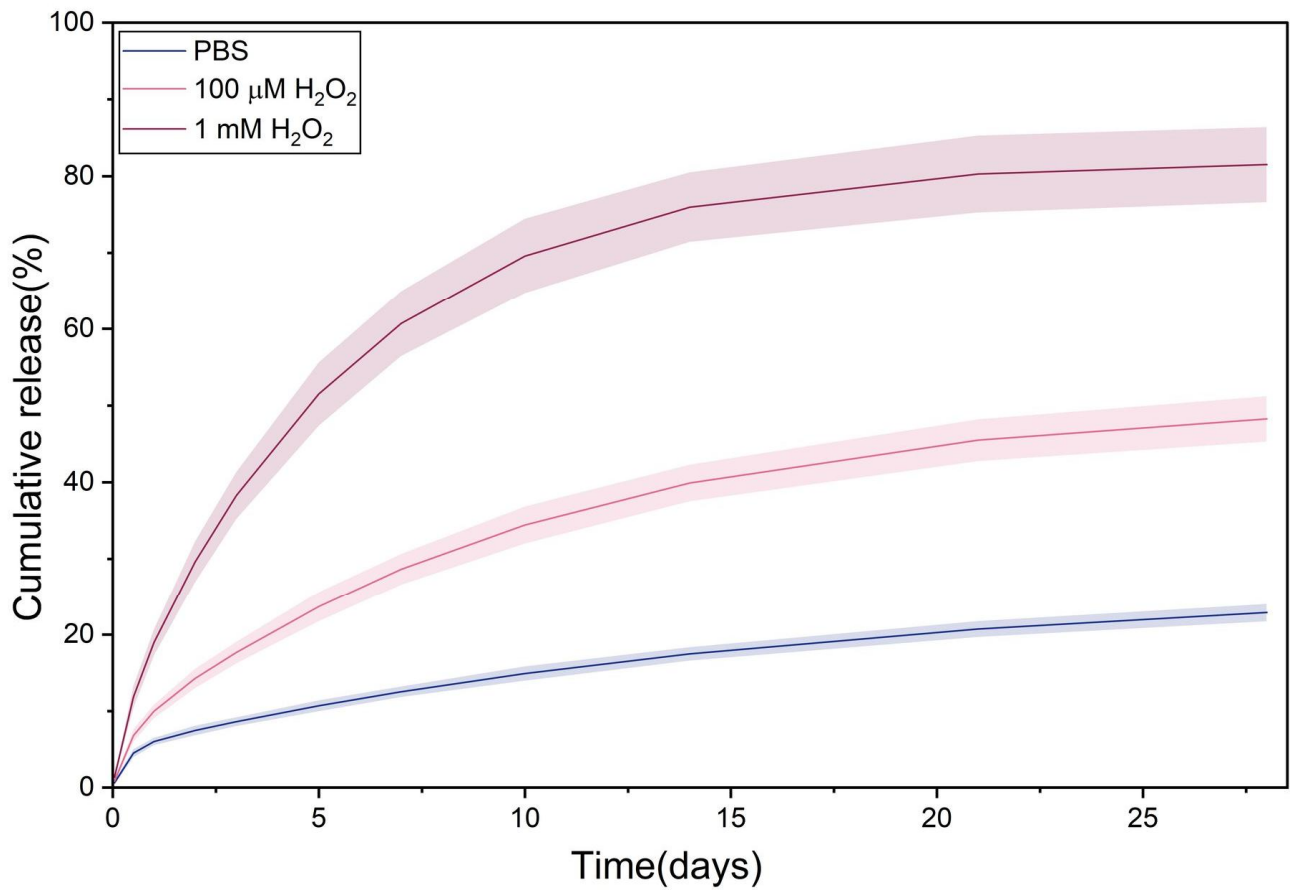

Figure S22. H_2O_2-responsive release of CGQD from ACGQD. Cumulative CGQD release was measured over 28 days in phosphate-buffered saline (PBS), 100 µM H_2O_2, or 1 mM H_2O_2. Solid lines show means and shaded bands indicate standard deviations; *n* = 6.

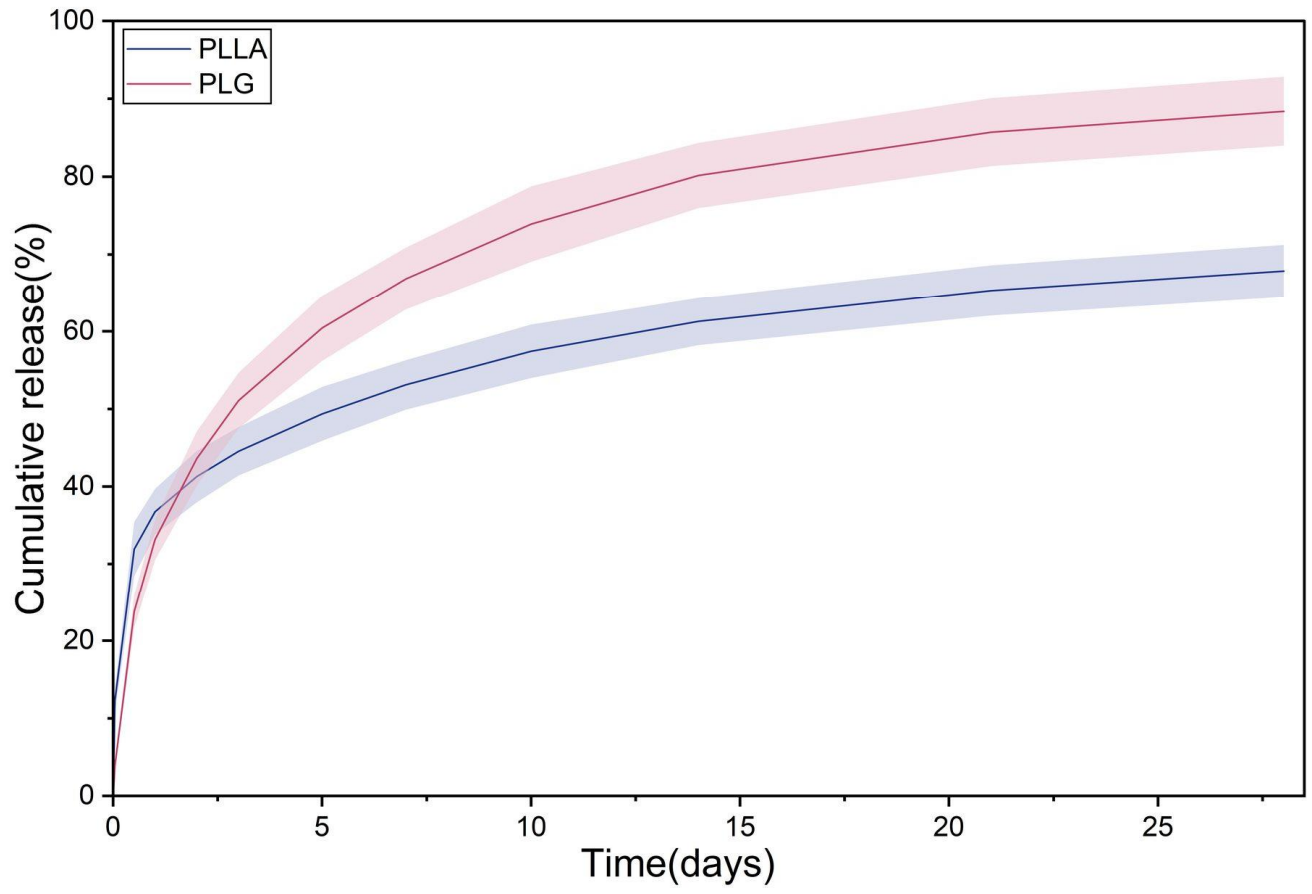

Figure S23. Cumulative release of ACGQD-associated cargo from PLLA and PLG fiber scaffolds over 28 days. Solid lines show means and shaded bands indicate standard deviations; *n* = 6.

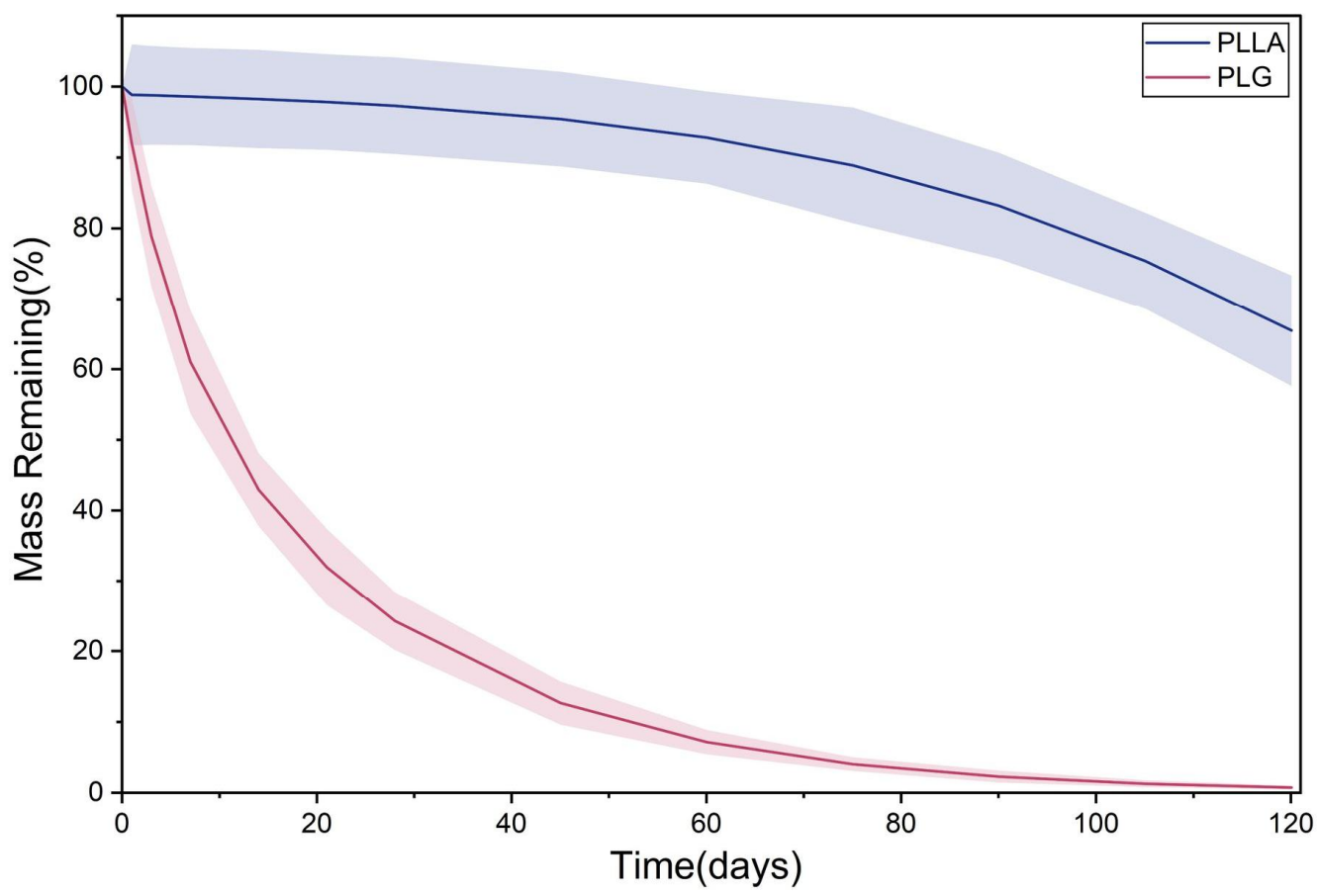

Figure S24. Mass remaining of PLLA and PLG scaffolds during 120 days of incubation. Residual mass is expressed as a percentage of the initial mass. Solid lines show means and shaded bands indicate standard deviations; *n* = 6.

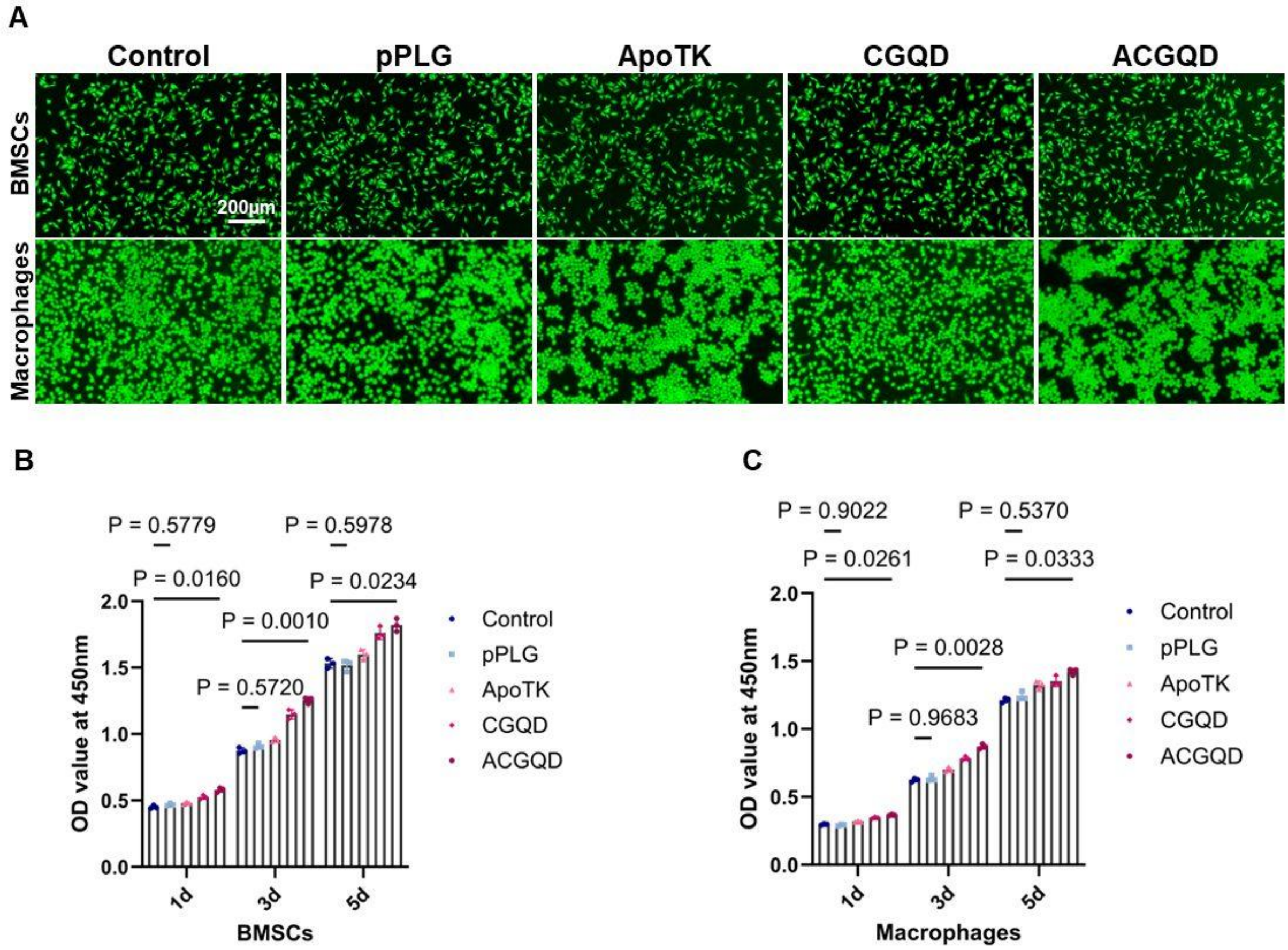

Figure S25. Cytocompatibility of the patch formulations in bone marrow-derived mesenchymal stem cells (BMSCs) and macrophages. (A) Representative calcein-AM fluorescence images of BMSCs (top row) and macrophages (bottom row) after 5 days of Transwell co-culture with the Control, pPLG, ApoTK, CGQD, and ACGQD formulations. ApoTK, CGQD, and ACGQD label the corresponding cargo-loaded pPLG patches. Green fluorescence indicates calcein-AM-positive cells. Scale bar, 200 μm. (B, C) Cell Counting Kit-8 (CCK-8) absorbance at 450 nm for BMSCs (B) and macrophages (C) on days 1, 3, and 5. Data are mean ± SD with individual values shown; $n = 3$. P values for the indicated comparisons were calculated by one-way ANOVA with Tukey's post hoc test.

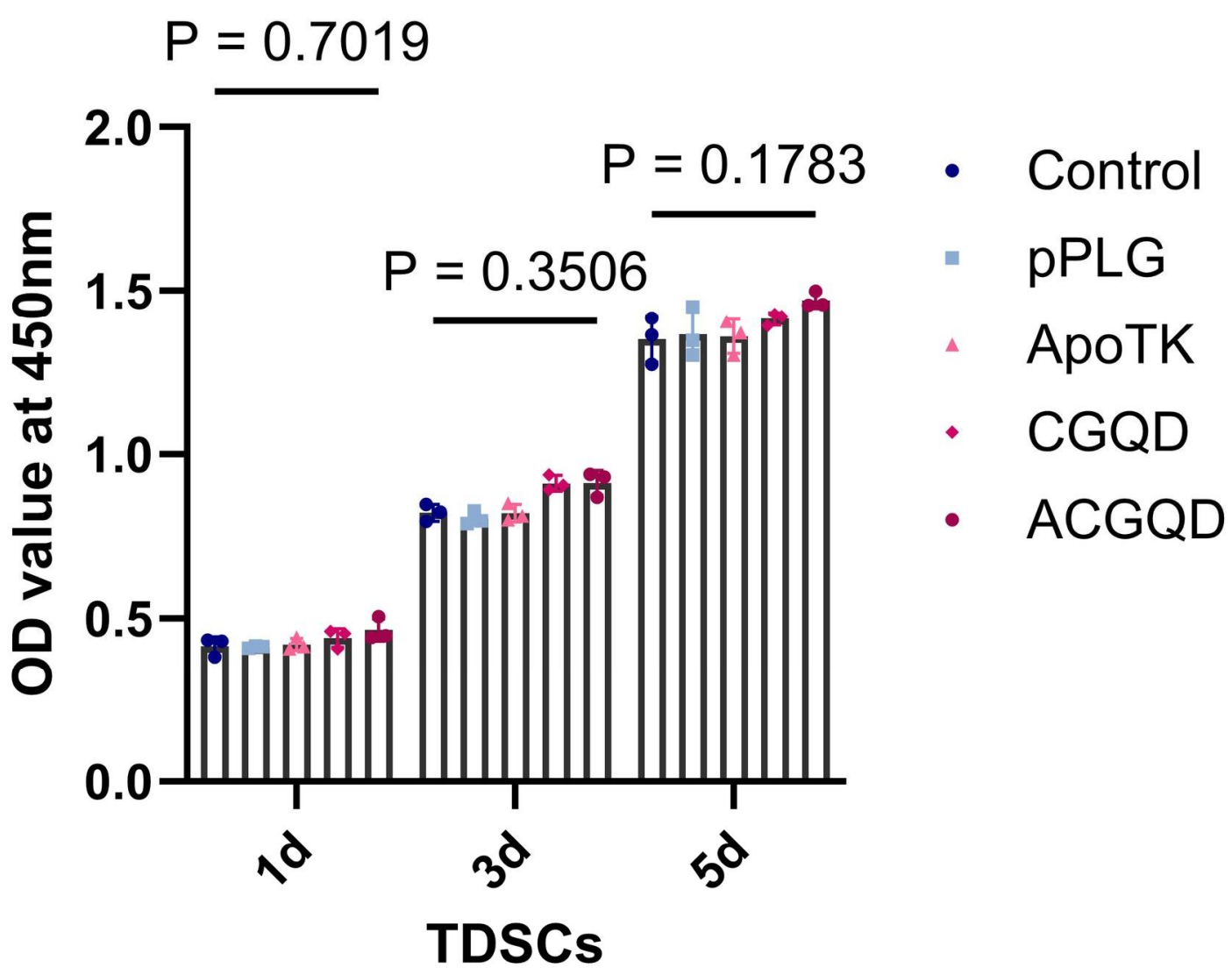

Figure S26. CCK-8 assessment of tendon-derived stem cells (TDSCs) exposed to the patch formulations. Absorbance at 450 nm was measured on days 1, 3, and 5 in the Control, pPLG, ApoTK, CGQD, and ACGQD groups. ApoTK, CGQD, and ACGQD label the corresponding cargo-loaded pPLG patches. Data are mean ± SD; *n* = 3 per group. Individual values are shown. *P* values for the indicated comparisons were calculated by one-way ANOVA with Tukey's post hoc test.

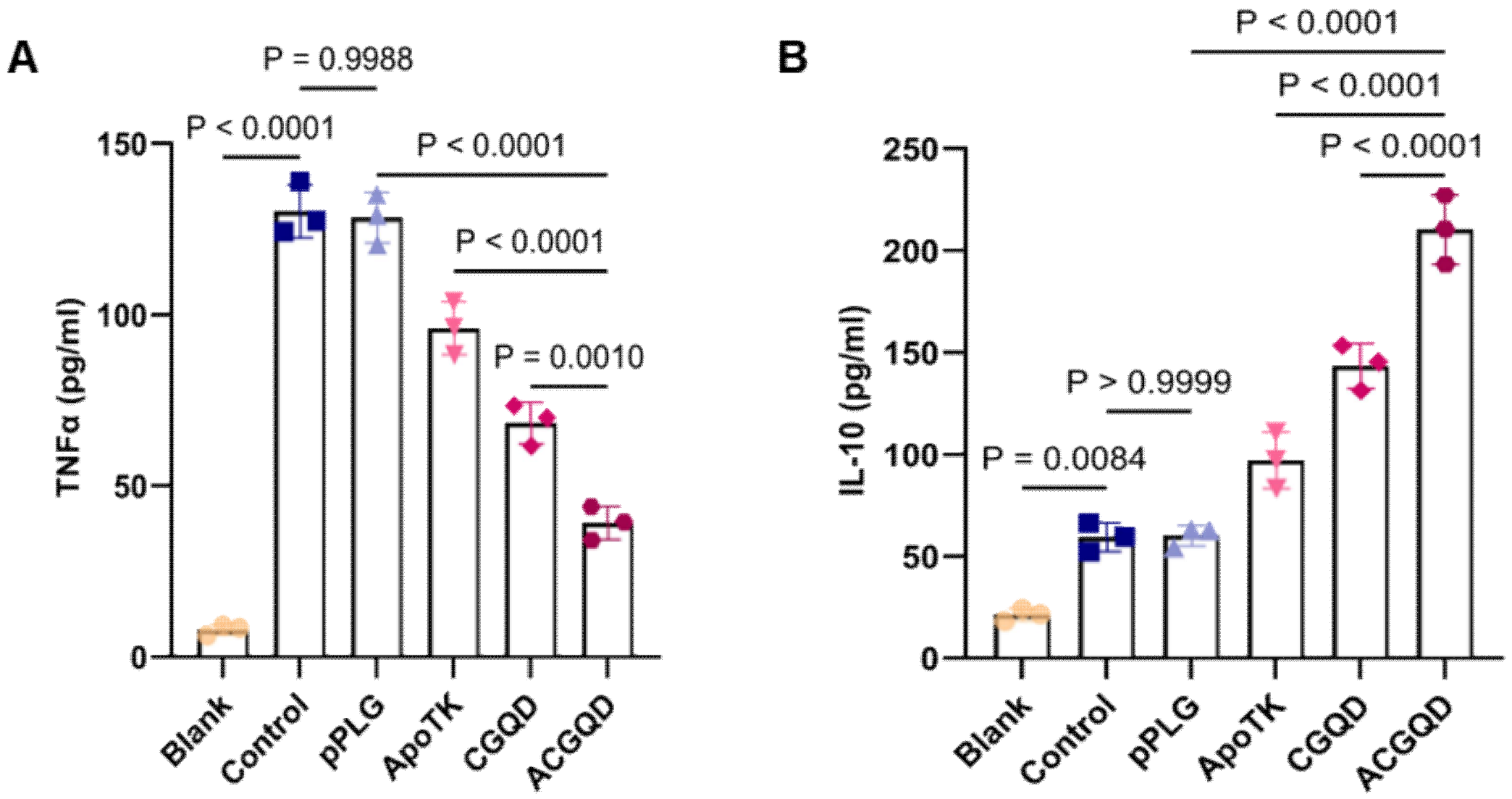

Figure S27. Cytokine secretion by macrophages exposed to the patch formulations. Culture-supernatant concentrations of (A) tumor necrosis factor-α (TNF-α) and (B) interleukin-10 (IL-10) were measured by enzyme-linked immunosorbent assay (ELISA) in the Blank, Control, pPLG, ApoTK, CGQD, and ACGQD groups. Blank denotes unstimulated macrophages, and Control denotes LPS-stimulated macrophages without a patch. ApoTK, CGQD, and ACGQD label the corresponding cargo-loaded pPLG patches. Concentrations are expressed in pg/mL. Data are mean ± SD; $n = 3$ per group. Individual values are shown. P values for the indicated comparisons were calculated by one-way ANOVA with Tukey's post hoc test.

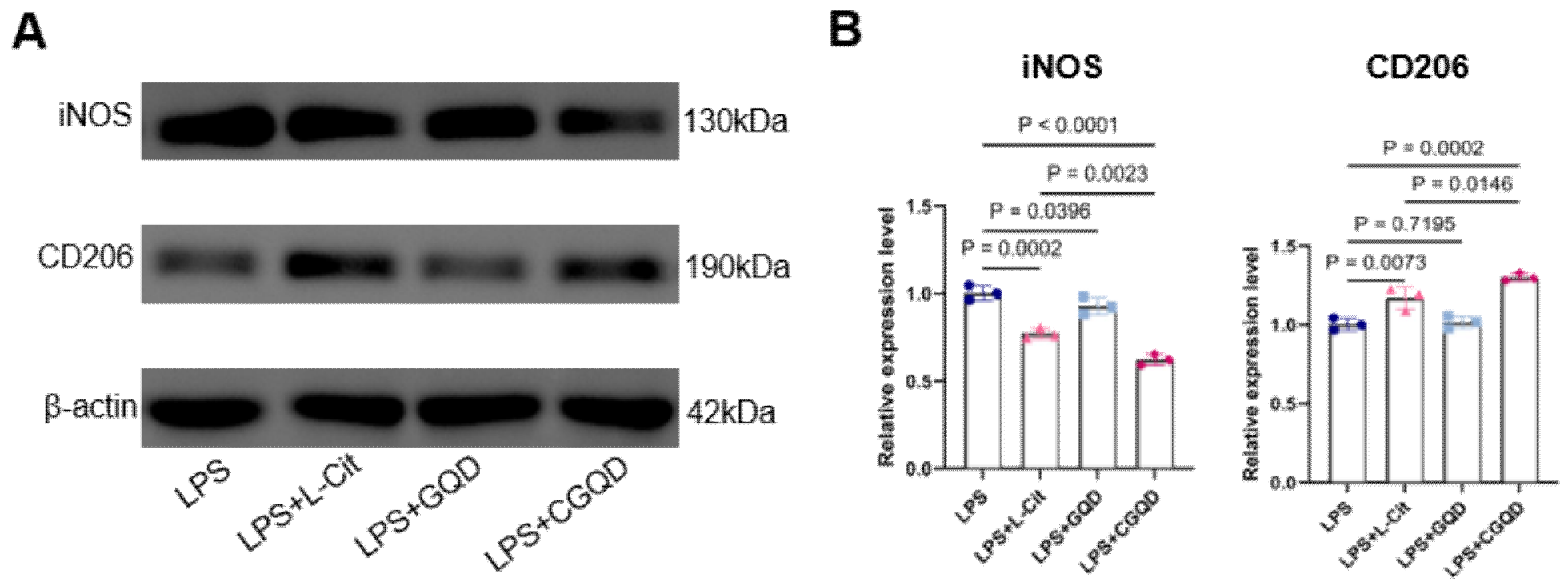

Figure S28. Comparison of free L-citrulline, GQD, and CGQD in LPS-stimulated macrophages. Cells were exposed for 24 h to free L-citrulline, GQD, or CGQD, each at 50 μg mL⁻¹, with LPS alone as the reference. The comparison used the same nominal total mass concentration of each test material rather than equimolar or equivalent-citrulline doses. (A) Representative immunoblots of inducible nitric oxide synthase (iNOS) and CD206 in the LPS, LPS + L-Cit, LPS + GQD, and LPS + CGQD groups; β-actin is the loading control. (B) Densitometric analysis of iNOS and CD206, normalized to β-actin and expressed relative to the LPS reference. L-Cit denotes free L-citrulline. Data are mean ± SD; $n = 3$ per group. P values for the indicated comparisons were calculated by one-way ANOVA with Tukey's post hoc test.

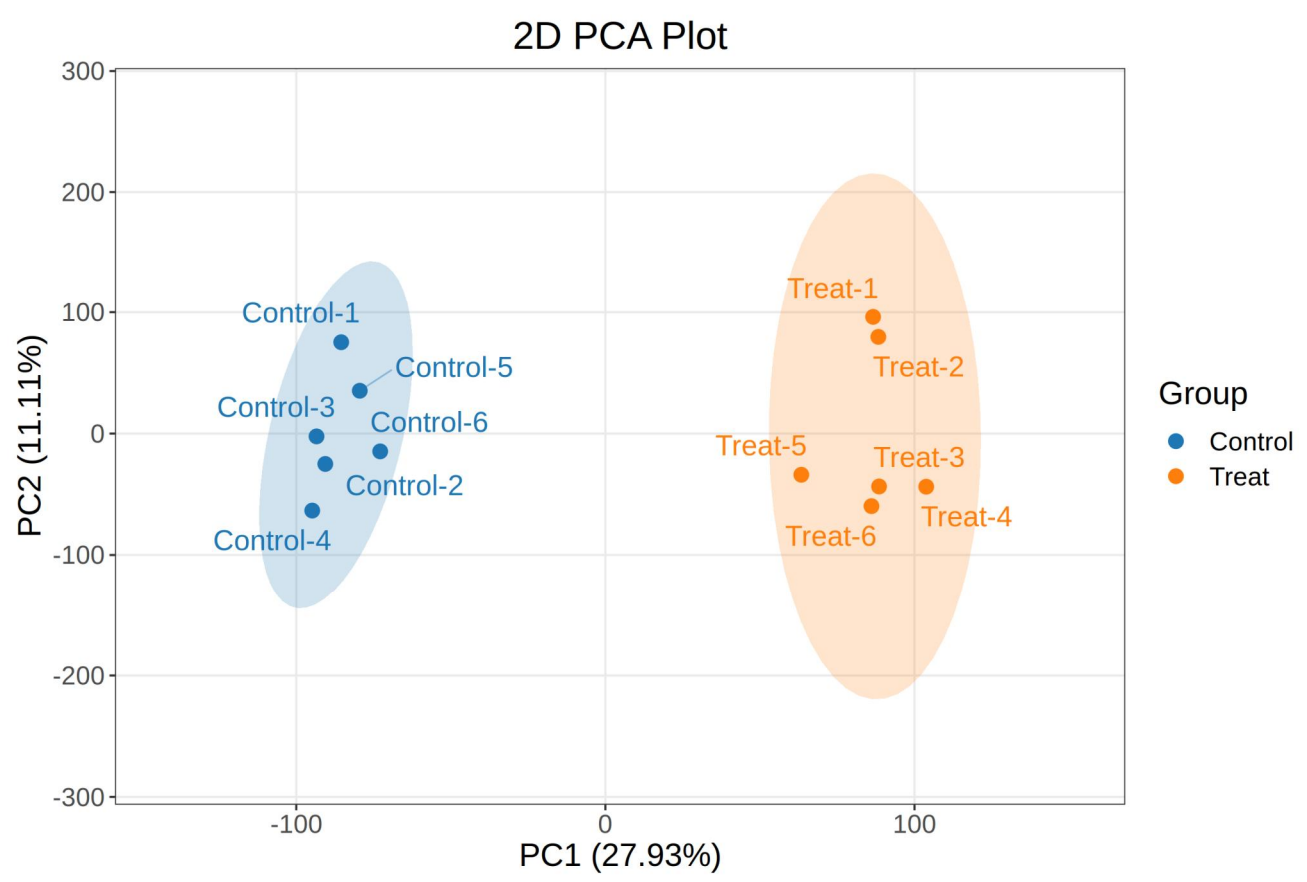

Figure S29. Principal component analysis (PCA) of bulk RNA-sequencing expression profiles. Control denotes LPS-stimulated macrophages, and Treat denotes LPS-stimulated macrophages exposed to ACGQD@pPLG. Each point represents one sample; n = 6 per group.

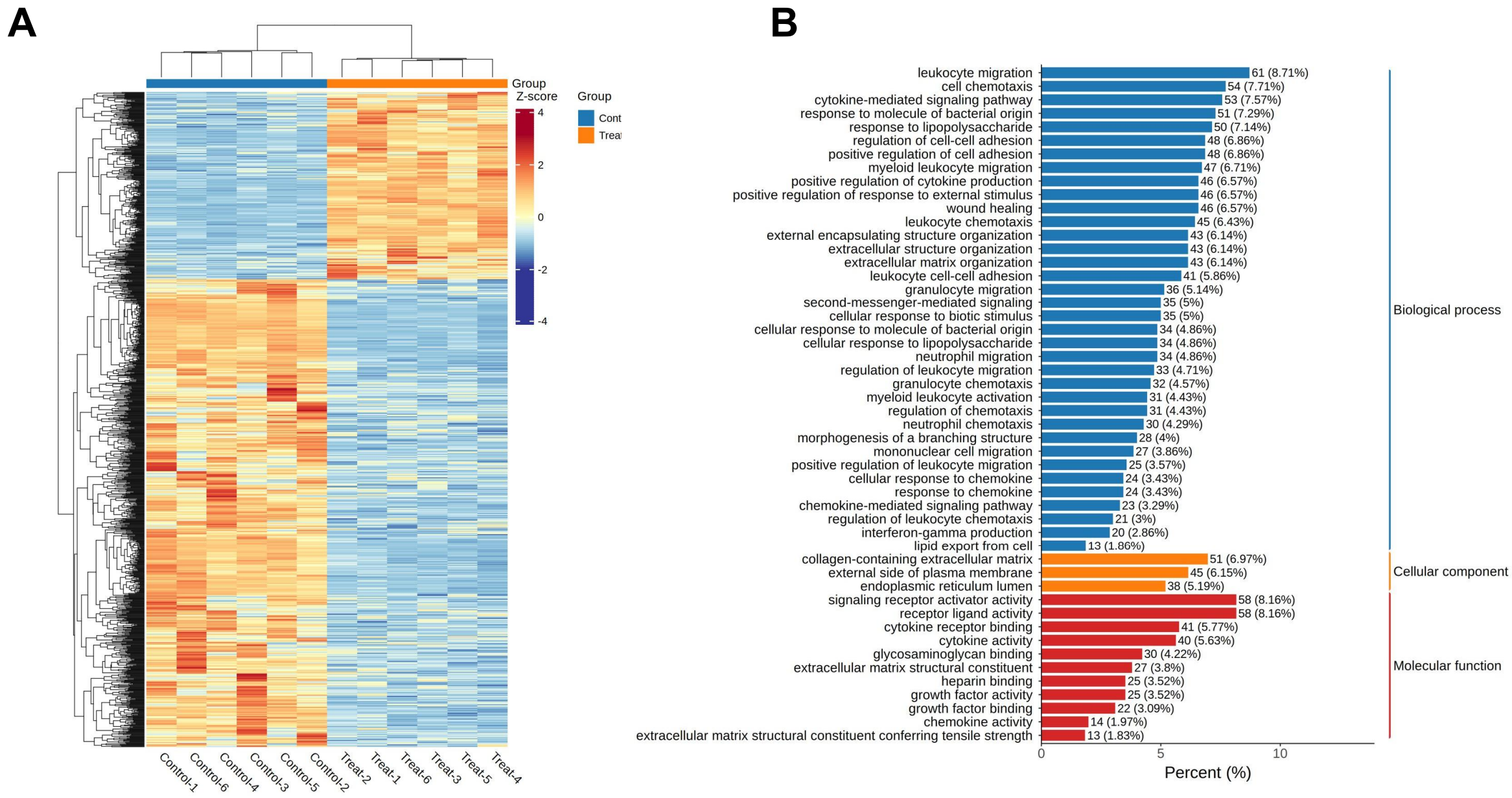

Figure S30. Differential gene expression and Gene Ontology (GO) annotation **in the Control and Treat groups.** (A) Hierarchical clustering heatmap of differentially expressed genes. Rows represent genes and columns represent the six samples in each group; colors indicate row-wise Z scores. (B) GO annotation of the differentially expressed genes, grouped into biological process (blue), cellular component (orange), and molecular function (red). Numbers beside the bars indicate gene counts and the corresponding percentages.

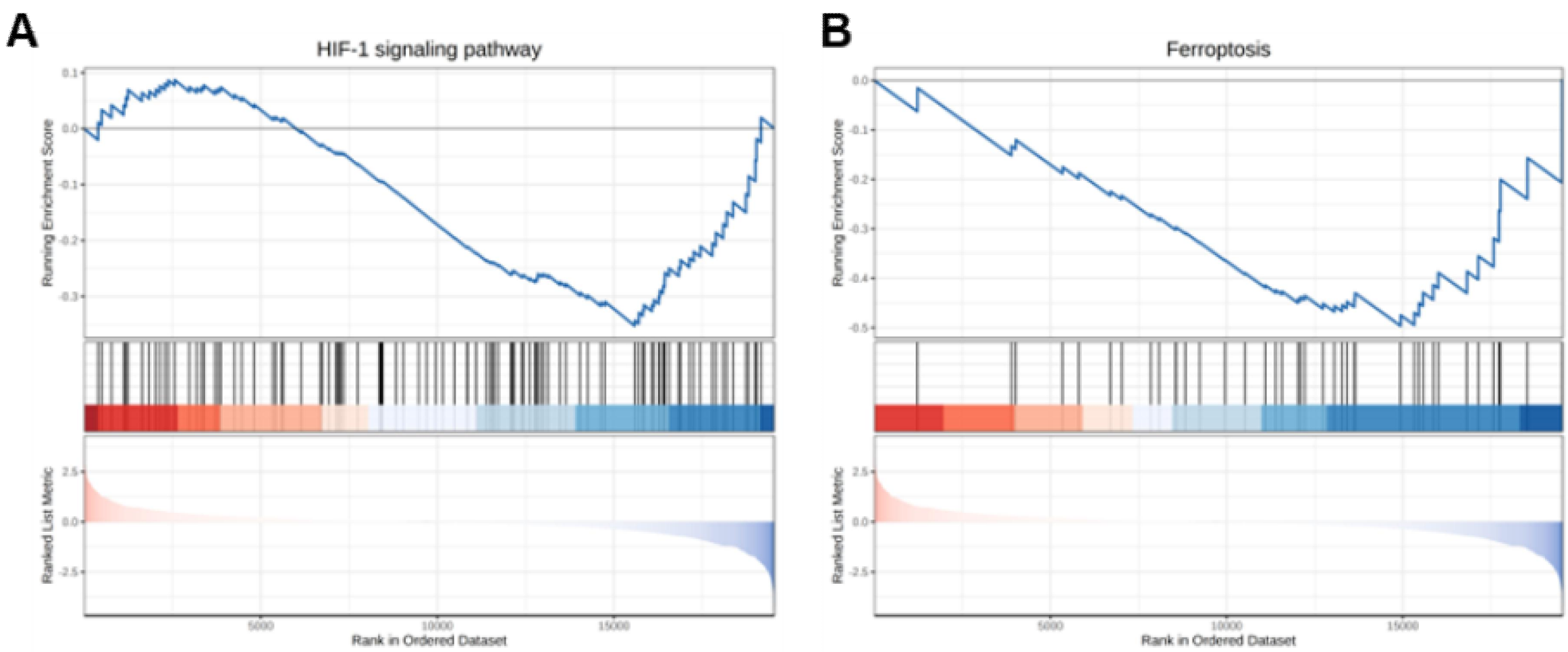

Figure S31. Gene set enrichment analysis (GSEA) of the HIF-1 signaling and ferroptosis pathways. (A) HIF-1 signaling pathway, normalized enrichment score (NES) = −1.117. (B) Ferroptosis pathway, NES = −1.376. In each panel, the upper plot shows the running enrichment score, vertical ticks mark pathway-gene positions in the ranked list, and the lower plot shows the ranking metric.

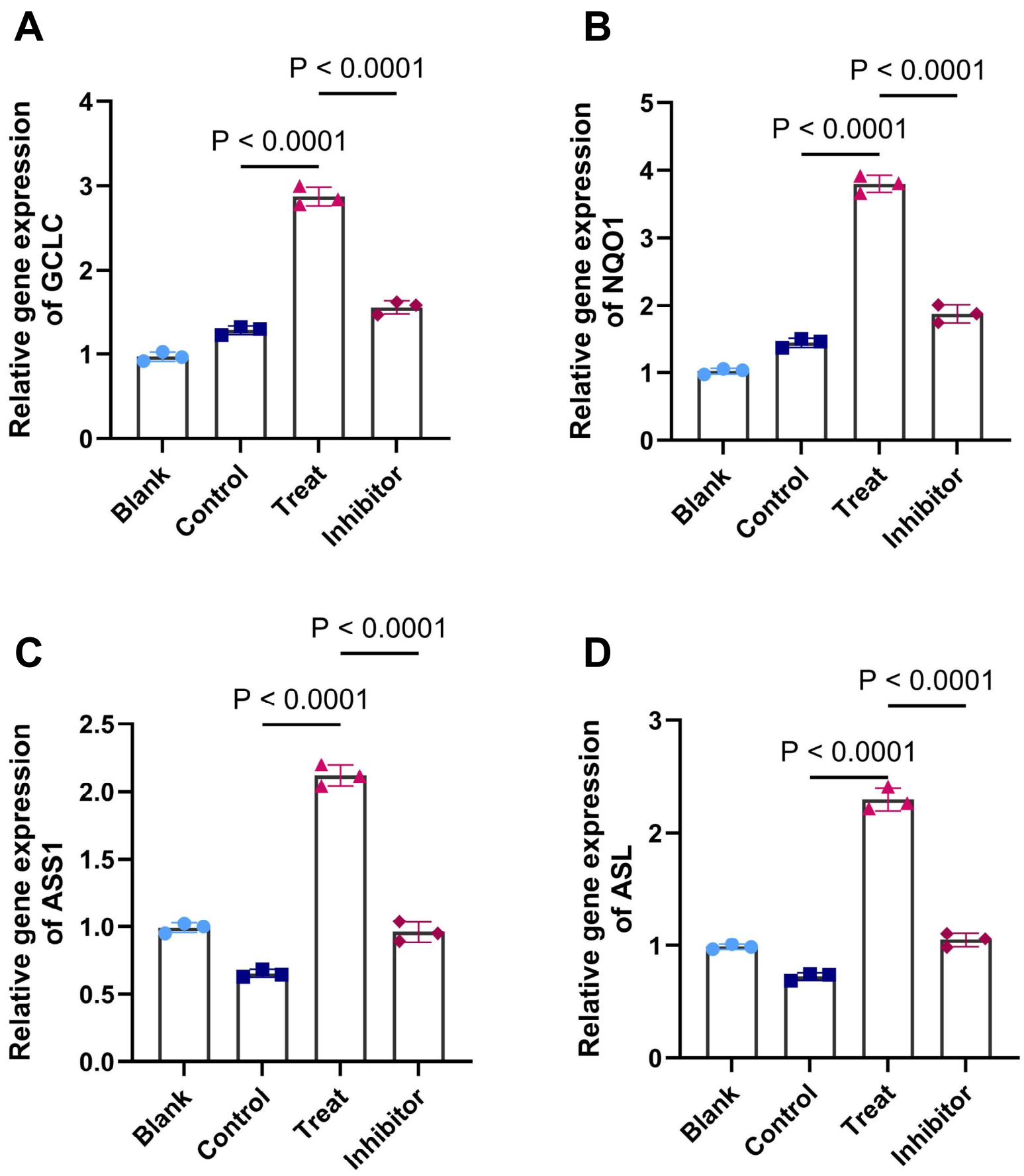

223 **Figure S32. RT-qPCR analysis of antioxidant-response and citrulline–arginine recycling genes after LY294002 treatment.** Relative mRNA expression of (A) *GCLC*, (B) *NQO1*, (C) *ASS1*, and (D) *ASL* in the Blank, Control (LPS), Treat (LPS + ACGQD@pPLG), and Inhibitor (LPS + ACGQD@pPLG + LY294002) groups. Data are mean ± SD with individual values shown; $n = 3$. P values for the indicated comparisons were calculated by one-way ANOVA with Tukey's post hoc test.

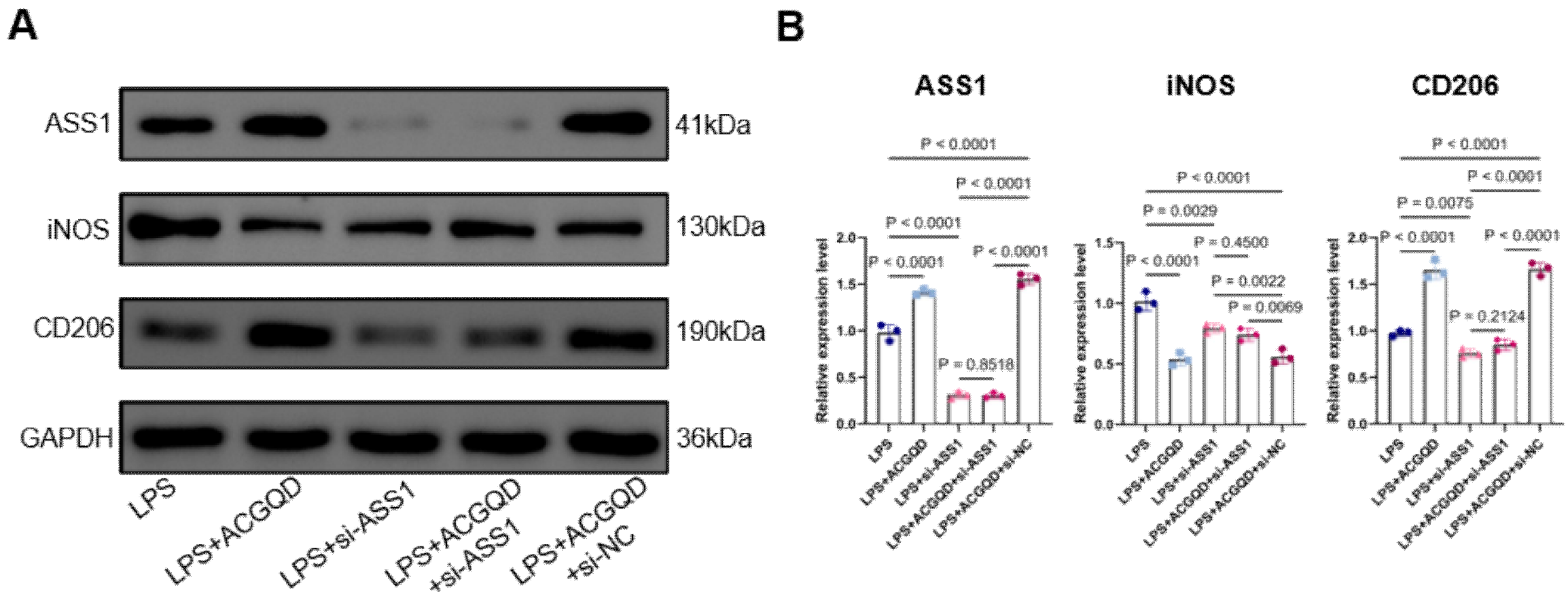

Figure S33. ASS1 knockdown in ACGQD-treated, LPS-stimulated macrophages. (A) Representative immunoblots of ASS1, iNOS, and CD206 in the LPS, LPS + ACGQD, LPS + si-ASS1, and LPS + ACGQD + si-ASS1 groups, together with an LPS + ACGQD + si-NC control group; GAPDH is the loading control. (B) Densitometric analysis of ASS1, iNOS, and CD206, normalized to GAPDH and expressed relative to the LPS reference. si-ASS1 denotes ASS1-targeting small interfering RNA and si-NC denotes the negative-control siRNA. Data are mean ± SD; $n = 3$ per group. *P* values for the indicated comparisons were calculated by one-way ANOVA with Tukey's post hoc test.

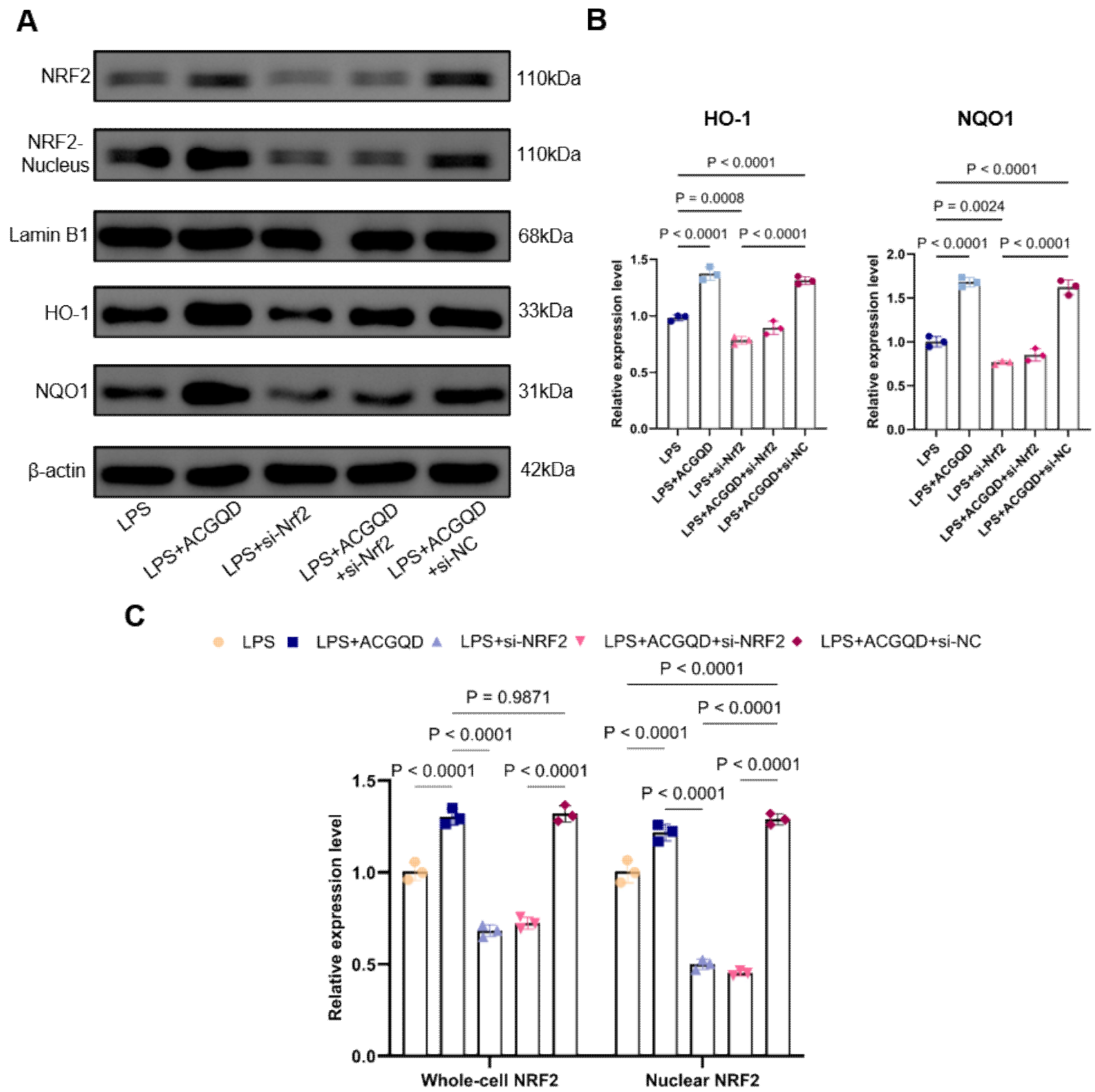
A
NRF2
NRF2-Nucleus
Lamin B1
HO-1
NQO1
β-actin
110kDa
110kDa
68kDa
33kDa
31kDa
42kDa
LPS
LPS+ACGQD
LPS+si-Nrf2
LPS+ACGQD +si-Nrf2
LPS+ACGQD +si-NC
B
HO-1
NQO1
Relative expression level
P < 0.0001
P = 0.0008
P = 0.0024
C
LPS
LPS+ACGQD
LPS+si-NRF2
LPS+ACGQD+si-NRF2
LPS+ACGQD+si-NC
P = 0.9871
P < 0.0001
Whole-cell NRF2
Nuclear NRF2

238

Figure S34. NRF2 knockdown in ACGQD-treated, LPS-stimulated macrophages. (A) Representative immunoblots of whole-cell NRF2, nuclear NRF2, heme oxygenase-1 (HO-1), and NAD(P)H quinone oxidoreductase 1 (NQO1) in the LPS, LPS + ACGQD, LPS + si-NRF2, and LPS + ACGQD + si-NRF2 groups, together with an LPS + ACGQD + si-NC control group. β-actin and Lamin B1 are the loading controls for whole-cell and nuclear proteins, respectively. (B) Densitometric analysis of HO-1 and NQO1. (C) Densitometric analysis of whole-cell and nuclear NRF2. Whole-cell NRF2, HO-1, and NQO1 were normalized to β-actin, and nuclear NRF2 was normalized to Lamin B1; values are expressed relative to the LPS reference. si-NRF2 denotes siRNA targeting *NFE2L2*, the gene encoding NRF2, and si-NC denotes negative-control siRNA. Data are mean ± SD; $n = 3$ per group. P values for the indicated comparisons were calculated by one-way ANOVA with Tukey's post hoc test.

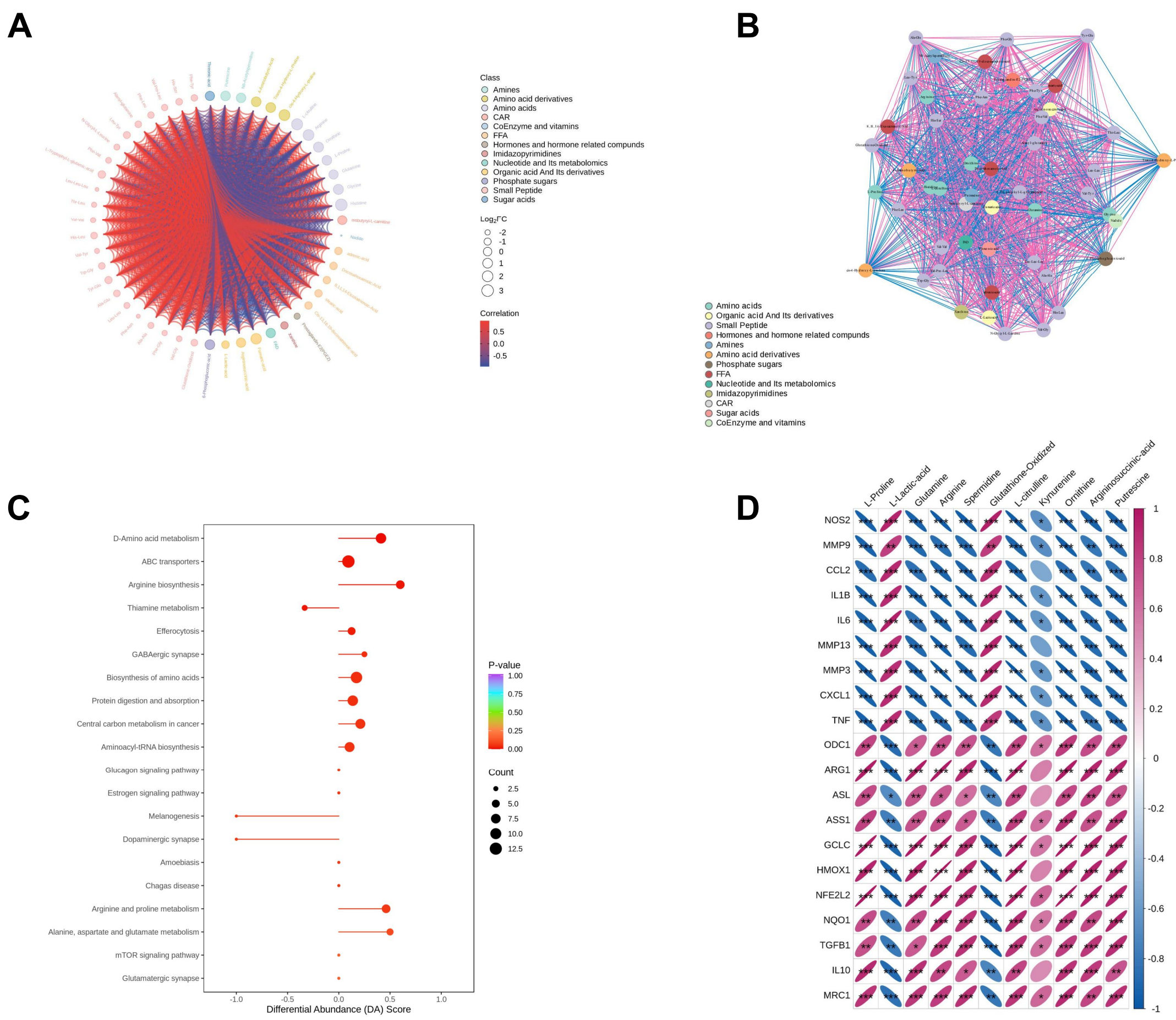

Figure S35. Metabolite correlation networks and gene–metabolite associations. (A) Circular correlation plot of differential metabolites; node colors denote metabolite classes, node sizes encode $\log_2$ fold changes, and edge colors indicate Pearson correlation coefficients. (B) Metabolite correlation network; node colors denote metabolite classes, with pink and blue edges representing positive and negative correlations, respectively. (C) Differential abundance (DA) scores for KEGG pathways; point sizes indicate metabolite counts and colors indicate P values. (D) Pearson correlation matrix between the indicated genes (rows) and metabolites (columns); ellipse color and orientation indicate the sign and magnitude of each correlation. *$P < 0.05$, **$P < 0.01$, and ***$P < 0.001$; $n = 6$.

A

Name	Distance	Categroy	Type
X:ARG415:NH1 - lig:O80	2.88556	Hydrogen Bond	Conventional Hydrogen Bond
X:SER555:OG - lig:O104	2.73931	Hydrogen Bond	Conventional Hydrogen Bond
lig:H55 - X:SER383:O	2.65591	Hydrogen Bond	Conventional Hydrogen Bond
lig:H59 - X:TYR334:OH	2.21792	Hydrogen Bond	Conventional Hydrogen Bond
lig:H65 - X:TYR334:OH	2.14243	Hydrogen Bond	Conventional Hydrogen Bond
lig:S43 - X:TYR572	4.44585	Other	Pi-Sulfur
lig:S43 - X:PHE577	4.91399	Other	Pi-Sulfur

B

Name	Distance	Categroy	Type
X:TYR334:OH - lig:O135	3.28018	Hydrogen Bond	Conventional Hydrogen Bond
X:ARG380:NH1 - lig:O95	2.82964	Hydrogen Bond	Conventional Hydrogen Bond
X:SER555:OG - lig:O100	3.2585	Hydrogen Bond	Conventional Hydrogen Bond
X:SER602:OG - lig:O106	2.70069	Hydrogen Bond	Conventional Hydrogen Bond
X:GLY364:CA - lig:O111	3.77042	Hydrogen Bond	Carbon Hydrogen Bond
X:GLY603:CA - lig:O110	3.50498	Hydrogen Bond	Carbon Hydrogen Bond
lig:C118 - X:TYR334	3.48981	Hydrophobic	Pi-Sigma

C

Name	Distance	Categroy	Type
lig:H14 - B:TYR373:OH	2.48021	Hydrogen Bond	Conventional Hydrogen Bond
lig:H17 - B:PRO350:O	2.41901	Hydrogen Bond	Conventional Hydrogen Bond
lig:H18 - B:TRP372:O	1.98872	Hydrogen Bond	Conventional Hydrogen Bond

D

Name	Distance	Categroy	Type
lig:H2 - B:TRP372:O	2.45224	Hydrogen Bond	Conventional Hydrogen Bond
lig:H17 - B:TYR347:OH	3.03122	Hydrogen Bond	Conventional Hydrogen Bond
lig:H17 - B:TYR373:OH	2.41412	Hydrogen Bond	Conventional Hydrogen Bond
lig:H18 - B:TYR373:OH	2.7339	Hydrogen Bond	Conventional Hydrogen Bond
lig:H19 - B:GLU377:OE1	2.42654	Hydrogen Bond	Conventional Hydrogen Bond

Figure S36. Predicted noncovalent contacts in the docking models. Contact lists are shown for (A) the CGQD model–KEAP1, (B) NRF2–KEAP1, (C) citrulline–iNOS, and (D) arginine–iNOS complexes. The interacting atoms or residues, distances (Å), interaction categories, and interaction types are listed. These contacts describe the selected modeled poses.

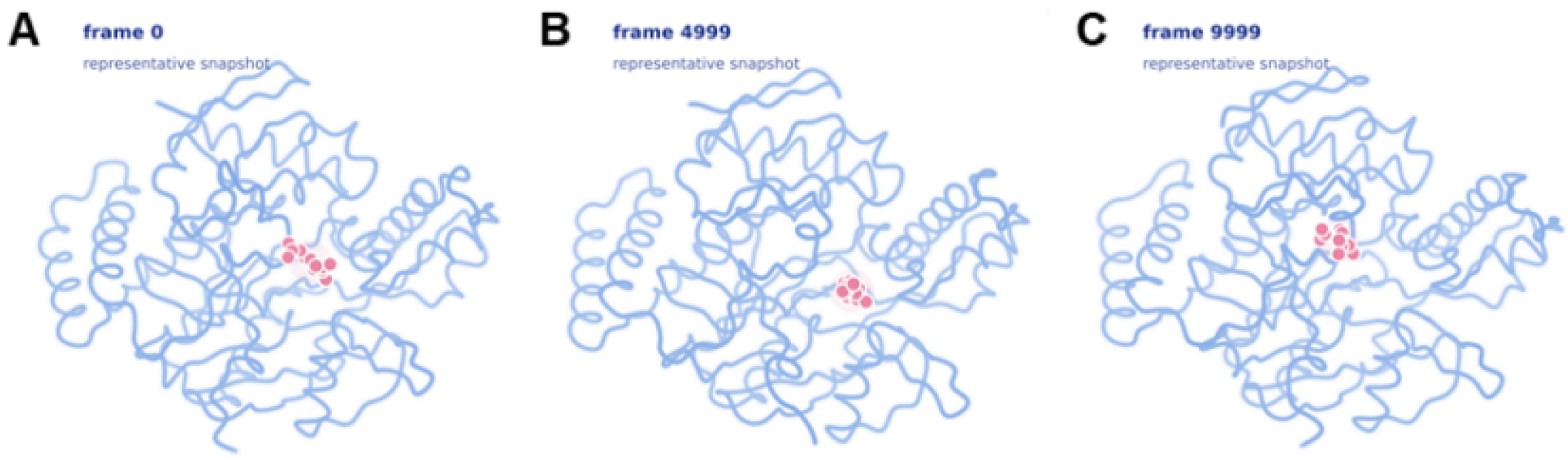

Figure S37. Selected snapshots of the citrulline–iNOS molecular dynamics trajectory. (A–C) Frames 0, 4999, and 9999, respectively. iNOS is shown as a light-blue cartoon and citrulline as pink spheres.

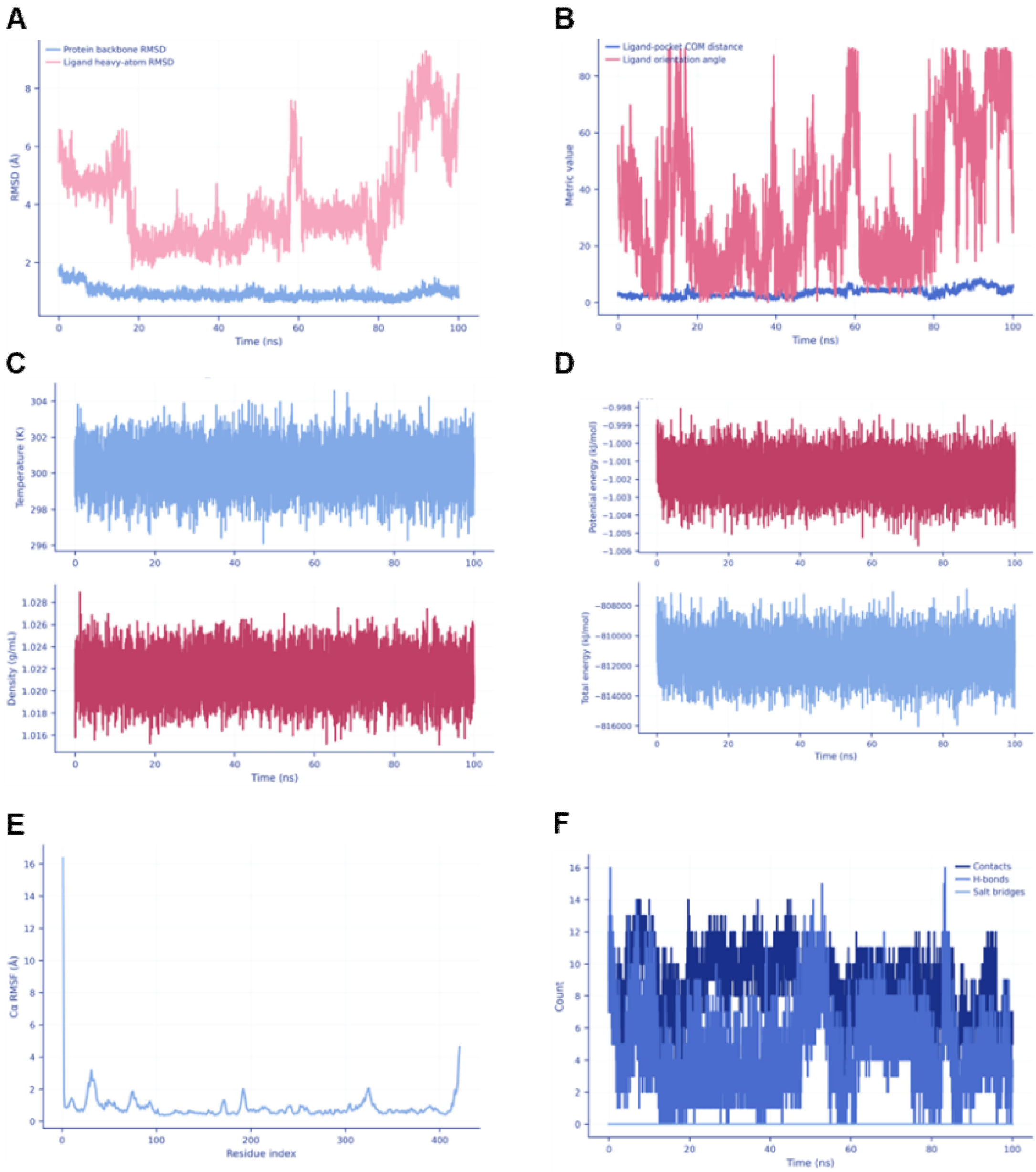

Figure S38. Molecular dynamics descriptors of the citrulline–iNOS complex over 100 ns. (A) Root-mean-square deviation (RMSD) of the protein backbone and ligand heavy atoms. (B) Ligand–pocket center-of-mass distance and ligand orientation angle. (C) System temperature and density. (D) Potential energy and total energy. (E) Per-residue Cα root-mean-square fluctuation (RMSF). (F) Numbers of protein–ligand contacts, hydrogen bonds, and salt bridges over time.

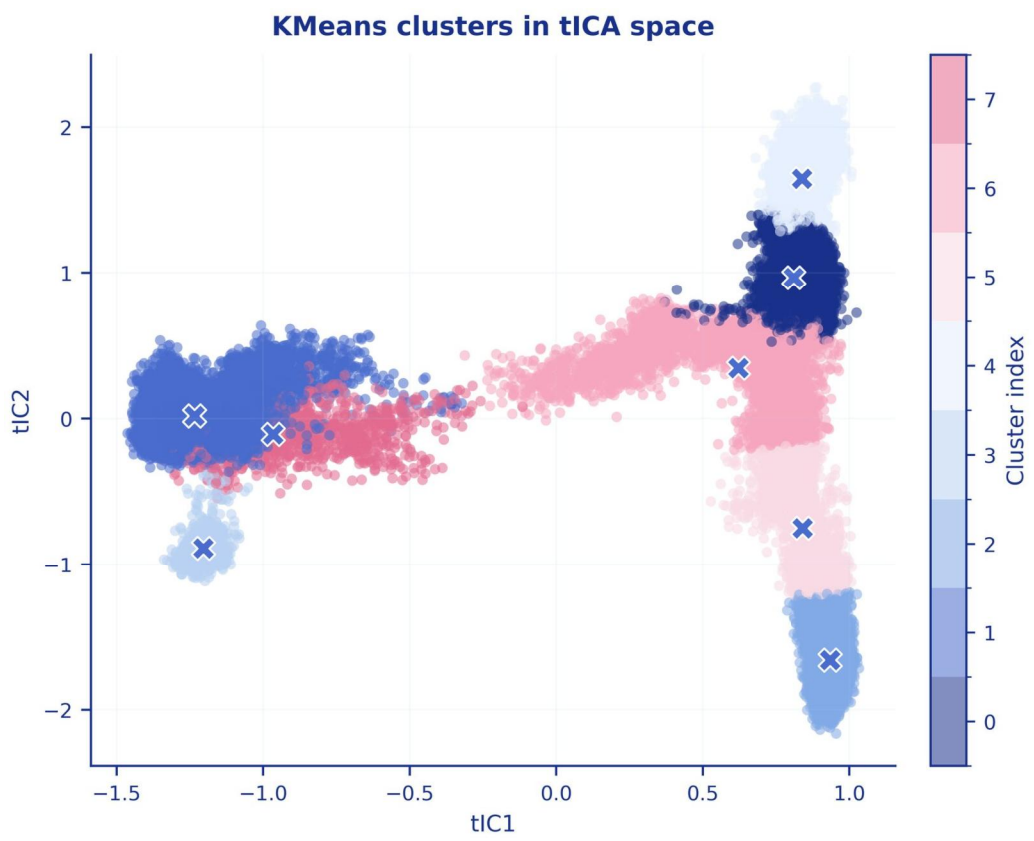

Figure S39. k-Means clustering of the citrulline–iNOS molecular dynamics trajectory in time-lagged independent component analysis (tICA) space. The trajectory is projected onto the first two components, tIC1 and tIC2. Point colors identify the eight clusters (indices 0–7); crosses indicate cluster centers.

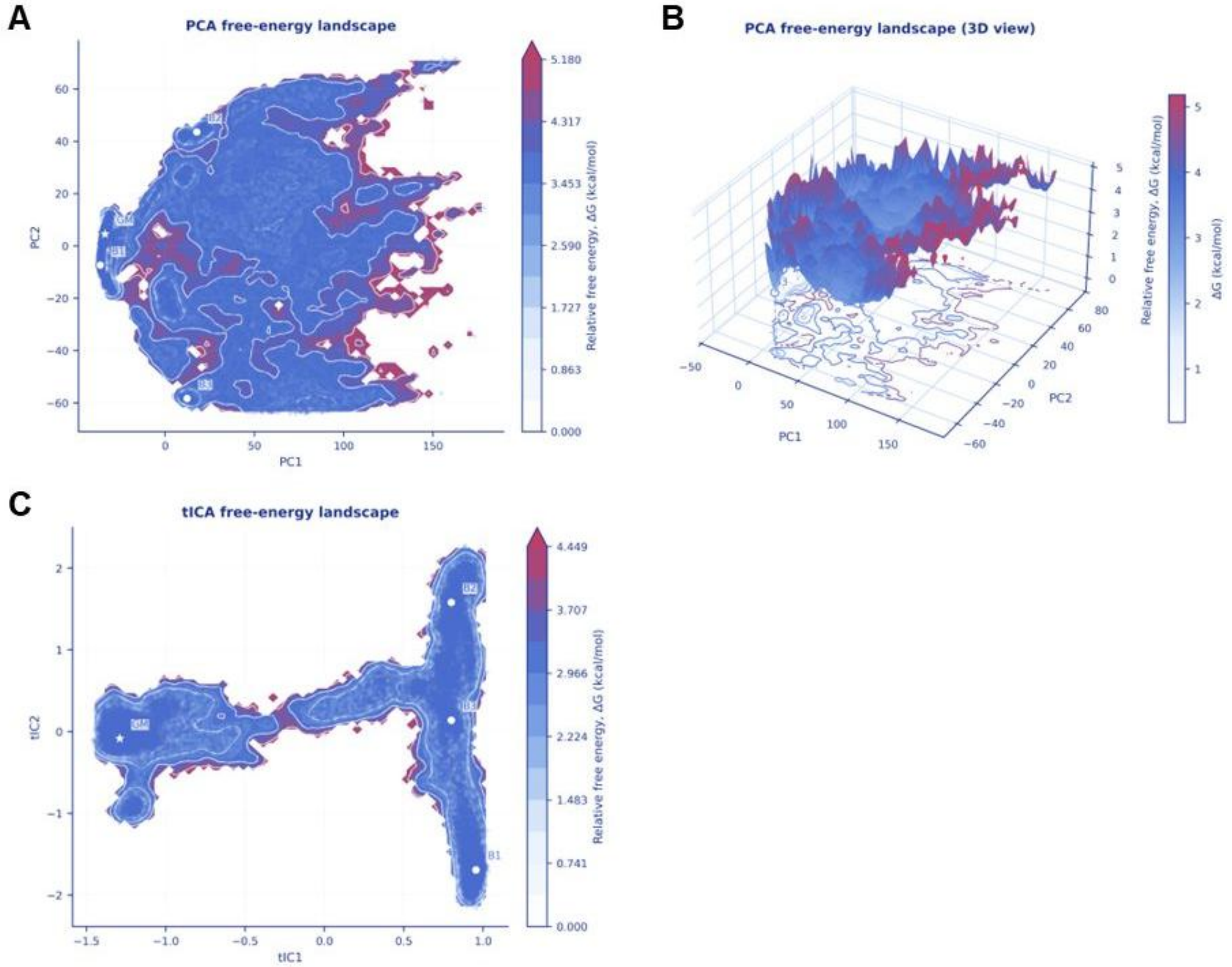

Figure S40. Projected free-energy landscapes of the citrulline–iNOS molecular dynamics trajectory. (A) Two-dimensional landscape in the first two principal components (PC1 and PC2). (B) Three-dimensional view of the same projection. (C) Landscape in the first two time-lagged independent components (tIC1 and tIC2). Color scales indicate relative free energy (kcal/mol). Stars mark the labeled global minima (GM), and circles mark the labeled additional basins B1–B3.

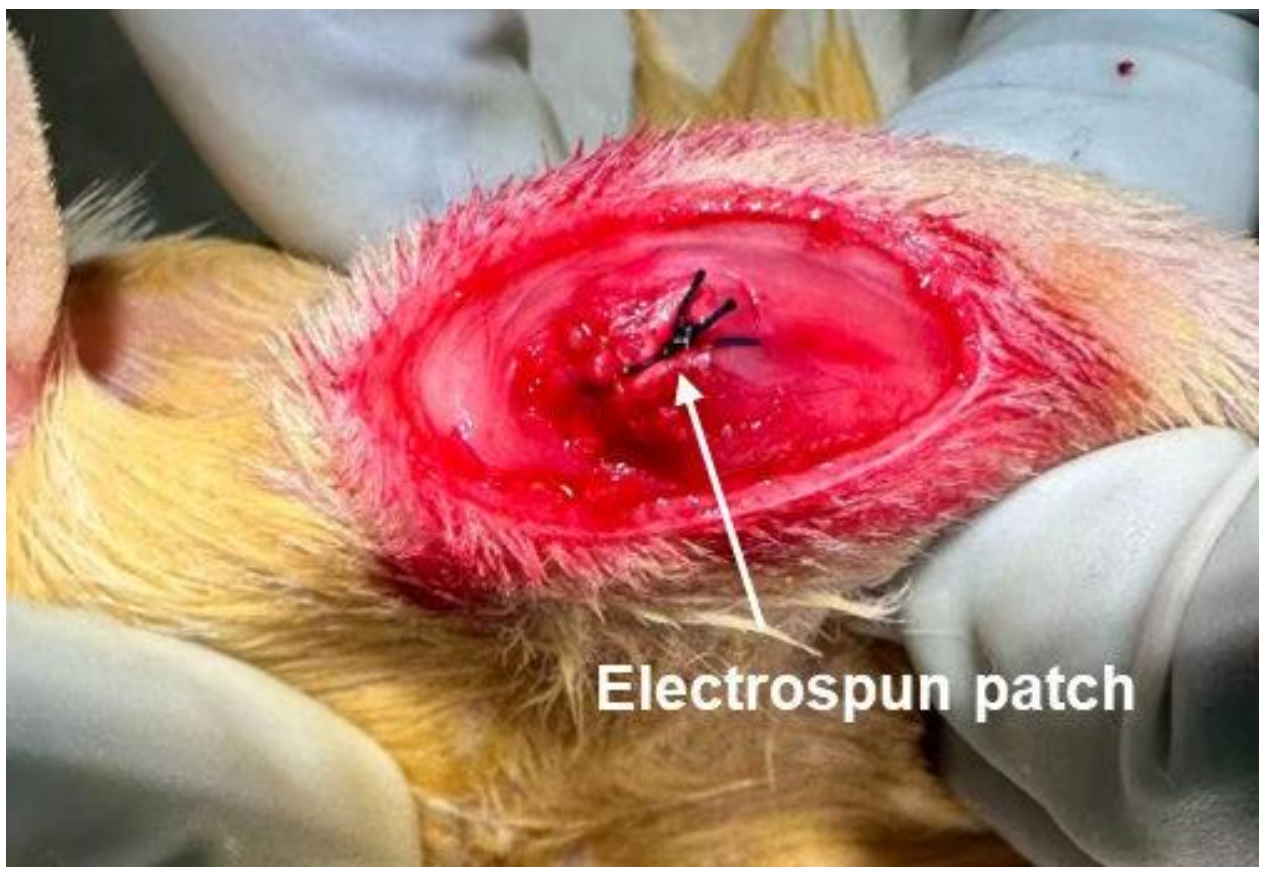

Figure S41. Intraoperative photograph of electrospun patch placement in the rat rotator cuff repair model. The arrow indicates the patch at the repaired tendon-to-bone interface.

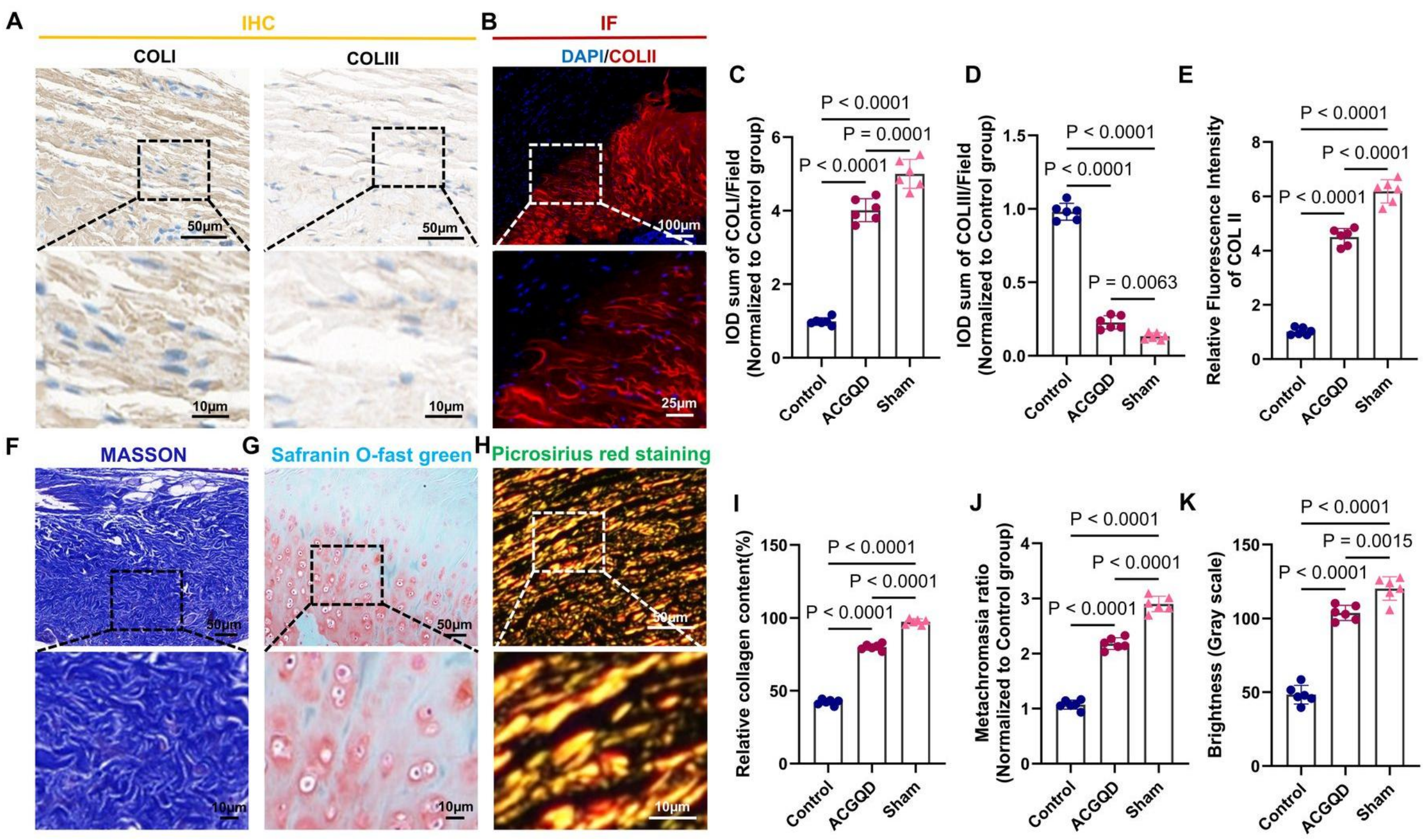

Figure S42. Histological reference from unoperated Sham rats and comparison with repair groups at 8 weeks. (A) Representative immunohistochemical staining for collagen I (COL I; left) and collagen III (COL III; right) in the Sham group. (B) Representative collagen II (COL II) immunofluorescence in the Sham group; COL II is shown in red and DAPI-stained nuclei in blue. (C, D) Integrated optical density (IOD) of COL I and COL III staining, respectively. (E) Relative COL II fluorescence intensity. (F–H) Representative Sham sections stained with Masson trichrome (F), Safranin O–fast green (G), and picrosirius red viewed under polarized light (H). (I–K) Quantification of Masson-based collagen content (I), relative Safranin O staining (J), and grayscale intensity of collagen birefringence in picrosirius red images (K). Dashed boxes mark the regions enlarged below. Scale bars: 50 μm in the overview images and 10 μm in the enlargements in A and F–H; 100 μm and 25 μm, respectively, in B. Quantitative panels compare Control, ACGQD@pPLG (labeled ACGQD), and unoperated Sham groups. COL I/COL III IOD and Safranin O staining are expressed relative to the Control reference, as indicated in the panels. Data are mean ± SD; *n* = 6 independent animals per group. *P* values for the indicated comparisons were calculated by one-way ANOVA with Tukey's post hoc test.

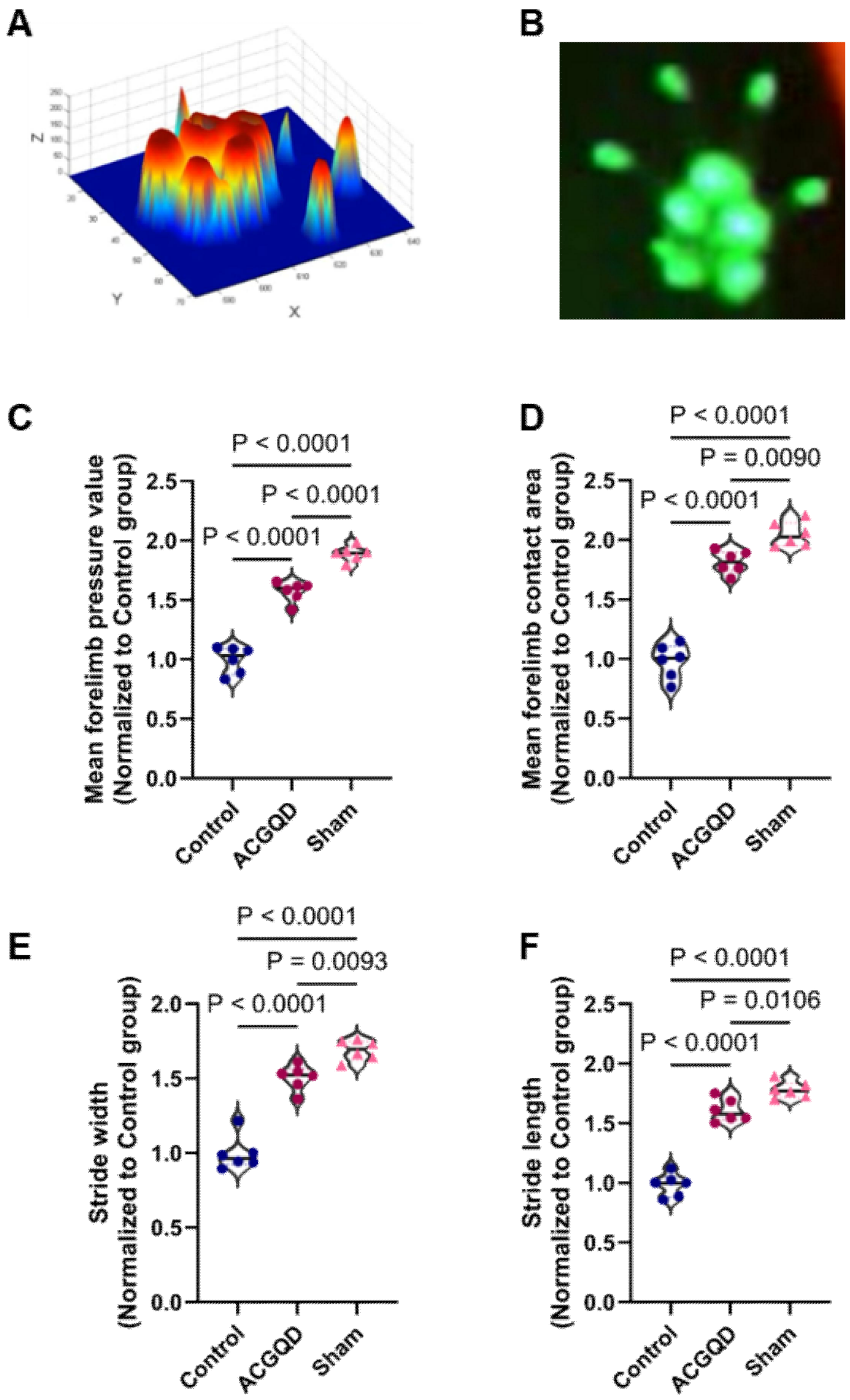

Figure S43. Gait reference from unoperated Sham rats and comparison with repair groups at 8 weeks. (A) Representative three-dimensional paw-contact pressure map from the Sham group. (B) Corresponding representative paw-contact image. (C–F) Mean forelimb contact pressure (C), mean forelimb contact area (D), stride width (E), and stride length (F) in the Control, ACGQD@pPLG (labeled ACGQD), and unoperated Sham groups. Each parameter is normalized to the repair-only Control reference. Data are mean ± SD; *n* = 6 independent animals per group. *P* values for the indicated comparisons were calculated by one-way ANOVA with Tukey's post hoc test.

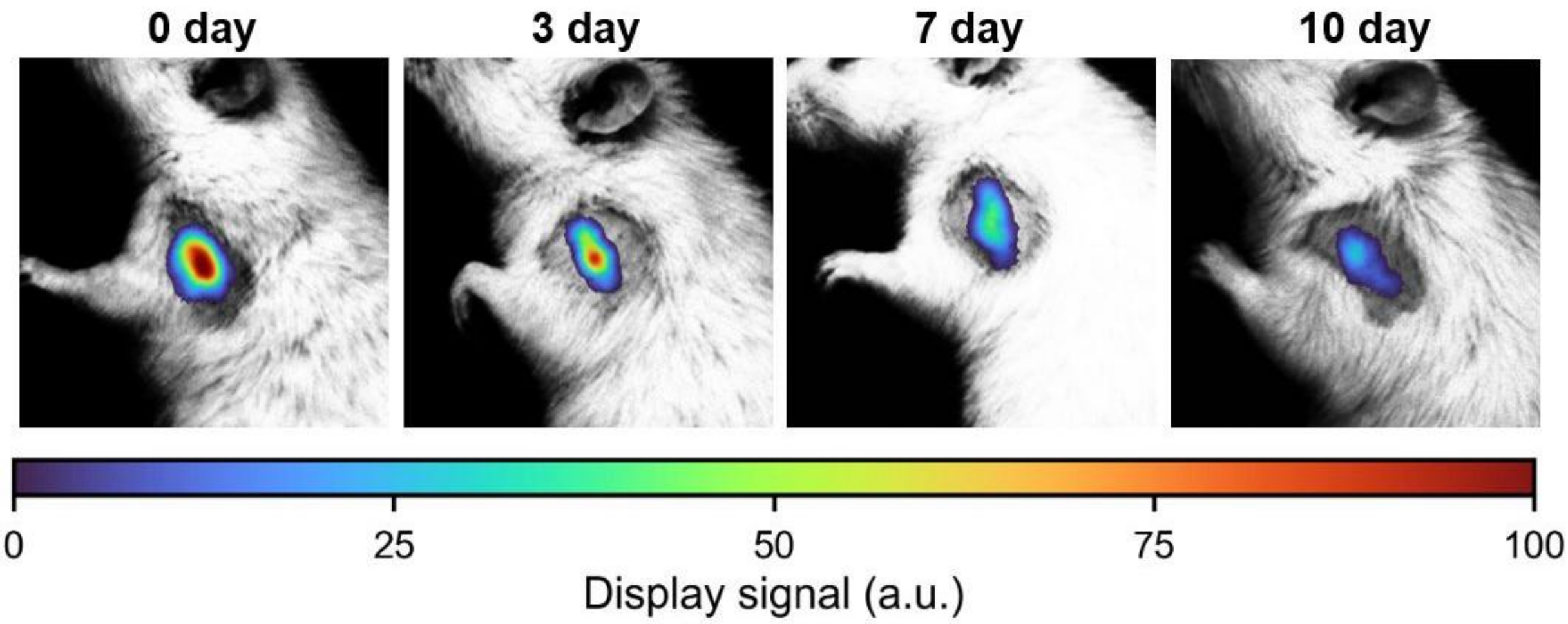

Figure S44. Longitudinal in vivo fluorescence imaging after local placement of a patch loaded with fluorescently labeled nanoparticles in rats. Representative images of the shoulder region acquired on days 0, 3, 7, and 10 after implantation (left to right) using identical acquisition settings and a common display range. The signal decreased over time but remained detectable at day 10.

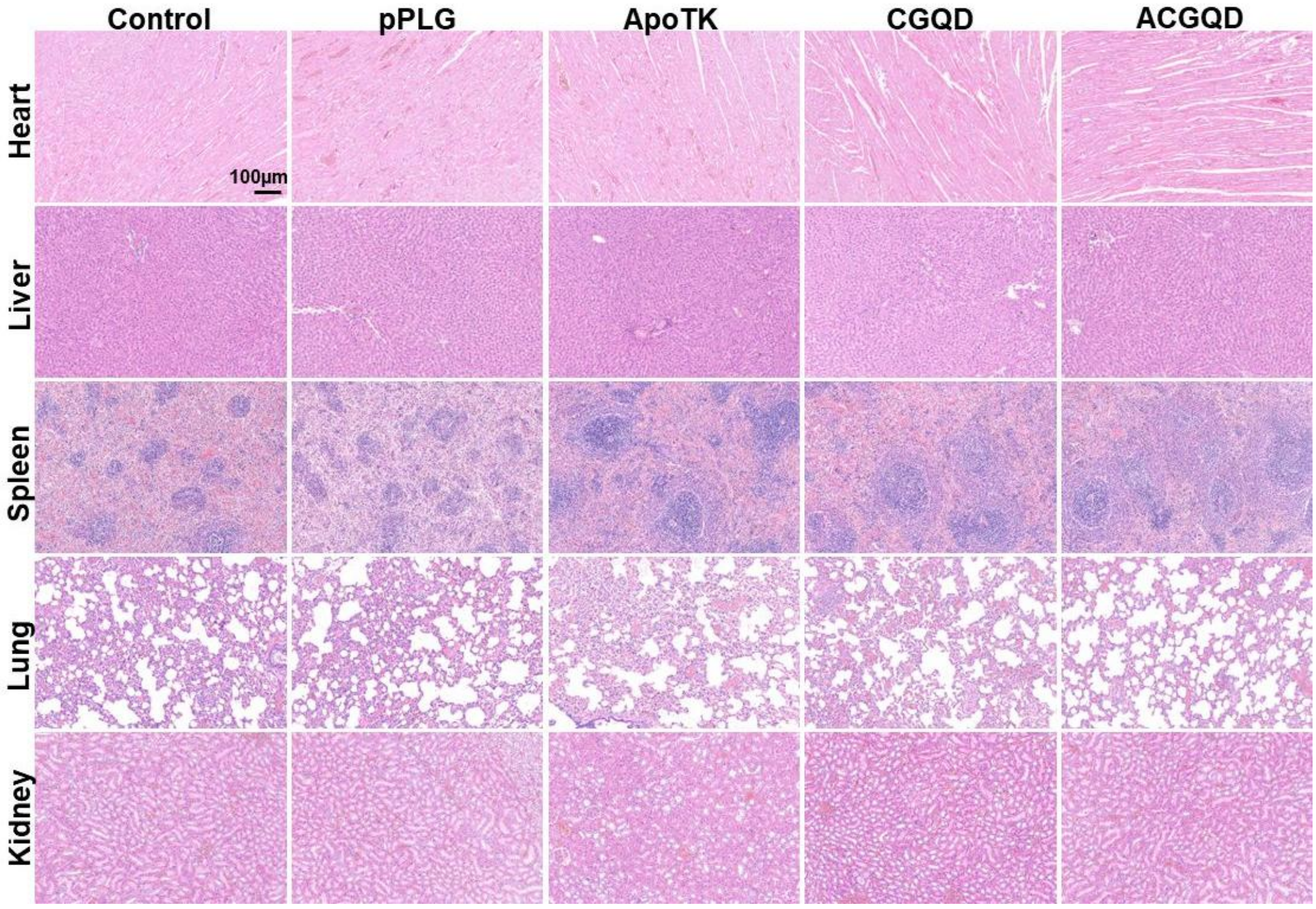

Figure S45. Major-organ histology at 8 weeks after rat rotator cuff repair. Representative hematoxylin and eosin (H&E)-stained sections of the heart, liver, spleen, lung, and kidney in the Control, pPLG, ApoTK, CGQD, and ACGQD groups. Control denotes repair alone; ApoTK, CGQD, and ACGQD label the corresponding cargo-loaded pPLG patches. Scale bar, 100 μm; *n* = 6 animals per group.

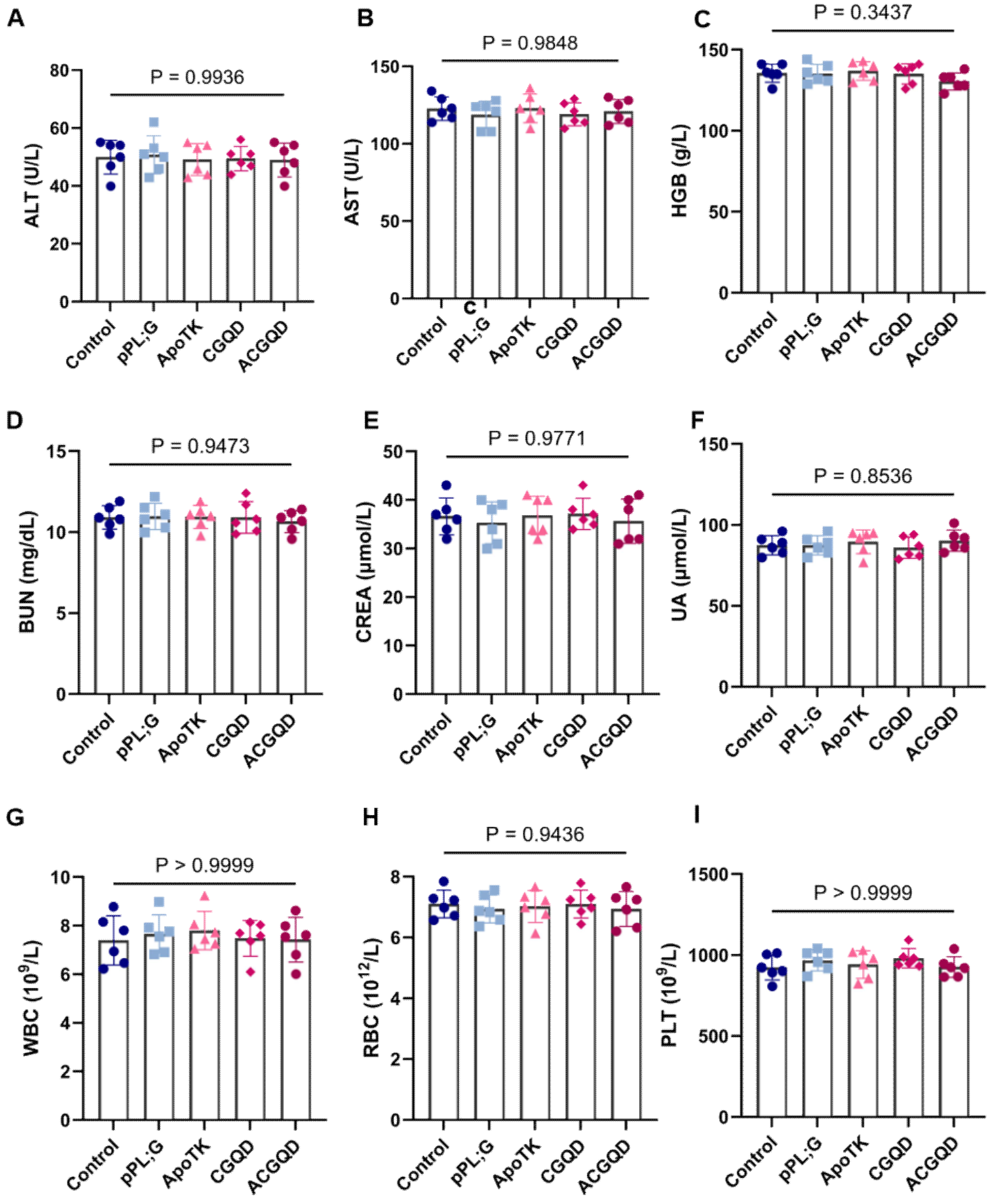
A
ALT (U/L)
P = 0.9936
B
AST (U/L)
P = 0.9848
C
HGB (g/L)
P = 0.3437
D
BUN (mg/dL)
P = 0.9473
E
CREA (μmol/L)
P = 0.9771
F
UA (μmol/L)
P = 0.8536
G
WBC (10^9/L)
P > 0.9999
H
RBC (10^{12}/L)
P = 0.9436
I
PLT (10^9/L)
P > 0.9999
Control
pPL;G
ApoTK
CGQD
ACGQD

328

Figure S46. Serum biochemistry and hematological measurements at 8 weeks after rat rotator cuff repair. (A) Alanine aminotransferase (ALT). (B) Aspartate aminotransferase (AST). (C) Hemoglobin (HGB). (D) Blood urea nitrogen (BUN). (E) Creatinine (CREA). (F) Uric acid (UA). (G) White blood cell count (WBC). (H) Red blood cell count (RBC). (I) Platelet count (PLT). The Control, pPLG, ApoTK, CGQD, and ACGQD groups are compared. Control denotes repair alone; ApoTK, CGQD, and ACGQD label the corresponding cargo-loaded pPLG patches. Data are mean ± SD; $n = 6$ animals per group. P values from one-way ANOVA are shown.

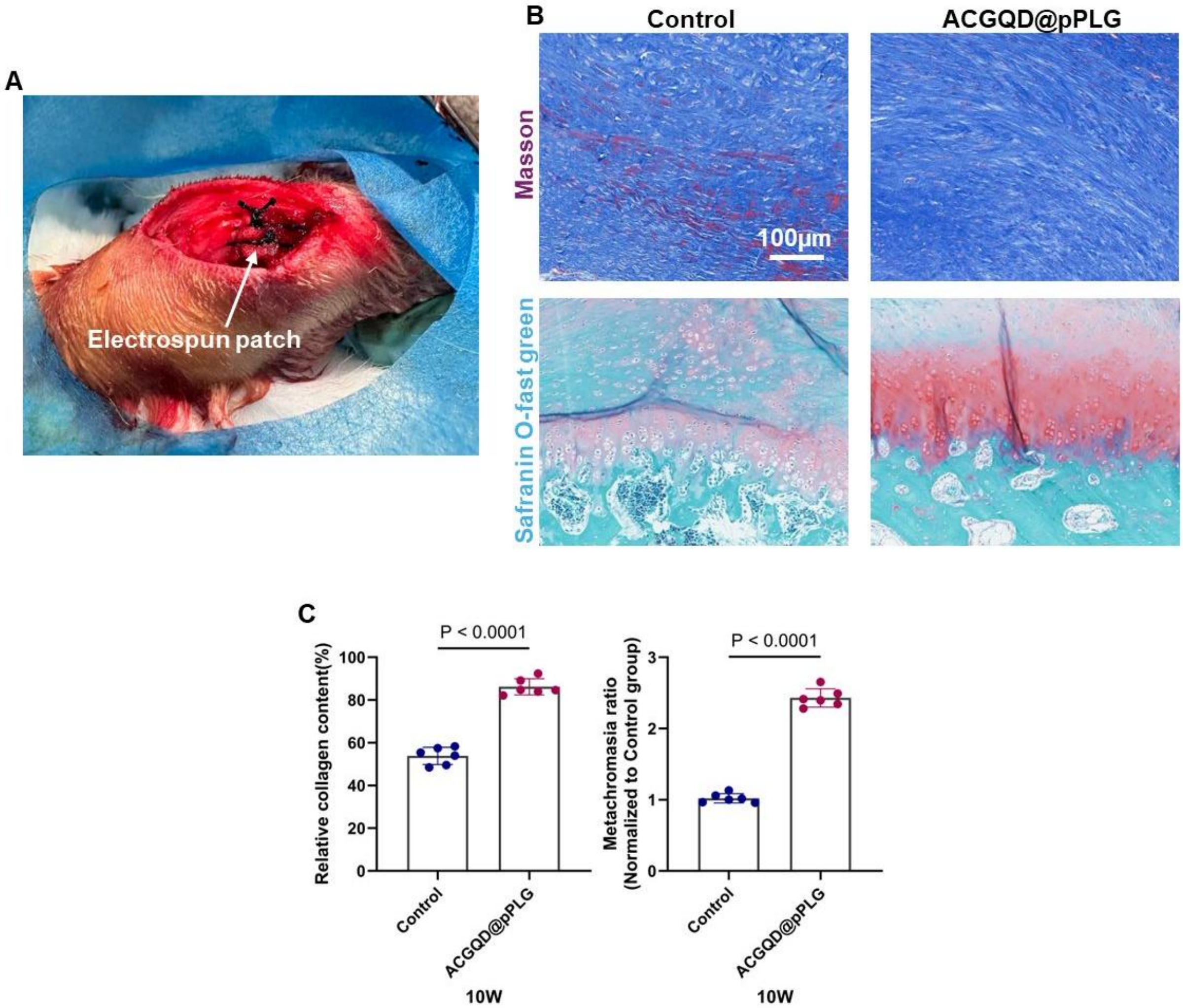

Figure S47. Histological assessment of rotator cuff repair in rabbits at 10 weeks. (A) Intraoperative photograph showing placement of the ACGQD@pPLG electrospun patch at the repaired tendon-to-bone interface. (B) Representative Masson trichrome-stained sections (top) and Safranin O–fast green-stained sections (bottom) from the Control and ACGQD@pPLG groups. Scale bar, 100 μm. (C) Masson-based collagen content (left) and relative Safranin O staining normalized to the Control reference (right). Data are mean ± SD; *n* = 6 animals per group. Groups were compared using an unpaired two-sided Student's *t*-test; *P* values are shown.

Table S1. Histological criteria for the modified tendon healing score (mTHS). Inflammatory cell infiltration, tenocyte alignment, collagen organization, and cellularity are each assigned a score from 0 to 3. Higher scores indicate less inflammatory cell infiltration and more organized tendon morphology (maximum total score, 12). PMN, polymorphonuclear neutrophils.

**Sub-domain (metric)**	**Score 0**	**Score 1**	**Score 2**	**Score 3**
Inflammatory cell infiltration	>50% dense PMN / lymphocytic infiltrate	25–50% multifocal aggregates, acute phenotype	5–25% scattered, predominantly mononuclear	<5% rare resident macrophages
Tenocyte alignment	<25% predominantly rounded fibroblasts	25–50% mixed rounded / elongated	50–75% mostly elongated, partial alignment	>75% uniform spindle-shaped tenocytes
Collagen organization	<25% disorganized, no preferred direction	25–50% patchy, focal organized regions	50–75% partial axial alignment	>75% densely aligned, near-native architecture
Cellularity (cells/mm^2)	>2000 hypercellular, granulation-like	1000–2000 moderately hypercellular	500–1000 transitioning to organized matrix	<500 near-native tendon cellularity

350 **Table S2. Primary antibodies used in this study, including target or conjugate, supplier,**
351 **and catalog number.**

Antibody Name	Company	Catalog Number
iNOS	Abcam	ab178945
Arg-1	Cell Signaling Technology	#93668
CD68	Proteintech	25747-1-AP
CD206	Abcam	ab64693
CD86	Abcam	ab239075
CD163	Abcam	ab182422
TNF-α	Proteintech	17590-1-AP
IL-10	Proteintech	60269-1-Ig
β-actin	Proteintech	66009-1-Ig
APC-CD68	BioLegend	333810
FITC-CD86	BioLegend	374203
PE-CD206	BioLegend	321105
PE-iNOS	Invitrogen	12-5920-82
HO-1	Abcam	ab68477
NRF2	Servicebio	GB113808-100
NQO1	Servicebio	GB15282-100
KEAP1	Abcam	ab139729
SCX	Santa Cruz Biotechnology	sc-87425
RUNX2	Proteintech	20700-1-AP

Antibody Name	Company	Catalog Number
COL I	Servicebio	GB124197-100
COL II	Servicebio	GB11021-100
COL III	Servicebio	GB111629-100
OCN	Proteintech	23418-1-AP
CD44	Proteintech	15675-1-AP
CD90	Proteintech	27178-1-AP
PI3K	Cell Signaling Technology	#4292
p-PI3K	Cell Signaling Technology	#4228
AKT	Cell Signaling Technology	#9272
p-AKT	Cell Signaling Technology	#4060
GSK3β	Cell Signaling Technology	#9315
p-GSK3β	Cell Signaling Technology	#9336
ASS1	Proteintech	16210-1-AP
4-HNE	Abcam	ab46545
FAK	Proteintech	66258-1-Ig
Calreticulin	Proteintech	27298-1-AP
CD47	Proteintech	20305-1-AP
GAPDH	Proteintech	60004-1-Ig
Lamin B1	Proteintech	12987-1-AP

Antibody Name	Company	Catalog Number
Na^+/K^+-ATPase	Proteintech	14418-1-AP

Table S3. Forward and reverse primer sequences used for reverse-transcription quantitative PCR (RT-qPCR) in human (Homo sapiens) and rat (Rattus norvegicus) samples.

Gene	Species		Primer sequence
GAPDH	*Homo sapiens*	Forward	GGAAGCTTGTCATCAATGGAAATC
		Reverse	TGATGACCCTTTTGGCTCCC
TNF	*Homo sapiens*	Forward	CCTCTCTCTAATCAGCCCTCTG
		Reverse	GAGGACCTGGGAGTAGATGAG
NOS2	*Homo sapiens*	Forward	TTCAGTATCACAACCTCAGCAAG
		Reverse	TGGACCTGCAAGTTAAAATCCC
ARG1	*Homo sapiens*	Forward	GTGGAAACTTGCATGGACAAC
		Reverse	AATCCTGGCACATCGGGAATC
IL10	*Homo sapiens*	Forward	GAGAACAGCTGCACCCACTTC
		Reverse	GGGCATCACCTCCTCCAGGTAA
NQO1	*Homo sapiens*	Forward	GAAGAGCACTGATCGTACTGGC
		Reverse	GGATACTGAAAGTTCGCAGGG
GCLC	*Homo sapiens*	Forward	GTTCTTGAAACTCTGCAAGAGAAG
		Reverse	ATGGAGATGGTGTATTCTTGTCC
ASS1	*Homo sapiens*	Forward	TTATAACCTGGGATGGGCACC
		Reverse	TGGACATAGCGTCTGGGATTG
ASL	*Homo sapiens*	Forward	CTATGACCGGCATCTGTGGAA
		Reverse	CTTGGTGCAGTAGAGGATGAGG
Gapdh	*Rattus norvegicus*	Forward	CTGGAGAAACCTGCCAAGTATG
		Reverse	GGTGGAAGAATGGGAGTTGCT
Spp1	*Rattus*	Forward	GATGAACAGTATCCCGATGCCA

Gene	Species		Primer sequence
	norvegicus	Reverse	GTCTTCCCGTTGCTGTCCTGA
Runx2	*Rattus norvegicus*	Forward	CCCAACTTCCTGTGCTCCGT
		Reverse	AGTGAAACTCTTGCCTCGTCC
Col1a1	*Rattus norvegicus*	Forward	TGCCCAGAAGAATATGTATCACCA
		Reverse	AGCAAAGTTTCCTCCAAGACCAG
Tnmd	*Rattus norvegicus*	Forward	AACAAATCGTAGCACGGGAG
		Reverse	AGTCGGCTAACAGATGCCAG
Scx	*Rattus norvegicus*	Forward	TCCGACGAGAAACCCTGC
		Reverse	GCAGCGTCTCAATCTTGGAG
